# Global Prediction of Dengue Incidence Using an Explainable Artificial Intelligence‐Driven ConvLSTM Integrating Environmental, Health, and Socio‐Economic Determinants

**DOI:** 10.1002/hsr2.72280

**Published:** 2026-04-06

**Authors:** Md. Abu Bokkor Shiddik

**Affiliations:** ^1^ Department of Statistics Begum Rokeya University Rangpur Bangladesh

**Keywords:** deep learning, feature attribution, public health forecasting, spatiotemporal modeling, vector‐borne diseases

## Abstract

**Background and Aims:**

Dengue fever is a rapidly expanding vector‐borne disease that poses significant global epidemiological and public health challenges. Accurate and interpretable forecasting is essential for timely interventions, yet most models overlook spatiotemporal, sex‐specific, and country‐level heterogeneity in disease dynamics. This study aimed to develop a robust explainable AI (XAI) framework to predict dengue incidence globally and identify key environmental, health system, and socio‐economic drivers.

**Methods:**

A Convolutional Long Short‐Term Memory (ConvLSTM) network was applied to predict dengue incidence across 118 countries from 2000 to 2021. The data set included total, male, and female dengue incodence alongside 20 climatic, environmental, health system, and socio‐economic predictors. The model was trained using data from 2000 to 2018 and tested on 2019–2021. Model performance was evaluated using RMSE, MAE, *R*², and adjusted *R*². Feature contributions were assessed through multiple XAI approaches: SHAP values, permutation importance, ±50% perturbation sensitivity perturbations, integrated gradients (IG), and layer‐wise relevance propagation (LRP).

**Results:**

ConvLSTM achieved the best predictive performance (*R*² = 0.7731), demonstrating suitability for national‐level public health planning. Sex‐specific analysis revealed that annual freshwater withdrawals (SHAP: 44.37%; IG: 0.41; LRP: 0.38) dominated male dengue incidence, whereas hospital bed density had a greater influence for females (SHAP: 31.86%; IG: 0.34; LRP: 0.32). Temperature anomalies contributed consistently to both sexes (SHAP: 11.51%; IG: 0.18; LRP: 0.17). Country‐level contributions highlighted electricity access (India: 97.35%; Bangladesh: 89.62%) and UHC coverage (Bangladesh: 8.33%) as key socio‐economic determinants, with environmental and healthcare factors such as community health resources (Afghanistan: 35.42%; Brazil: 9.00%) further shaping sex‐specific patterns. Sensitivity analysis indicated dengue incidence varied from −65% to +91% under ±50% predictor perturbations, underscoring model responsiveness and targeted intervention potential.

**Conclusion:**

By integrating SHAP, IG, and LRP, the ConvLSTM–XAI framework provides transparent, sex‐aware, and country‐specific dengue forecasts. The results support targeted, climate‐resilient, and equitable dengue control strategies.

## 1 Introduction

Dengue fever is still one of the fastest‐growing mosquito‐borne diseases in the world, and outbreaks have become worse over the past decade. Global cases jumped from less than 3 million in 2015 to more than 7.6 million in 2024, including about 3.4 million confirmed infections and over 3000 deaths [1, 2]. In 2025, the European Centre for Disease Prevention and Control (ECDC) reported more than 4 million dengue cases and 2500 deaths across 101 countries, with the highest numbers in the Americas, South Asia, and parts of Africa [1, 2, 3]. Some national outbreaks have been especially severe. Brazil recorded over 2.8 million cases, the largest in its history, while Bangladesh, India, Indonesia, the Philippines, Vietnam, Thailand, Sri Lanka, Malaysia, and Peru also faced major surges linked to unusual weather, rapid urbanization, and weak vector control systems [4].

There isn't much gender‐based data available, but what we do know shows that dengue affects both men and women almost equally. In Bangladesh, hospital reports from 2025 found that around 60.9% of patients were male and 39.1% were female [5, 6]. This modest difference could be due to similar exposure to mosquito habitats and limited access to proper healthcare, especially in crowded urban areas [7].

Countries around the world have launched emergency efforts to control dengue; large‐scale mosquito control drives, hospital mobilization, and awareness campaigns are now common [1]. Bangladesh introduced its National Dengue Prevention and Control Strategy (2024–2030), focusing on better surveillance, improved hospital readiness, and stronger community participation [8]. Still, most responses are reactive. They tend to rely on case numbers and weather data after outbreaks have already started [1, 9].

Existing dengue prediction models also have limitations. Many work with broad time intervals and don't fully capture how climate, environment, and social factors interact over time, which makes predictions less accurate across different regions [10]. On top of that, data from health, climate, and infrastructure sectors are often scattered, making it hard to act quickly [11]. Few forecasting tools take into account differences in healthcare quality or social vulnerability, meaning they often miss key equity issues [12].

To fill these gaps, this study builds a reliable, easy‐to‐understand, and inclusive dengue prediction model based on One Health and health equity principles. The One Health idea connects human, environmental, and vector health—important because dengue transmission depends on factors like temperature, humidity, rainfall, land use, and how people interact with their surroundings [13, 14]. Adding a health equity focus helps ensure that differences in healthcare access, education, and infrastructure are also considered [15, 16].

This study brings together national‐level data across four areas: (1) dengue incidence, separated by total population, males, and females; (2) climate and environmental conditions; (3) health system indicators, like hospital bed and doctor availability; and (4) socioeconomic factors, including GDP growth, education, and electricity access. Twenty predictors were selected based on prior evidence, biological relevance, and data availability across all 118 countries. Factors such as vector control coverage or vaccination rates were not included due to insufficient or inconsistent global data. By linking these areas, the model aims to give a clearer, more complete picture of dengue risk—helping public health officials plan ahead, respond faster, and build fairer, climate‐resilient health systems in regions where dengue is common.

### 1.1 Novelty of the Study

This study presents a global ConvLSTM‐based framework designed to predict dengue incidence across 118 countries between 2000 and 2021 using 20 different climatic, environmental, health system, and socio‐economic factors. Unlike traditional models, it can capture how dengue patterns change over time and space, while also accounting for differences between males and females and variations across countries. The analysis highlights several key factors that consistently influence dengue trends, including annual freshwater withdrawals, hospital bed availability, and access to electricity.

By combining this deep learning approach with explainable AI tools, the framework not only forecasts dengue more accurately but also shows why certain predictions occur. These insights make it easier for policymakers and health officials to design targeted interventions, prioritize resources, and strengthen climate‐resilient public health strategies in regions where dengue remains a major threat.

## 2 Methods and Methodology

### 2.1 Study Area and Data Collection

This study looked at dengue incidence in 118 countries across Asia, Africa, Oceania, and the Americas from 2000 to 2021. Each country was treated as one observation unit, and the 22‐year timespan allowed us to track how dengue trends have changed and evolved over time.

Data on dengue cases—reported per 1 million people and separated by total population, males, and females—were collected from the Global Health Data Exchange (GHDx) [17]. To understand what might be driving these trends, 20 predictor variables were gathered from trusted international sources such as the World Bank [18], World Health Organization (WHO) [19], United Nations (UN) [20], and Our World in Data [21] (Table 1). These variables cover four key areas: climate, environment, health systems, and socioeconomic conditions. More details on why each variable was included, and how it relates to dengue transmission, are provided in the Supplementary Information (See Supporting Information S1: Rationale for variable selection).

**Table 1:** Summary of variables, codes, and data sources for dengue incidence analysis across 118 countries (2000–2021).

Category	Variable	Code	Source
Spatio‐temporal	Country (118)	Country	
	Year (2000–2021)	Year	
Dengue	Global dengue incident total (per 1 M people)	Incidence both	Global health data exchange (GHDx) [17]
	Global dengue incidence in males (per 1 M people)	Incidence male	Global health data exchange (GHDx) [17]
	Global dengue incidence in females (per 1 M people)	Incidence female	Global health data exchange (GHDx) [17]
Climatic and environmental factors	Relative humidity (%)	x1	World Bank [18]
	Days with precipitation over 20 mm	x2	World Bank [18]
	Temperature anomaly (°C)	x3	Our world in data [21]
	Average annual surface temperature (°C)	x4	Our world in data [21]
	Agricultural land (% of land area)	x5	World Bank [18]
	Air pollution	x6	World Bank [18]
Health system and health risk factors	Hospital bed density (per 10,000 population)	x7	World Bank [18]
	Density of physicians (per 10,000 population)	x8	World Health Organization (WHO) [19]
	Domestic general government health expenditure (%)	x9	World Health Organization (WHO) [19]
	Life expectancy at birth (years)	x10	World Health Organization (WHO) [19]
	Mortality rate under 5 per 1000 live births	x11	World Bank [18]
	UHC service coverage index (SDG 3.8.1)	x12	World Health Organization (WHO) [19]
Socioeconomic and demographic factors	Population, total	x13	World Bank [18]
	GDP growth (annual %)	x14	World Bank [18]
	Population growth (annual %)	x15	World Bank [18]
	Access to electricity	x16	World Bank [18]
	Average number of years adults aged 25 and older have spent in formal education.	x17	Our world in data [21]
	Crude rate of net migration	x18	United Nations (UN) [20]
	Urban population (% of total population)	x19	United Nations (UN) [20]
	Annual freshwater withdrawals, total (billion cubic meters)	x20	World Bank [18]

### 2.2 Data Processing and Statistical Analyses

Missing values in the dataset ranged from 0.5% to 7.3% across predictors. To handle missingness, K‐nearest neighbor (KNN) imputation methods were applied using *k* = 5 and Euclidean distance after standardizing predictors to zero mean and unit variance in Python using *pandas*, *sklearn*.*impute* packages [22]. To assess the robustness of imputation, multiple imputation by chained equations (MICE) and linear interpolation were also performed as alternative approaches. Model performance was compared using 1000 bootstrap resamples, demonstrating minimal variation in RMSE (±1.2%) and MAE (±0.9%), supporting the stability of KNN‐based imputation. All predictors were standardized to facilitate model convergence and ensure comparability across variables with different scales. Descriptive statistics were computed to summarize variable distributions, while temporal trends were visualized using line plots and heatmaps. Multicollinearity among predictors was assessed using variance inflation factors (VIFs) (Supporting Information S1: Table S1). While several predictors exhibited VIF > 3 (e.g., x17 = 3.28, x20 = 2.24), they were retained because deep learning models can robustly learn non‐linear interactions among correlated features without biasing predictions, unlike linear regression models. Descriptive statistics summarized variable distributions, while temporal trends were visualized using line plots and heatmaps. Spatial visualizations and maps were generated using the *naturalearth* dataset in combination with the *sf* package in RStudio. All analyses were conducted using R (version 5.4.1) [23] and Python 3.14.0 (IPython 9.6.0) [24] using standard packages for data manipulation and visualization.

### 2.3 Deep Learning Model Development, and Evaluation

**Figure 1:**
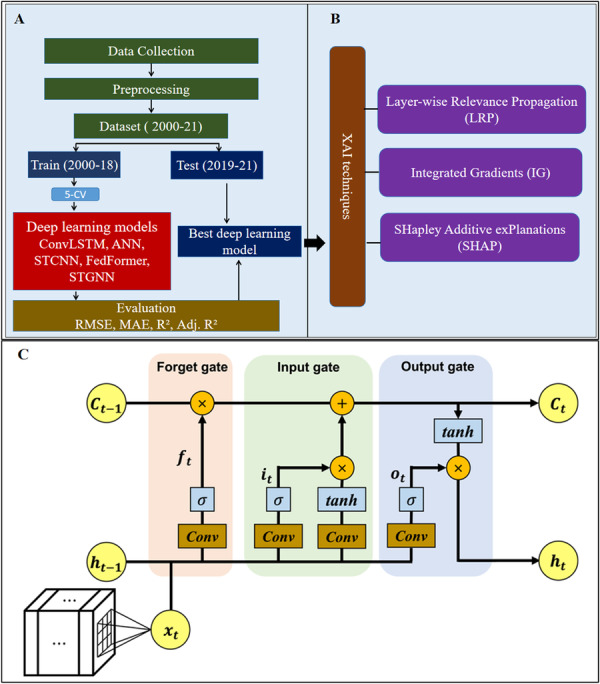
Study design. (A) Modelling workflow, (B) XAI techniques, (C) ConvLSTM architecture.

To predict dengue incidence across 118 countries from 2000 to 2021, I implemented five deep learning models (Convolutional Long Short‐Term Memory (ConvLSTM), Artificial Neural Network (ANN), Spatio‐Temporal Convolutional Neural Network (STCNN), Feedforward Neural Network (FNN), and Spatio‐Temporal Graph Neural Network (STGNN)). The data set was split into a training set (2000–2018) and a test set (2019–2021) (see Supporting Information S1: Note on 2019–2021 testing period) (Figure 1A). Log‐transformed dengue incidence was used for model training, with inverse transformation applied for evaluation metrics.

All models were trained using the Adam optimizer with learning rate = 0.001, batch size = 16, and mean squared error loss. Early stopping with patience = 100 max epochs was applied on validation folds during five‐fold cross‐validation. Hyperparameters were tuned via grid search across hidden sizes {32, 64, 128}, dropout {0.1, 0.2, 0.3}, and learning rates {0.0005, 0.001, 0.005}, selecting the configuration that minimized mean RMSE on validation folds. The models were implemented using Python 3.14 with the following packages: *pandas* for data manipulation, *numpy* for numerical operations, *scikit‐learn* for preprocessing, scaling, and evaluation metrics, *PyTorch* for deep learning model construction and training, *torchvision* (when convolutional layers were used), and *matplotlib* for visualization [25]. For graph‐based models (STGNN), *PyTorch* Geometric was used. Model performance was assessed using root mean squared error (RMSE), mean absolute error (MAE), *R*², and adjusted *R*², both on five‐fold cross‐validation of the training set and the independent test set (Figure 1 and Supporting Information S1: Table S2).

#### 2.3.1 Convolutional Long Short‐Term Memory (ConvLSTM)

The ConvLSTM integrates convolutional operations within LSTM cells to capture spatiotemporal dependencies among predictors. Input features (20 predictors) (Figure 1C and Supporting Information S1: Table S1) were reshaped into a 4 × 5 grid per country per time step to enable spatial convolution. The model comprised two stacked ConvLSTM layers with 32 hidden channels each, kernel size = 3 × 3, followed by flattening and a fully connected regression head with 64 neurons (ReLU activation, dropout = 0.2) and a linear output for log‐transformed incidence. The ConvLSTM cell operations are defined as:

$$i_{t}=\sigma (W_{xi}*X_{t}+W_{hi}*H_{t-1}+b_{i})$$

$$f_{t}=\sigma (W_{\mathrm{xf}}*X_{t}+W_{hf}*H_{t-1}+b_{f})$$

$$o_{t}=\sigma (W_{\mathrm{xo}}*X_{t}+W_{ho}*H_{t-1}+b_{o})$$

$$C_{t}=f_{t}⊙C_{t-1}+i_{t}⊙\tanh(W_{\mathrm{xc}}*X_{t}+W_{\mathrm{hc}}*H_{t-1}+b_{c})$$

$$H_{t}=o_{t}⊙\tanh(C_{t})$$

Where, * denotes convolution, ⊙ is element‐wise multiplication, σ is the sigmoid activation, $X_{t}$ is the input at a time *t*; W and b denote convolutional weights and biases [26, 27, 28].

#### 2.3.2 Artificial Neural Network (ANN) and Feedforward Neural Network (FNN)

ANN and FNN models contained three hidden layers (128, 64, 32 neurons) with *ReLU* activations and dropout (0.2) in the first two layers. The output layer used a linear activation to predict log‐transformed incidence (Supporting Information S1: Table S1). Formally:

$$h_{1}=\text{ReLU}({\text{XW}}_{1}+b_{1})$$

$$h_{2}=\text{ReLU}({h_{1}w}_{2}+b_{2})$$

$$h_{2}=\text{ReLU}({h_{2}W}_{3}+b_{3})$$

$$\hat{y}={h_{3}W}_{4}+b_{4}$$

Where X is the input vector, $h_{i}$ are hidden layer activations, $W_{i}\;,b_{i}$ are weights and biases, and $\hat{y}\;$is the predicted log‐incidence [29, 30].

#### 2.3.3 Spatio‐Temporal Convolutional Neural Network (STCNN)

The STCNN captured temporal and spatial dependencies by applying three 1D temporal convolutions (64, 32, 16 filters) followed by two 2D spatial convolutions (32, 16 filters), each with ReLU activation and dropout (0.2), before a fully connected layer of 32 neurons and a linear regression output:

$$\hat{y}=f_{\text{fc}}\left(\text{ReLU}\left(f_{\text{spatial}}\left(\text{ReLU}\left(f_{\text{temporal}}(X)\right)\right)\right)\right)$$

Where X is the feature matrix at time *t* for a country, $f_{\mathrm{temporal}}$ and $f_{\mathrm{spatial}}$ are temporal and spatial convolution operators, and $f_{\mathrm{fc}}$ is the fully connected layer [31, 32].

#### 2.3.4 Spatio‐Temporal Graph Neural Network (STGNN)

Each country was treated as a graph node, with edges representing spatial proximity or similarity in predictors. Temporal graph convolutions captured dynamics across years, followed by a fully connected layer of 32 neurons and a linear output:

$${\hat{y}}_{t}=f\left(\text{FC}\left(\text{TemporalGraphConv}\left(\text{GraphConv}\left(X_{t},A\right);f_{T}\right)\right)\right)$$

Where, $X_{t}$ denotes the feature matrix of all nodes at time t, A is the adjacency matrix representing the graph structure, $W_{T}$ corresponds to the temporal graph convolution kernels, FC is the fully connected layer, and f is the linear activation function at the output layer [33, 34].

#### 2.3.5 Model Performance Evaluation

Model evaluation metrics were calculated as:

$$\text{RMSE}=\sqrt{\frac{1}{n}\sum_{i=1}^{n}{\left({\hat{y}}_{i}-y_{i}\right)}^{2}},\text{MAE}=\frac{1}{n}\sum_{i=1}^{n}\left|{\hat{y}}_{i}-y_{i}\right|,R^{2}=1-\frac{\sum_{i=1}^{n}\left({\hat{y}}_{i}-y_{i}\right)}{\sum_{i}^{n}({\hat{y}}_{i}-\bar{y})}$$

Where, n denotes the number of observations and p the number of predictors.

#### 2.3.6 Feature Contribution and Sensitivity Analyses Using SHAP

SHapley Additive exPlanations (SHAP) and permutation‐based importance were used to quantify predictor contributions. SHAP values for feature i are:

$$\emptyset_{i}=\;\sum_{S\;\subseteq F\{i\}}\frac{\left|S\right|!\left(\left|F\right|-\left|S\right|-1\right)!}{\left|F\right|}\left[f_{s\cup \left\{i\right\}}\left(X_{S\cup \left\{i\right\}}\right)-\;f_{s}(x_{s})\right]$$

Where, F is the set of all features, S is a subset of features excluding i, $f_{s}\left(x_{s}\right)$denotes the model prediction using only features in S, and $\emptyset_{i}$ represents the contribution of feature i to the difference between the model output for xxx and the expected model output [35].

Permutation importance was computed at the country level by randomizing each top feature and measuring the increase in RMSE relative to baseline, normalized to 100% per country:

$${\text{Importance}}_{i}=\frac{\max({\bar{\text{RMSE}}}_{\text{perm}\left(i\right)}-{\text{RMSE}}_{\text{baseline},}0)}{\sum_{j=1}^{10}\max({\bar{\text{RMSE}}}_{\text{perm}\left(j\right)}-{\text{RMSE}}_{\text{baseline},}0)}$$

where ${\bar{\mathrm{RMSE}}}_{\mathrm{perm}\left(i\right)}$ is the average RMSE over multiple permutations of feature i [36].

Sensitivity analysis perturbed each top predictor by ±50% of its standardized value, computing the mean percent change in predicted dengue incidence:

$${△}_{y_{i}}=\;\frac{1}{n}\sum_{j=1}^{n}\frac{{\hat{y}}_{j}\left(x_{i}\left(1+p\right)\right)-{\hat{y}}_{j}(x_{i})}{{\hat{y}}_{j}(x_{i})}\times 100$$

where n is the number of observations in the test set, ${\hat{y}}_{j}(x_{i})$ is the baseline prediction for observation j, and ${\hat{y}}_{j}\left(x_{i}\left(1+p\right)\right)$ is the predicted incidence after perturbing feature i by p [37, 38].

These analyses enabled both global and country‐level interpretation of feature importance, cross‐country comparison, and quantification of model sensitivity, highlighting predictors with the largest influence on dengue incidence.

#### 2.3.7 Feature Contribution Analysis Using Integrated Gradients (IG)

Integrated Gradients (IG) is a gradient‐based attribution method used to interpret ConvLSTM predictions and quantify feature contributions. IG measures the effect of each input feature by integrating the gradients along a straight path from a baseline input (all‐zero vector) to the actual input:

$${\text{IG}}_{i}(x)=(x_{i}-x_{i}^{\prime })\times \int_{0}^{1}\frac{\text{δF}(x^{\prime }+\text{α}(x-x^{\prime }))}{{\text{δx}}_{i}}d\alpha$$

Where F(.) is the ConvLSTM prediction function, x is the input feature vector, *x*′ is the baseline vector, and i indexes the feature. Signed attributions indicate whether a feature increases (positive) or decreases (negative) the predicted dengue incidence.

Feature attributions were averaged across the test set to obtain global importance. Layer‐wise analysis was performed by extracting attributions at the input layer, hidden layer, and fully connected output layer, providing a multi‐layer understanding of feature relevance. Visualization employed diverging lollipop plots, highlighting the magnitude and direction of contributions for the top predictors.

#### 2.3.8 Layer‐Wise Relevance Propagation (LRP)

Layer‐wise Relevance Propagation (LRP) was applied to decompose ConvLSTM predictions across network layers while preserving the total output at each stage. For a model F(.) and input x, the relevance scores $R_{i}$ satisfy:

$$\sum_{i}R_{i}=F(x)$$

Relevance is propagated backward through the network, redistributing contributions proportionally to each neuron's input. LRP allows interpretation of feature contributions at multiple levels:

Input layer: direct attribution of original predictors. Positive values indicate features that enhance predicted incidence; negative values indicate suppressing effects.

A. Hidden layer: aggregated contribution of ConvLSTM hidden states, revealing the most informative latent spatio‐temporal representations.
B. Fully connected layer: weighted contribution of the final hidden representations, summarizing high‐level interactions among features.

Relevance scores were normalized to percentages for cross‐layer comparison. The top 10 features were identified based on the absolute magnitude of contributions, and diverging lollipop plots were used to visualize positive and negative contributions, enabling clear interpretation of enhancing and suppressing effects on dengue incidence.

## 3 Results

### 3.1 Global Dengue Incidence Overview

**Figure 2:**
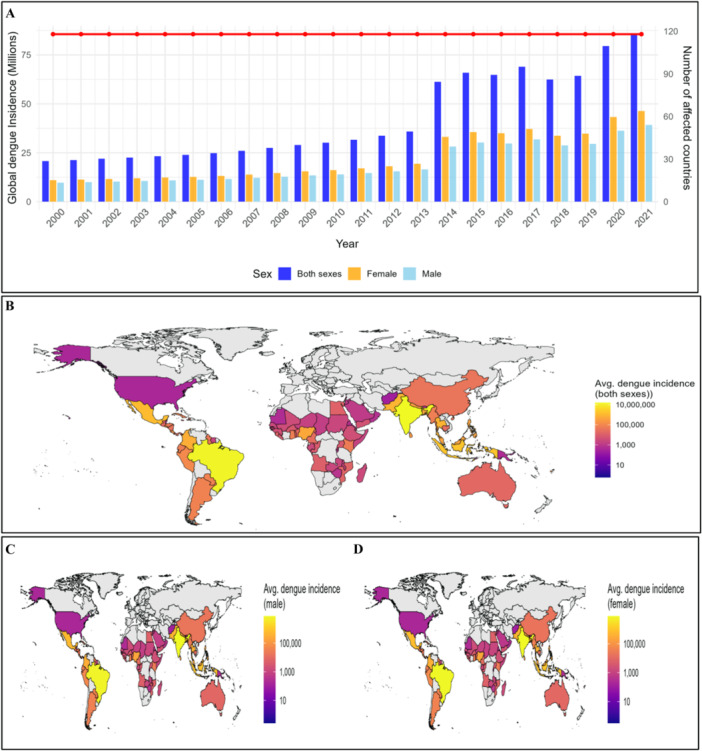
Country‐wise average dengue incidence across 190 countries from 2000 to 2021: (A) Average dengue incidence for both sexes combined, showing the overall burden across countries. (B) Average dengue incidence among males, highlighting sex‐specific patterns of disease distribution. (C) Average dengue incidence among females, illustrating differences compared to males and combined totals. Incidence values are presented as the mean number of cases per year per country, calculated from 2000 to 2021. Countries with missing data are shown in gray.

From 2000 to 2021, dengue incidence exhibited substantial geographic and sex‐specific variation (Figure 2; Supporting Information S1: Tables S2–S5). High‐burden countries included India (male 7,533,494 ± 3,730,613; female 8,106,364 ± 4,112,545), Bangladesh (male 197,882 ± 106,798; female 238,385 ± 138,110), Viet Nam (male 317,766 ± 90,588; female 391,530 ± 121,645), and the Philippines (male 180,694 ± 165,404; female 215,738 ± 201,522). Latin America also reported high incidence, with Brazil (male 6,191,454 ± 3,399,890; female 7,687,881 ± 4,273,945) and Colombia (male 294,453 ± 169,544; female 372,662 ± 212,828) (Figure 2; Supporting Information S1: Tables S2–S5).

Low‐incidence countries included Afghanistan (male 112.63 ± 36.18; female 125.29 ± 40.88), Jordan (male 515 ± 497; female 567 ± 559), and Niue (male 0.26 ± 0.07; female 0.32 ± 0.10) (Supporting Information S1: Tables S4–S5). Some countries showed extreme variability, such as Cabo Verde (male 171,034 ± 343,174; female 195,964 ± 393,517) and Mauritius (male 133,238 ± 473,578; female 199,911 ± 712,536). Quartile differences further highlighted outbreak peaks, for example, Argentina males (median 20,448; Q3 36,606) and females (median 25,453; Q3 49,125) (Supporting Information S1: Tables S4–S6).

Across most countries, female incidence slightly exceeded male incidence. Dengue burden was concentrated in tropical and subtropical regions, reflecting pronounced spatial heterogeneity and episodic outbreaks. Socio‐demographic, climate, and environmental predictors also varied widely, indicating potential drivers of observed incidence patterns (Figure 2; Supporting Information S1: Table S2–S8).

### 3.2 Model Performance in Predicting Dengue Incidence

ConvLSTM consistently outperformed other models in predicting dengue incidence across sexes. It achieved the highest predictive accuracy with *R*² values of 0.7731 (total), 0.7753 (male), and 0.8877 (female), and the lowest RMSE and MAE for each group (total: RMSE = 946,837.00, MAE = 158,791.17; male: RMSE = 451,397.78, MAE = 73,207.33; female: RMSE = 364,037.02, MAE = 109,361.03). FedFormer and STCNN showed moderate performance across all groups (*R*² = 0.5207–0.6978), whereas ANN and STGNN had the weakest predictive strength (*R*² = –0.0120 to 0.4584) (Table 2, Supporting Information S1: Table S9). At the global scale, an *R*² of 0.7731 suggests adequate generalizability for population‐level public health monitoring and comparative risk assessment across countries, rather than fine‐grained local forecasting.

**Table 2:** Average performance comparison of deep learning models (ConvLSTM, ANN, STCNN, FedFormer, and STGNN) in predicting dengue incidence across both sexes, males, and females from 2000 to 2021.

Model	RMSE	*R*²	Adj. *R*²	MAE
**Both**				
ConvLSTM	946,837.00	0.7731	0.7709	158,791.17
ANN	1,469,069.49	0.4269	0.4099	389,064.68
STCNN	1,215,981.46	0.6298	0.6243	180,316.60
FedFormer	1,128,745.43	0.6978	0.6907	291,042.91
STGNN	2,050,098.26	0.0032	−0.0137	276,471.93
**Male**				
ConvLSTM	451,397.78	0.77529	0.77331	73,207.33
ANN	672,486.97	0.45836	0.44232	177,615.82
STCNN	574,651.85	0.6208	0.61433	82,491.61
FedFormer	654,714.19	0.54438	0.53594	119,081.87
STGNN	976,552.76	−0.01203	−0.02918	131,333.99
**Female**				
ConvLSTM	364,037.02	0.88772	0.8852	109,361.03
ANN	767,567.10	0.44281	0.42631	207,274.02
STCNN	564,590.94	0.69806	0.69762	131,202.78
FedFormer	731,633.98	0.52074	0.50709	109,169.60
STGNN	1,083,314.53	0.00033	−0.01663	148,412.63

Abbreviations: Adj. R² = adjusted coefficient of determination, ANN = artificial neural network, ConvLSTM = convolutional long short‐term memory, FedFormer = federated transformer, MAE = mean absolute error, MAPE = mean absolute percentage error, *R*² = coefficient of determination, RMSE = root mean square error, STCNN = spatiotemporal convolutional neural network, STGNN = spatiotemporal graph neural network.

#### 3.2.1 ConvLSTM Hidden Layer, Activation, and Attention Patterns

The ConvLSTM model captured temporal dynamics for countries from 2019 to 2021. Attention weights increased over time, indicating stronger contributions from later steps. For instance, Antigua and Barbuda (2019) showed attention rising from 0.036 at t1t_1t1 to 0.468 at t5t_5t5, Brazil (2019) from 0.036 to 0.456, and India (2019) from 0.034 to 0.398.

Hidden state means and activation statistics reflected temporal feature learning. Early steps had low hidden means (≈0.004–0.006) and small activations (≈0.004–0.015), while later steps reached maxima close to 1 (e.g., ActMax t5t_5t5 ≈ 0.998–0.999), indicating strong feature encoding.

Overall, the model consistently emphasized later time steps across countries, highlighting its effectiveness in capturing temporal dependencies in the data (Supporting Information S1: Figures S1, S2, and Table S10).

### 3.3 Global and Country‐Level Feature Contributions and Sensitivity Analysis on Dengue Prediction

#### 3.3.1 Global Feature Contributions on Dengue Prediction

**Figure 3:**
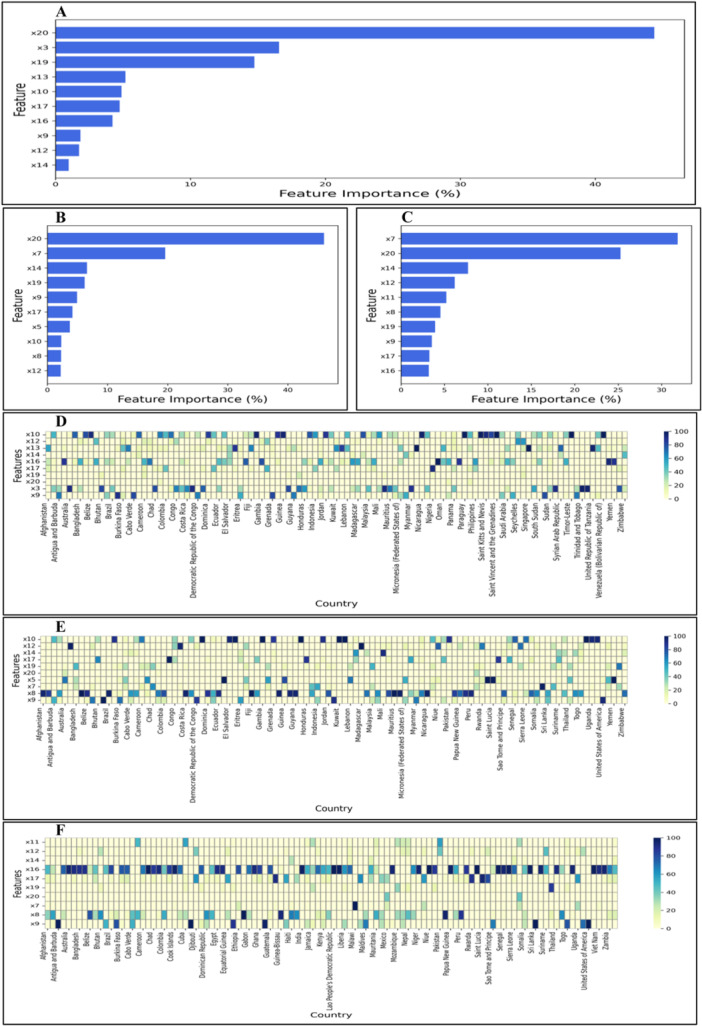
Top 10 influential SHAP features driving dengue incidence prediction using the ConvLSTM model for (A) both sexes combined, (B) males, and (C) females. Panels (D–F) depict country‐specific heatmaps illustrating the relative contribution of each feature to dengue prediction across regions for (D) both sexes, (E) males, and (F) females, respectively. x3: temperature anomaly; x5: agricultural land (% of land area); x7: hospital bed density (per 10,000 population); x8: density of physicians (per 10,000 population); x9: domestic general government health expenditure (%); x10: life expectancy at birth (years); x11: mortality rate under 5 per 1000 live births; x12: UHC service coverage index (SDG 3.8.1); x13: population, total; x14: GDP growth (annual %); x16: access to electricity; x17: average number of years adults aged 25+ spent in formal education; x19: urban population (% of total population); x20: Annual freshwater withdrawals, total (billion cubic meters).

Across all countries, annual freshwater withdrawals emerged as the most influential predictor, contributing 44.37% to global dengue incidence predictions (Figure 3, Supporting Information S1: Figure S3, Tables S11–S14). For males, the leading contributors were freshwater withdrawals (46.01%), hospital bed density (19.62%), and GDP growth (6.58%), while for females, hospital bed density (31.86%) and freshwater withdrawals (25.27%) were the primary factors. Additional important contributors included urban population (14.74%), life expectancy (4.88%), access to electricity (4.23%), education (4.76%), and UHC coverage (1.74–4.94%) (Supporting Information S1: Table S11).

The SHAP correlation matrices (Supporting Information S1: Tables S12–S14) provide additional context for these global contributions. In total dengue incidence (Supporting Information S1: Table S12), freshwater withdrawals (x20) were strongly correlated with GDP growth (x14, *r* = 0.567) and urban population (x19, *r *= 0.474), reinforcing the high influence of these socio‐economic and environmental factors. In males (Supporting Information S1: Table S13), freshwater withdrawals (x20) correlated strongly with hospital bed density (x7, *r *= 0.952), which aligns with the observed top contributors for male dengue incidence. For females (Supporting Information S1: Table S14), hospital bed density (x7) was moderately correlated with UHC coverage (x12, *r* = 0.661) and electricity access (x16, *r *= −0.096), supporting the observed feature rankings.

These results highlight the strong influence of environmental, health system, and socio‐economic factors on global dengue incidence, demonstrating opportunities to guide targeted interventions across diverse populations.

#### 3.3.2 Country‐Level Feature Contributions on Dengue Prediction

Feature contributions demonstrated notable diversity across countries. In India, access to electricity (97.35%) and temperature anomaly (2.62%) were the leading predictors, while in Bangladesh, electricity access (89.62%) and UHC coverage (8.33%) were most influential. In Afghanistan, electricity access (48.05%) and community health resources (35.42%) played a central role, whereas in Brazil, electricity access (81.74%) and community health resources (9.00%) were dominant. In the Philippines, electricity access (87.03%) and temperature anomaly (11.51%) were the primary contributors (Supporting Information S1: Table S15). African countries exhibited diverse contributions from infrastructure, environmental, and health system indicators, reflecting the heterogeneous drivers of dengue incidence across the continent. Sex‐specific patterns were evident: male incidence was primarily influenced by electricity access, temperature anomaly, life expectancy, and freshwater availability, while female incidence was mainly shaped by electricity access, community health resources, UHC coverage, and temperature anomalies (Figure 3; Supporting Information S1: Tables S16–S17).

#### 3.3.3 Sensitivity Analysis of the Top 10 Features for ConvLSTM Dengue Prediction

**Figure 4:**
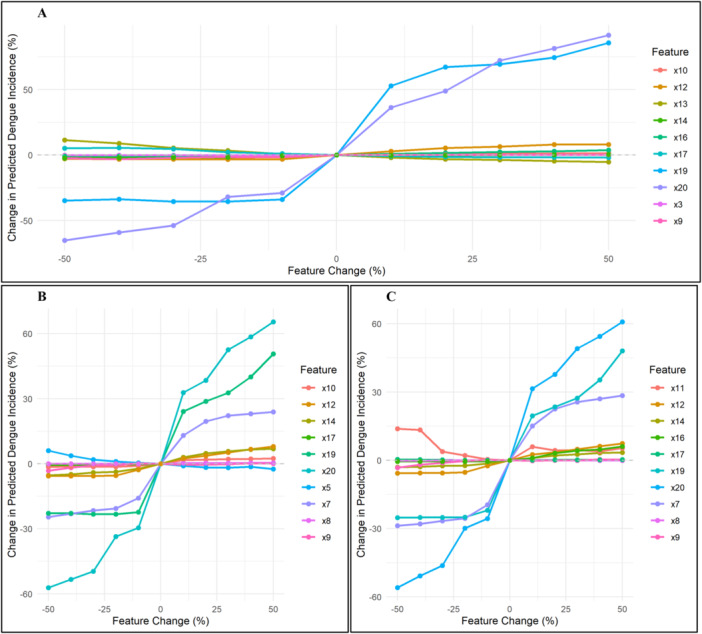
Sensitivity analysis of the top 10 predictors of dengue incidence using the ConvLSTM model for (A) both sexes combined, (B) males, and (C) females. The plots show the effect of varying each feature on predicted dengue cases, highlighting the relative impact and direction of change for each predictor. x3: temperature anomaly; x5: agricultural land (% of land area); x7: hospital bed density (per 10,000 population); x8: density of physicians (per 10,000 population); x9: domestic general government health expenditure (%); x10: life expectancy at birth (years); x11: mortality rate under 5 per 1000 live births; x12: UHC service coverage index (SDG 3.8.1); x13: population, total; x14: GDP growth (annual %); x16: access to electricity; x17: average number of years adults aged 25+ spent in formal education; x19: urban population (% of total population); x20: annual freshwater withdrawals, total (billion cubic meters).

Sensitivity analyses, evaluating ±50% changes in top predictors, revealed the relative responsiveness of dengue incidence to key factors. Freshwater withdrawals, urban population, and total population exerted the greatest influence, producing variations of 65%–91% for both sexes, 57%–65% for males, and 56%–61% for females (Figure 4; Supporting Information S1: Tables S14–S16). Other important predictors, including GDP growth (up to 74.46%), life expectancy (up to 1.49%), education (up to 3.72%), UHC coverage (up to 8.07%), temperature anomaly (up to 0.06%), under‐5 mortality (up to 5.25%), and agricultural land (up to 0.44%), contributed meaningful variations to predicted incidence (Figure 4; Supporting Information S1: Tables S18–S20). These results highlight the strong positive impact of environmental, infrastructural, and socio‐economic factors on dengue incidence, with clear sex‐specific patterns, emphasizing opportunities for targeted interventions and resource prioritization.

#### 3.3.4 Complementary Explainability Analysis Using Information Gain and Layer‐Wise Relevance Propagation

**Figure 5:**
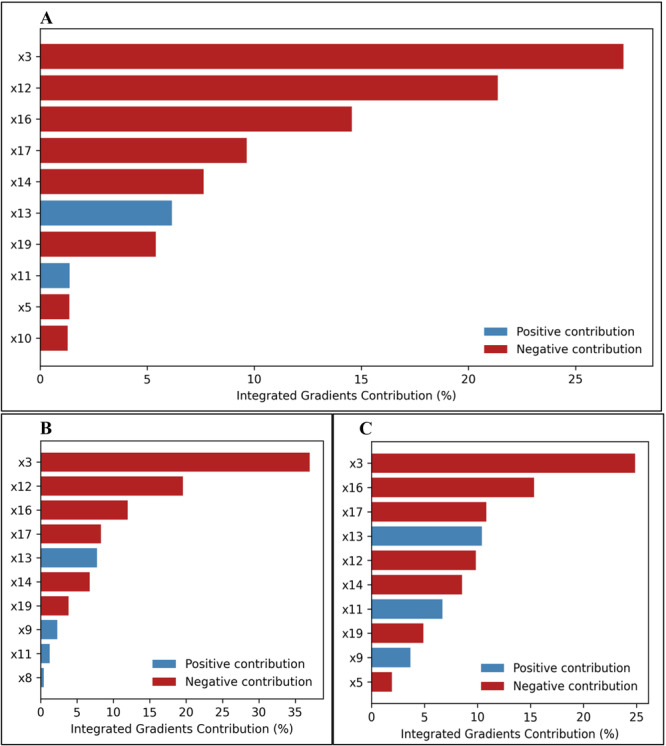
Top 10 influential IG features driving dengue incidence prediction using the ConvLSTM model for (A) both sexes combined, (B) males, and (C) females. x3: temperature anomaly; x5: agricultural land (% of land area); x7: hospital bed density (per 10,000 population); x8: density of physicians (per 10,000 population); x9: domestic general government health expenditure (%); x10: life expectancy at birth (years); x11: mortality rate under 5 per 1000 live births; x12: UHC service coverage index (SDG 3.8.1); x13: population, total; x14: GDP growth (annual %); x16: access to electricity; x17: average number of years adults aged 25+ spent in formal education; x19: urban population (% of total population); x20: annual freshwater withdrawals, total (billion cubic meters).

**Figure 6:**
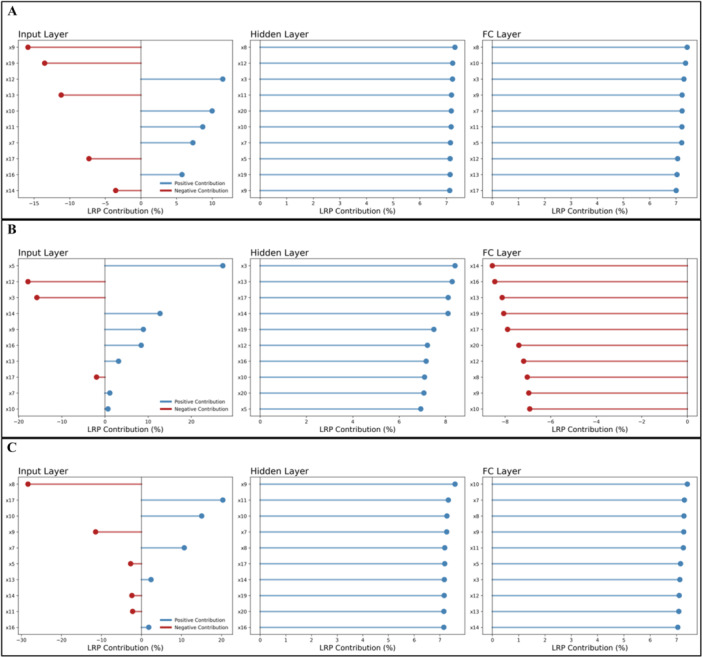
Layer‐wise Signed Feature Contribution (LRP)—ConvLSTM Model on dengue prediction, (A) both sexes, (B) male, (C) female; x3: temperature anomaly; x5: agricultural land (% of land area); x7: hospital bed density (per 10,000 population); x8: density of physicians (per 10,000 population); x9: domestic general government health expenditure (%); x10: life expectancy at birth (years); x11: mortality rate under 5 per 1000 live births; x12: UHC service coverage index (SDG 3.8.1); x13: population, total; x14: GDP growth (annual %); x16: access to electricity; x17: average number of years adults aged 25+ spent in formal education; x19: urban population (% of total population); x20: annual freshwater withdrawals, total (billion cubic meters).

To complement the SHAP‐based results, we evaluated feature importance using signed Information Gain (IG) and Layer‐wise Relevance Propagation (LRP) (Figures 5, 6 and Supporting Information S1: Table S21–S23). Across all models, temperature anomaly (IG: –27.24; LRP: 1.76) consistently emerged as a top predictor, highlighting its strong influence on dengue incidence globally. Other key predictors included UHC coverage (IG: –21.37; LRP: 11.48), access to electricity (IG: –14.56; LRP: 5.76), education (IG: –9.65; LRP: –7.29), urban population (IG: –5.40; LRP: –13.50), and GDP growth (IG: –7.64; LRP: –3.54), reflecting the combined effects of environmental, health system, and socio‐economic factors.

Sex‐specific patterns were evident. For males, temperature anomaly (G: –36.98; LRP: –15.77), freshwater withdrawals (IG: –0.63; LRP: –0.63), and hospital bed density (IG: 1.13; LRP: 6.93) were among the most influential, while for females, hospital bed density (IG: 6.70; LRP: 7.26), temperature anomaly (IG: –24.86; LRP: 0.21), and education (IG: –10.82; LRP: 20.34) dominated feature relevance (Figures 5, 6).

Layer‐wise analysis with LRP revealed that relevance scores were strongest at the input layer, with certain predictors exhibiting amplified sex‐specific contributions (Supporting Information S1: Table S21–S23). At the hidden and fully connected layers, relevance was more evenly distributed, indicating integration and abstraction of input features within the ConvLSTM network.

The observed consistency between SHAP, IG, and LRP top features reinforces the robustness of the identified drivers of dengue incidence. These complementary explainability methods provide convergent evidence for the importance of environmental, infrastructural, and socio‐economic predictors, thereby increasing confidence in the model interpretations (Figures 5, 6).

#### 3.3.5 Predicted Global Dengue Incidence, 2022–2032

The ConvLSTM model predicted global dengue incidence for 118 countries from 2022 to 2032, achieving *R*² = 0.81 (Supporting Information S1: Table S23). The highest predicted incidences were observed in India (658.10 cases per 1 M), China (549.25), Maldives (361.79), Cuba (338.46), Saudi Arabia (338.12), Lebanon (295.93), Indonesia (270.80), Singapore (259.56), Malaysia (254.01), and Colombia (253.44). Other countries with substantial predicted incidence included Philippines (205.06), Bangladesh (204.97), Guatemala (205.16), Brazil (199.62), and Costa Rica (197.01), highlighting regions with the highest dengue burden in the next decade (Supporting Information S1: Table S24).

## 4 Discussion

This study offers a broad assessment of dengue across 118 countries from 2000 to 2021. Clear differences were observed across regions, time periods, and between males and females. Dengue burden was highest in tropical and subtropical areas, where warm temperatures, rapid urban growth, and limited infrastructure create favorable conditions for mosquito breeding and virus transmission [2, 3, 39, 40].

Among the models tested, ConvLSTM gave the most accurate predictions. It captured both spatial patterns and temporal trends, allowing delayed and cumulative effects to be recognized. Simpler models often missed these patterns [41, 42]. This study also applied multiple explainability methods (SHAP, Integrated Gradients (IG), and Layer‐wise Relevance Propagation (LRP)) to interpret the model and identify the features most strongly influencing predictions.

Five factors consistently emerged as the most important predictors: freshwater availability, temperature anomaly, hospital bed density, access to electricity, and urban population. Sensitivity analysis confirmed that changes in these predictors strongly affected predicted dengue incidence. These results highlight associations rather than direct cause‐effect relationships, and they should not be interpreted as proof that changing one factor will directly reduce dengue.

Environmental factors, particularly freshwater availability and temperature, had the largest contributions at the global level. They likely reflect complex pathways, such as mosquito breeding conditions and virus replication rates [43, 44]. Health system and infrastructure variables also played a major role. Access to electricity, hospital bed density, community health services, and overall health coverage were all linked to dengue outcomes. When these services are limited, it becomes harder to control mosquitoes, raise public awareness, and provide timely treatment, which in turn worsens the outbreak impact [45, 46].

Sex‐specific patterns were apparent. Male dengue incidence was more influenced by environmental and infrastructure factors, while female incidence was more affected by healthcare access. These differences may relate to exposure patterns, occupational roles, or healthcare‐seeking behaviors [40, 47, 48].

Clear contrasts were observed between high‐ and low‐incidence countries. In high‐incidence settings, particularly in tropical and densely populated regions, dengue predictions were strongly driven by environmental and infrastructural factors such as freshwater availability, temperature anomalies, and urbanization, reflecting favorable conditions for mosquito proliferation and sustained transmission. In contrast, low‐incidence countries showed weaker environmental signals, with feature contributions more strongly associated with healthcare capacity, electricity access, and surveillance‐related indicators. This pattern suggests that in low‐incidence settings, reported dengue burden may be shaped more by detection capacity and health system readiness than by climatic suitability alone, whereas in high‐incidence countries, transmission dynamics are more tightly coupled with environmental and demographic pressures.

Overall, the explainable AI framework provided transparent insights into model behavior. The agreement between SHAP, IG, and LRP strengthens confidence in the identified predictors. This framework can guide surveillance and risk assessment, highlighting which factors and regions require closer monitoring, without implying direct causation [49, 50].

### 4.1 Limitations

First, the analysis relies on data from 2000 to 2021, creating a temporal gap relative to more recent dengue outbreaks, including those reported in 2024. While the 2019–2021 period was used as a test set to evaluate near‐future trends, actual dengue dynamics may shift due to emerging environmental, social, or policy changes, potentially affecting the generalizability of predictions. Second, although the model incorporates 20 climatic, environmental, health system, and socio‐economic variables, other important factors—such as vector control interventions, vaccination coverage, and local mosquito species distributions—were not included due to a lack of consistent global data. Third, the ConvLSTM model effectively captures spatio‐temporal dependencies but does not explicitly model causal relationships; thus, associations identified through SHAP, IG, and LRP should not be interpreted as direct causation. Fourth, missing data were imputed using KNN, which may introduce bias, especially for countries or years with substantial missingness. Although sensitivity analyses (±50% perturbation) were conducted, alternative imputation strategies could yield slightly different predictions. Fifth, while explainable AI methods provide global and sex‐specific insights, interpretability at finer temporal and country‐specific scales may remain limited, particularly in low‐incidence countries with sparse data or in high‐incidence regions with complex transmission patterns. Finally, operational deployment in public health practice would require additional validation, including real‐time outbreak monitoring, local calibration, and careful assessment of prediction uncertainty to ensure reliable and actionable decision‐making.

## 5 Conclusion

This study developed an interpretable deep learning framework for predicting dengue incidence across 118 countries by integrating climatic, environmental, healthcare, and socio‐economic determinants. The ConvLSTM model captured spatio‐temporal patterns and highlighted sex‐specific variations in risk. Globally, five factors—freshwater availability, temperature anomaly, hospital bed density, electricity access, and urban population—emerged as consistently influential, representing predictive associations rather than causal links. By combining predictive accuracy with explainable AI (SHAP, IG, LRP), this framework offers transparent insights to support surveillance, resource allocation, and early warning systems.

Future studies should integrate real‐time epidemiological and vector surveillance data, apply causal inference methods, and explore localized calibration and scenario‐based simulations to assess intervention impacts. Emphasis on equity‐focused predictions will help identify and prioritize vulnerable populations in dengue prevention and control. Furthermore, extending the model to outbreak detection and error analysis could allow evaluation using precision‐recall curves, calibration plots, and false‐alarm versus detection trade‐offs, thereby enhancing its operational utility for public health decision‐making.

## Author Contributions

**Md. Abu Bokkor Shiddik:** conceptualization, investigation, funding acquisition, writing – original draft, methodology, validation, visualization, writing – review and editing, software, formal analysis, project administration, data curation, resources, supervision.

## Funding

The author has nothing to report.

## Conflicts of Interest

The author declares no conflicts of interest.

## Transparency Statement

The lead author, Md. Abu Bokkor Shiddik, affirms that this manuscript is an honest, accurate, and transparent account of the study being reported; that no important aspects of the study have been omitted; and that any discrepancies from the study as planned (and, if relevant, registered) have been explained.

## Supporting information

RevisedSupplement_1.

## Data Availability

All necessary data and source codes are available at, https://github.com/abubokkorshiddik/Research-and-Publication/wiki/Global-Dengue-2000%E2%80%9021.
