# Supplementary material for: Global Prediction of Dengue Incidence Using an Explainable Artificial Intelligence‐Driven ConvLSTM Integrating Environmental, Health, and Socio‐Economic Determinants
Source: Health Sci Rep. 2026 Apr 6;9(4):e72280. doi: 10.1002/hsr2.72280 (PMC13053675; doi:10.1002/hsr2.72280)
Supplement: Supplementary file 1 — RevisedSupplement_1. [file HSR2-9-e72280-s001.docx]

**Global prediction of dengue incidence using an explainable AI–driven ConvLSTM integrating environmental, health, and socio-economic determinants**

**Guide to Supplementary Information**

The Supplementary Information provides additional methodological details, descriptive statistics, model configurations, and extended explainability analyses supporting the main findings.

Tables S1–S2 document variable selection, multicollinearity assessment (VIF), and hyperparameter configurations, including ConvLSTM grid search. Tables S3–S9 summarize annual and country-level dengue incidence (2000–2021), stratified by sex.

Table S10 compares predictive performance across deep learning models. Figures S1–S2 and Table S11 present ConvLSTM hidden states, activations, and attention patterns across countries and incidence levels.

Tables S12–S18 report global and country-level SHAP feature contributions and correlations. Tables S19–S21 present sensitivity analyses under predictor perturbations. Tables S22–S23 provide complementary explainability results using Integrated Gradients and Layer-wise Relevance Propagation. Table S24 reports projected global dengue incidence for 2022–2032.

**Supplementary information**

**Rationale for variable selection**

The selection of variables in this study was guided by the One Health framework, recognizing the interconnectedness of environmental, climatic, socio-demographic, and health system determinants in shaping dengue transmission dynamics. Climatic and environmental variables (x₁–x₆) such as temperature anomaly, precipitation, humidity, and land use were included due to their well-established influence on mosquito ecology, vector survival, and virus replication rates. Health system and health risk indicators (x₇–x₁₂), including hospital bed density, physician density, health expenditure, and UHC coverage, capture the capacity of national health systems to prevent, detect, and manage dengue outbreaks. Socioeconomic and demographic variables (x₁₃–x₂₀), such as population size, GDP growth, education, urbanization, and freshwater use, were selected to represent human behavior, mobility, and infrastructure factors that facilitate or mitigate disease transmission. Data were obtained from authoritative global repositories including the World Bank, World Health Organization (WHO), United Nations (UN), and Our World in Data, ensuring accuracy, comparability, and temporal consistency across 118 countries. Together, these multidimensional predictors provide a comprehensive representation of the environmental and socio-structural determinants essential for modeling global dengue dynamics.

**Note on 2019–2021 testing period:** Although global case data extend to 2024, this study uses 2019–2021 as the test period to evaluate model generalizability. This period includes recent outbreaks and extreme weather events, making it a relevant proxy for near-current patterns while maintaining consistency with model training.

**Table S1.** Variance inflation factor (VIF) for Dengue predictor variables.

| **Variable** | **VIF** |
| --- | --- |
| x1 | 2.859575 |
| x2 | 1.918281 |
| x3 | 1.165375 |
| x4 | 1.65516 |
| x5 | 1.186052 |
| x6 | 1.940396 |
| x7 | 1.159996 |
| x8 | 1.163625 |
| x9 | 1.717268 |
| x10 | 1.287778 |
| x11 | 1.369023 |
| x12 | 1.293608 |
| x13 | 1.561221 |
| x14 | 1.175926 |
| x15 | 1.34314 |
| x16 | 1.5374 |
| x17 | 3.282844 |
| x18 | 1.465893 |
| x19 | 1.516649 |
| x20 | 2.237362 |

*x1: Relative Humidity; x2: Days with Precipitation over 20mm; x3: Temperature anomaly; x4: Average annual surface temperature; x5: Agricultural land (% of land area); x6: Air pollution; x7: Hospital bed density (per 10,000 population); x8: Density of physicians (per 10,000 population); x9: Domestic general government health expenditure (%); x10: Life expectancy at birth (years); x11: Mortality rate under 5 per 1000 live births; x12: UHC Service Coverage Index (SDG 3.8.1); x13: Population, total; x14: GDP growth (annual %); x15: Population growth (annual %); x16: Access to electricity; x17: Average number of years adults aged 25+ spent in formal education; x18: Crude rate of net migration; x19: Urban population (% of total population); x20: Annual freshwater withdrawals, total (billion cubic meters).*

**Table S2.** Hyperparameter configurations for deep learning models predicting dengue incidence (2000–2018 training, 2019–2021 test) and ConvLSTM gridsearch.

A.Hyperparameter configurations for deep learning models predicting dengue incidence (2000–2018 training, 2019–2021 test)

| **Model** | **Architecture / Layers** | **Hidden Units / Filters** | **Kernel Size** | **Dropout** | **Learning Rate** | **Batch Size** | **Epochs** | **Optimizer** |
| --- | --- | --- | --- | --- | --- | --- | --- | --- |
| ConvLSTM | 2 ConvLSTM layers + FC | 32, 32; FC 64 | 3×3 | 0.2 | 0.001 | 16 | Early stopping 100 max | Adam |
| ANN | 3 hidden layers + FC | 128, 64, 32 | – | 0.2 | 0.001 | 16 | Early stopping 100 max | Adam |
| FNN | 3 hidden layers + FC | 128, 64, 32 | – | 0.2 | 0.001 | 16 | Early stopping 100 max | Adam |
| STCNN | 3 temporal conv + 2 spatial conv + FC | 64, 32, 16 (temporal); 32, 16 (spatial); FC 32 | 1D temporal & 2D spatial | 0.2 | 0.001 | 16 | Early stopping 100 max | Adam |
| STGNN | Temporal graph conv + FC | 32 | – | 0.2 | 0.001 | 16 | Early stopping 100 max | Adam |

B.Convlstm gridsearch

| **hidden_channels** | **dropout** | **learning_rate** | **mean_rmse** | **std_rmse** |
| --- | --- | --- | --- | --- |
| 16 | 0.1 | 0.0005 | 9.340947151 | 0.078546688 |
| 16 | 0.1 | 0.001 | 9.232698441 | 0.148024857 |
| 16 | 0.1 | 0.005 | 7.16149044 | 0.194834024 |
| 16 | 0.2 | 0.0005 | 9.342176437 | 0.070688665 |
| 16 | 0.2 | 0.001 | 9.182034492 | 0.083954737 |
| 16 | 0.2 | 0.005 | 7.403457642 | 0.427845597 |
| 16 | 0.3 | 0.0005 | 9.32171917 | 0.107489556 |
| 16 | 0.3 | 0.001 | 9.187726974 | 0.158015624 |
| 16 | 0.3 | 0.005 | 7.168938637 | 0.15288569 |
| 32 | 0.1 | 0.0005 | 9.331064224 | 0.06899821 |
| 32 | 0.1 | 0.001 | 9.217630386 | 0.086885825 |
| 32 | 0.1 | 0.005 | 6.809535027 | 0.435199767 |
| 32 | 0.2 | 0.0005 | 9.34158802 | 0.062356565 |
| 32 | 0.2 | 0.001 | 9.18629837 | 0.145165548 |
| 32 | 0.2 | 0.005 | 6.916140079 | 0.371996969 |
| 32 | 0.3 | 0.0005 | 9.370950699 | 0.127380177 |
| 32 | 0.3 | 0.001 | 9.208974838 | 0.159949273 |
| 32 | 0.3 | 0.005 | 6.722644806 | 0.387565672 |
| 64 | 0.1 | 0.0005 | 9.364462852 | 0.087331876 |
| 64 | 0.1 | 0.001 | 9.23866272 | 0.153160244 |
| 64 | 0.1 | 0.005 | 5.600932121 | 0.580483794 |
| 64 | 0.2 | 0.0005 | 9.325452805 | 0.167706236 |
| 64 | 0.2 | 0.001 | 9.233751297 | 0.091053471 |
| 64 | 0.2 | 0.005 | 5.975104332 | 0.41342175 |
| 64 | 0.3 | 0.0005 | 9.331995964 | 0.103823312 |
| 64 | 0.3 | 0.001 | 9.272630692 | 0.078667819 |
| 64 | 0.3 | 0.005 | 5.322936535 | 0.447945565 |

**Table S3.** Annual global dengue incidence (2000–2021) and affected countries.

|  | **Both** | **Female** | **Male** |  |
| --- | --- | --- | --- | --- |
| **Year** | **Total Cases (in million)** | **Total Cases (in million)** | **Total Cases (in million)** | **Affected countries** |
| 2000 | 20.77951 | 11.04304 | 9.736468 | 118 |
| 2001 | 21.30418 | 11.32034 | 9.983849 | 118 |
| 2002 | 21.91556 | 11.64332 | 10.27224 | 118 |
| 2003 | 22.57024 | 11.99126 | 10.57898 | 118 |
| 2004 | 23.24501 | 12.35194 | 10.89307 | 118 |
| 2005 | 23.89822 | 12.7047 | 11.19351 | 118 |
| 2006 | 24.77869 | 13.18726 | 11.59143 | 118 |
| 2007 | 26.04149 | 13.88462 | 12.15686 | 118 |
| 2008 | 27.49762 | 14.69275 | 12.80487 | 118 |
| 2009 | 28.939 | 15.49892 | 13.44009 | 118 |
| 2010 | 30.15332 | 16.18271 | 13.97061 | 118 |
| 2011 | 31.61741 | 17.00113 | 14.61628 | 118 |
| 2012 | 33.68016 | 18.14208 | 15.53808 | 118 |
| 2013 | 35.90221 | 19.3661 | 16.5361 | 118 |
| 2014 | 61.28861 | 33.14261 | 28.146 | 118 |
| 2015 | 65.88089 | 35.63094 | 30.24995 | 118 |
| 2016 | 64.76509 | 35.03218 | 29.73291 | 118 |
| 2017 | 68.98936 | 37.14161 | 31.84775 | 118 |
| 2018 | 62.46964 | 33.68763 | 28.782 | 118 |
| 2019 | 64.32425 | 34.76174 | 29.56251 | 118 |
| 2020 | 79.51636 | 43.28142 | 36.23495 | 118 |
| 2021 | 85.59419 | 46.32403 | 39.27016 | 118 |

*Both: Total dengue incidence combining males and females (in millions); Female: Total dengue incidence among females (in millions); Male: Total dengue incidence among males (in millions); Affected countries: Number of countries reporting dengue cases each year.*

**Table S4.** Yearly summary of dengue incidence (male) across all affected countries, 2000–2021.

| **Year** | **Min** | **Q1** | **Median** | **Q3** | **Mean_SD** |
| --- | --- | --- | --- | --- | --- |
| 2000 | 0.247 | 439.56 | 1892.174 | 14082.177 | 82512.441 ± 472021.88 |
| 2001 | 0.237 | 419.507 | 1808.805 | 14131.567 | 84608.894 ± 483447.743 |
| 2002 | 0.226 | 321.257 | 1682.447 | 13213.786 | 87052.872 ± 494751.766 |
| 2003 | 0.216 | 256.475 | 1814.786 | 12428.723 | 89652.409 ± 506342.314 |
| 2004 | 0.206 | 258.902 | 1943.955 | 12665.358 | 92314.186 ± 518524.977 |
| 2005 | 0.2 | 268.383 | 2053.017 | 13375.491 | 94860.281 ± 531549.869 |
| 2006 | 0.195 | 361.782 | 2295.737 | 16463.975 | 98232.491 ± 547267.62 |
| 2007 | 0.192 | 459.387 | 2360.194 | 18356.872 | 103024.241 ± 566697.281 |
| 2008 | 0.19 | 500.16 | 2404.503 | 17797.377 | 108515.837 ± 588687.062 |
| 2009 | 0.191 | 546.426 | 2734.377 | 17790.36 | 113899.034 ± 611768.875 |
| 2010 | 0.194 | 520.294 | 2872.238 | 17969.572 | 118394.997 ± 634027.726 |
| 2011 | 0.207 | 565.853 | 2914.542 | 18747.001 | 123866.756 ± 665022.351 |
| 2012 | 0.233 | 526.153 | 2792.333 | 20441.976 | 131678.619 ± 710862.47 |
| 2013 | 0.262 | 521.422 | 2796.08 | 22367.965 | 140136.458 ± 762865.05 |
| 2014 | 0.29 | 394.9 | 2503.357 | 29138.454 | 238525.448 ± 1290382.91 |
| 2015 | 0.314 | 419.874 | 2366.047 | 31512.45 | 256355.485 ± 1385995.16 |
| 2016 | 0.324 | 394.434 | 2585.765 | 31611.299 | 251973.799 ± 1387501.622 |
| 2017 | 0.329 | 491.061 | 3337.68 | 35551.855 | 269896.151 ± 1381342.89 |
| 2018 | 0.345 | 479.688 | 3616.794 | 34544.097 | 243915.29 ± 1357583.162 |
| 2019 | 0.364 | 619.812 | 4010.323 | 31331.427 | 250529.775 ± 1405542.649 |
| 2020 | 0.41 | 662.342 | 5675.127 | 38588.084 | 307075.82 ± 1583646.357 |
| 2021 | 0.416 | 797.668 | 4826.648 | 40735.105 | 332797.958 ± 1952043.999 |

Note: *Values are dengue incidence per country per year. Min, Q1, Median, and Q3 represent the minimum, first quartile, median, and third quartile across all countries with non-zero incidence from 2000–2021. Mean ± SD represents the mean incidence ± standard deviation across all countries. Countries with any zero incidence over the period were excluded.*

**Table S5.** Country-wise summary of average dengue incidence (male) from 2000–2021

| **Country** | **Min** | **Q1** | **Median** | **Q3** | **Mean_SD** |
| --- | --- | --- | --- | --- | --- |
| Afghanistan | 64.711 | 83.785 | 100.594 | 137.634 | 112.627 ± 36.179 |
| Angola | 1220.818 | 2274.41 | 5228.429 | 7068.75 | 5099.69 ± 2927.948 |
| Antigua and Barbuda | 12.516 | 12.868 | 13.925 | 33.973 | 21.805 ± 12.049 |
| Argentina | 16675.551 | 18077.553 | 20447.716 | 36605.822 | 32406.171 ± 26212.395 |
| Australia | 2886.912 | 3342.858 | 5181.676 | 7331.436 | 7651.095 ± 11144.821 |
| Bahamas | 1798.849 | 2191.6 | 2365.33 | 2617.944 | 2524.579 ± 720.22 |
| Bangladesh | 96856.127 | 109183.094 | 141186.778 | 317470.75 | 197881.782 ± 106797.746 |
| Barbados | 11727.746 | 13718.07 | 14763.883 | 28347.705 | 21467.094 ± 12301.111 |
| Belize | 49.988 | 96.159 | 320.314 | 364.748 | 305.283 ± 225.382 |
| Benin | 4612.284 | 6048.131 | 6563.313 | 9479.242 | 7723.756 ± 2511.492 |
| Bhutan | 50.18 | 87.194 | 274.332 | 490.056 | 394.774 ± 372.86 |
| Bolivia (Plurinational State of) | 6594.075 | 9268.277 | 14108.516 | 36850.307 | 22784.396 ± 17143.518 |
| Brazil | 3279539.473 | 3384742.789 | 4151852.441 | 9644053.541 | 6191453.59 ± 3399890.283 |
| Brunei Darussalam | 44.881 | 65.122 | 167.051 | 191.616 | 149.342 ± 77.298 |
| Burkina Faso | 1489.835 | 2145.974 | 3264.775 | 4378.294 | 3435.026 ± 1487.285 |
| Burundi | 158.824 | 214.482 | 307.004 | 389.679 | 325.874 ± 148.583 |
| Cabo Verde | 8293.083 | 9770.866 | 10273.084 | 153036.63 | 171033.973 ± 343174.28 |
| Cambodia | 6110.507 | 9159.709 | 10822.365 | 11315.33 | 10416.361 ± 2276.031 |
| Cameroon | 6520.566 | 7208.014 | 11734.834 | 13667.568 | 11220.819 ± 3769.962 |
| Central African Republic | 1198.699 | 1593.054 | 1878.808 | 3085.868 | 4109.581 ± 7353.175 |
| Chad | 455.866 | 590.215 | 674.405 | 841.866 | 705.895 ± 172.443 |
| China | 3682.342 | 4071.975 | 4975.391 | 16988.831 | 17490.041 ± 31255.881 |
| Colombia | 115768.263 | 140770.297 | 256491.233 | 413993.086 | 294452.775 ± 169543.837 |
| Comoros | 15979.122 | 17237.165 | 18830.601 | 19828.932 | 18512.529 ± 1478.544 |
| Congo | 2740.04 | 3438.622 | 3746.212 | 4334.536 | 3794.267 ± 593.32 |
| Cook Islands | 0.711 | 0.913 | 1.277 | 3.151 | 1.947 ± 1.21 |
| Costa Rica | 23462.926 | 57564.494 | 62372.597 | 70364.737 | 61624.977 ± 18184.79 |
| Cuba | 17584.461 | 17820.165 | 18423.403 | 37858.43 | 32552.496 ± 25110.114 |
| Democratic Republic of the Congo | 35203.804 | 137363.24 | 160851.686 | 181025.104 | 180924.717 ± 87923.055 |
| Djibouti | 762.926 | 949.276 | 1309.491 | 2042.35 | 1503.641 ± 626.8 |
| Dominica | 41.41 | 74.563 | 143.62 | 397.126 | 262.135 ± 284.821 |
| Dominican Republic | 11295.883 | 14108.047 | 25342.463 | 28707.977 | 23642.931 ± 8614.38 |
| Ecuador | 14545.965 | 19232.541 | 24143.926 | 30829.934 | 25827.619 ± 8855.639 |
| Egypt | 3427.811 | 4785.886 | 5811.516 | 6585.311 | 6069.248 ± 2694.411 |
| El Salvador | 54449.364 | 99469.998 | 122744.414 | 135794.719 | 167747.47 ± 188349.4 |
| Equatorial Guinea | 9.822 | 56.739 | 143.901 | 175.775 | 164.044 ± 140.055 |
| Eritrea | 184.678 | 200.741 | 215.085 | 349.926 | 291.817 ± 158.513 |
| Ethiopia | 1353.824 | 1483.827 | 1701.5 | 2283.357 | 1877.912 ± 484.333 |
| Fiji | 3155.601 | 3413.583 | 3746.718 | 36300.746 | 66308.406 ± 214432.925 |
| Gabon | 965.192 | 1226.02 | 1277.5 | 1366.103 | 1299.819 ± 160.868 |
| Gambia | 968.404 | 1138.628 | 1178.916 | 1423.198 | 1347.023 ± 358.334 |
| Ghana | 15942.318 | 18773.808 | 25899.789 | 31863.898 | 28062.395 ± 11970.972 |
| Grenada | 83.988 | 98.305 | 169.198 | 283.232 | 242.473 ± 190.286 |
| Guatemala | 4455.757 | 5073.476 | 8634.13 | 9824.096 | 7981.989 ± 2467.882 |
| Guinea | 938.096 | 1182.383 | 1682.699 | 2430.039 | 1867.264 ± 755.254 |
| Guinea-Bissau | 766.863 | 922.326 | 1054.258 | 1169.256 | 1091.704 ± 246.426 |
| Guyana | 272.703 | 1654.758 | 15417.907 | 35631.439 | 19584.418 ± 16736.45 |
| Haiti | 8265.543 | 9031.279 | 11464.701 | 14592.579 | 12495.429 ± 4015.601 |
| Honduras | 8867.301 | 13743.696 | 21071.753 | 24814.387 | 22400.047 ± 10472.359 |
| India | 3943785.219 | 4729598.278 | 5696332.423 | 10393938.67 | 7533494.374 ± 3730612.662 |
| Indonesia | 583679.721 | 648408.758 | 687682.304 | 1080785.198 | 849976.501 ± 289359.715 |
| Jamaica | 1615.518 | 1872.35 | 6694.931 | 8404.395 | 6286.47 ± 4157.119 |
| Jordan | 50.42 | 117.003 | 368.503 | 830.138 | 515.463 ± 496.672 |
| Kenya | 16131.602 | 17557.111 | 21981.23 | 31730.265 | 24811.032 ± 8508.602 |
| Kiribati | 1533.429 | 1617.927 | 1652.207 | 2010.266 | 1788.429 ± 257.64 |
| Kuwait | 163.98 | 304.797 | 467.083 | 1421.092 | 893.072 ± 758.643 |
| Lao People's Democratic Republic | 1778.613 | 2117.317 | 2756.098 | 5789.685 | 4286.92 ± 2975.112 |
| Lebanon | 132.576 | 233.264 | 430.749 | 1367.41 | 813.24 ± 805.631 |
| Liberia | 1528.617 | 2215.503 | 2344.389 | 6090.675 | 3798.416 ± 2272.455 |
| Madagascar | 781.424 | 1029.097 | 1328.784 | 1750.888 | 1376.613 ± 395.94 |
| Malawi | 621.102 | 718.641 | 804.488 | 967.533 | 943.929 ± 398.384 |
| Malaysia | 47702.488 | 76896.177 | 110633.746 | 269626.966 | 169415.82 ± 113565.455 |
| Maldives | 775.204 | 2686.953 | 3543.586 | 4683.378 | 3514.352 ± 1508.191 |
| Mali | 901.76 | 1240.572 | 1566.817 | 1865.781 | 1577.027 ± 436.312 |
| Marshall Islands | 833.539 | 1110.519 | 1728.492 | 1951.709 | 1580.658 ± 463.423 |
| Mauritania | 149.509 | 173.609 | 226.645 | 246.009 | 227.274 ± 58.729 |
| Mauritius | 2800.732 | 3899.196 | 4586.069 | 49032.541 | 133237.677 ± 473577.843 |
| Mexico | 168619.241 | 223707.152 | 349537.403 | 776985.788 | 566922.268 ± 427230.514 |
| Micronesia (Federated States of) | 119.195 | 125.366 | 133.534 | 197.039 | 162.92 ± 56.156 |
| Mozambique | 5183.949 | 5470.122 | 5959.627 | 6780.902 | 6180.228 ± 801.753 |
| Myanmar | 13675.924 | 18575.578 | 20312.074 | 30052.801 | 27259.703 ± 13758.008 |
| Nauru | 1.711 | 2.369 | 6.663 | 31.544 | 22.729 ± 31.023 |
| Nepal | 37936.813 | 42317.017 | 52624.918 | 99888.405 | 126537.264 ± 158813.242 |
| Nicaragua | 7561.796 | 8930.474 | 12663.276 | 20419.279 | 14748.85 ± 6412.047 |
| Niger | 545.382 | 579.156 | 850.314 | 947.337 | 847.089 ± 272.192 |
| Nigeria | 126699.479 | 175815.955 | 202583.111 | 247752.479 | 216965.624 ± 65772.954 |
| Niue | 0.19 | 0.201 | 0.235 | 0.321 | 0.263 ± 0.074 |
| Oman | 62.831 | 98.632 | 118.057 | 146.164 | 348.149 ± 804.07 |
| Pakistan | 244479.502 | 286197.274 | 332050.357 | 690783.912 | 455834.473 ± 218329.317 |
| Palau | 204.761 | 228.91 | 417.329 | 1778.625 | 2676.072 ± 6186.513 |
| Panama | 4130.275 | 6864.381 | 8815.525 | 9776.525 | 8948.373 ± 3241.565 |
| Papua New Guinea | 74.655 | 88.473 | 104.418 | 240.385 | 221.571 ± 218.68 |
| Paraguay | 18093.166 | 21176.376 | 27655.034 | 45933.433 | 34489.443 ± 16652.155 |
| Peru | 22693.31 | 31956.498 | 48612.523 | 57720.88 | 49690.163 ± 21534.156 |
| Philippines | 34831.039 | 58826.36 | 117702.671 | 243072.13 | 180694.106 ± 165403.985 |
| Rwanda | 265.733 | 293.766 | 527.066 | 657.369 | 731.388 ± 708.878 |
| Saint Kitts and Nevis | 6.683 | 8.019 | 18.907 | 21.393 | 15.881 ± 6.565 |
| Saint Lucia | 85.575 | 88.673 | 113.454 | 162.252 | 132.306 ± 52.006 |
| Saint Vincent and the Grenadines | 79.826 | 91.066 | 182.987 | 253.18 | 180.374 ± 84.512 |
| Samoa | 189.515 | 205.749 | 222.756 | 315.056 | 300.002 ± 156.279 |
| Sao Tome and Principe | 365.439 | 492.407 | 547.97 | 568.326 | 516.785 ± 70.905 |
| Saudi Arabia | 31.901 | 42.475 | 88.267 | 199.454 | 694.177 ± 2295.258 |
| Senegal | 753.984 | 947.289 | 1278.364 | 1492.733 | 1269.366 ± 354.764 |
| Seychelles | 2165.776 | 3725.462 | 4133.058 | 4648.251 | 4515.96 ± 1458.913 |
| Sierra Leone | 4265.493 | 6002.084 | 6642.958 | 7963.587 | 6990.188 ± 1729.128 |
| Singapore | 53574.343 | 143008.563 | 183607.101 | 242404.643 | 348524.072 ± 648651.576 |
| Solomon Islands | 429.523 | 475.491 | 536.957 | 828.218 | 701.562 ± 326.657 |
| Somalia | 745.628 | 835.064 | 914.701 | 1054.018 | 957.09 ± 183.517 |
| South Sudan | 6.541 | 10.663 | 13.392 | 46.58 | 90.217 ± 178.673 |
| Sri Lanka | 23537.884 | 30510.971 | 64559.546 | 148556.21 | 98005.447 ± 87859.575 |
| Sudan | 666.022 | 834.191 | 1033.501 | 1345.171 | 1095.017 ± 303.186 |
| Suriname | 394.804 | 703.56 | 780.508 | 1310.869 | 951.185 ± 452.166 |
| Syrian Arab Republic | 55.95 | 96.809 | 195.438 | 891.538 | 483.862 ± 514.5 |
| Thailand | 76011.071 | 90032.97 | 171209.009 | 547216.963 | 363723.756 ± 385336.362 |
| Timor-Leste | 39.441 | 87.53 | 91.406 | 578.992 | 287.254 ± 286.521 |
| Togo | 3891.035 | 4560.936 | 4914.191 | 6930.928 | 5813.538 ± 1813.454 |
| Tonga | 1459.982 | 12224.22 | 13521.782 | 15569.883 | 29580.12 ± 55195.374 |
| Trinidad and Tobago | 10731.372 | 12603.184 | 13687.127 | 15472.827 | 16284.056 ± 10405.777 |
| Tuvalu | 2.401 | 3.873 | 7.564 | 18.02 | 19.484 ± 27.145 |
| Uganda | 2000.003 | 2368.457 | 2638.836 | 3097.263 | 2981.421 ± 1085.43 |
| United Republic of Tanzania | 407.34 | 456.504 | 807.872 | 1575.053 | 1613.714 ± 2219.955 |
| United States of America | 14.084 | 17.277 | 69.423 | 107.922 | 163.722 ± 296.954 |
| Vanuatu | 99.687 | 110.736 | 170.767 | 365.284 | 235.803 ± 141.021 |
| Venezuela (Bolivarian Republic of) | 50630.155 | 56485.447 | 66675.572 | 104612.2 | 85310.023 ± 38672.491 |
| Viet Nam | 226672.584 | 268444.282 | 281133.666 | 321618.628 | 317766.156 ± 90588.211 |
| Yemen | 1298.973 | 1879.433 | 2021.045 | 2467.924 | 2306.147 ± 802.653 |
| Zambia | 724.815 | 792.679 | 996.34 | 1204.346 | 1062.669 ± 328.608 |
| Zimbabwe | 258.353 | 278.851 | 380.076 | 419.785 | 359.768 ± 68.655 |

*Summary statistics include minimum (Min), first quartile (Q1), median, third quartile (Q3), and mean ± standard deviation (Mean ± SD) of dengue incidence per country. Countries with any zero incidence over 2000–2021 were excluded.*

**Table S6.** Country-wise summary of average dengue incidence (female) from 2000–2021

| **Country** | **Min** | **Q1** | **Median** | **Q3** | **Mean_SD** |
| --- | --- | --- | --- | --- | --- |
| Afghanistan | 71.209 | 93.427 | 110.925 | 154.366 | 125.287 ± 40.875 |
| Angola | 1447.196 | 2726.538 | 6358.591 | 8691.525 | 6230.142 ± 3630.842 |
| Antigua and Barbuda | 15.86 | 17.144 | 18.87 | 43.952 | 28.142 ± 14.892 |
| Argentina | 20473.875 | 21897.269 | 25453.335 | 49124.5 | 41478.564 ± 34248.991 |
| Australia | 3450.504 | 4064.885 | 6703.875 | 9104.277 | 9737.792 ± 14574.821 |
| Bahamas | 2209.311 | 2652.961 | 2880.001 | 3204.33 | 3084.06 ± 892.087 |
| Bangladesh | 104763.81 | 122397.341 | 168783.338 | 390523.213 | 238385.132 ± 138110.24 |
| Barbados | 14908.709 | 17831.08 | 18781.147 | 36737.68 | 27593.372 ± 15710.821 |
| Belize | 57.515 | 108.858 | 383.917 | 431.921 | 360.123 ± 266.387 |
| Benin | 5526.147 | 7242.538 | 7821.528 | 11256.218 | 9195.734 ± 2950.072 |
| Bhutan | 51.563 | 90.294 | 289.853 | 507.164 | 410.637 ± 386.524 |
| Bolivia (Plurinational State of) | 7585.919 | 10887.512 | 16520.968 | 44645.144 | 27127.922 ± 20635.146 |
| Brazil | 4010080.379 | 4104898.757 | 5201650.604 | 11979868.65 | 7687881.409 ± 4273944.565 |
| Brunei Darussalam | 49.807 | 74.04 | 183.718 | 206.627 | 160.918 ± 79.855 |
| Burkina Faso | 1808.053 | 2601.212 | 4040.63 | 5408.501 | 4236.79 ± 1868.548 |
| Burundi | 191.631 | 250.962 | 374.799 | 446.769 | 414.732 ± 243.479 |
| Cabo Verde | 10057.148 | 11623.414 | 11967.286 | 173480.127 | 195963.927 ± 393516.517 |
| Cambodia | 7772.08 | 11718.289 | 13738.943 | 14409.973 | 13172.741 ± 2775.401 |
| Cameroon | 7625.635 | 8374.167 | 13556.967 | 15802.63 | 12990.183 ± 4325.978 |
| Central African Republic | 1391.129 | 1840.035 | 2178.988 | 3647.354 | 4810.129 ± 8624.734 |
| Chad | 545.439 | 698.82 | 790.588 | 979.699 | 827.736 ± 194.595 |
| China | 4069.886 | 4391.328 | 5226.774 | 17807.298 | 18695.501 ± 32816.559 |
| Colombia | 138621.188 | 178254.821 | 325816.725 | 542094.945 | 372661.601 ± 212827.675 |
| Comoros | 18109.133 | 19586.009 | 21584.578 | 23590.806 | 21387.779 ± 2038.933 |
| Congo | 3279.465 | 4071.36 | 4425.637 | 5065.654 | 4482.126 ± 672.974 |
| Cook Islands | 0.9 | 1.126 | 1.579 | 4.589 | 2.721 ± 1.859 |
| Costa Rica | 27201.411 | 69527.714 | 80055.363 | 87933.738 | 77091.492 ± 23829.08 |
| Cuba | 20360.764 | 20602.934 | 21613.668 | 45667.688 | 38485.367 ± 30109.379 |
| Democratic Republic of the Congo | 39824.607 | 156709.292 | 182442.059 | 204251.609 | 204939.808 ± 98735.478 |
| Djibouti | 758.858 | 933.878 | 1325.439 | 1990.312 | 1480.848 ± 613.198 |
| Dominica | 47.413 | 88.125 | 165.632 | 468.984 | 306.165 ± 331.234 |
| Dominican Republic | 13523.048 | 16835.296 | 30271.056 | 34254.687 | 28134.511 ± 10186.077 |
| Ecuador | 17207.027 | 23149.583 | 29242.106 | 37851.962 | 31549.021 ± 11392.093 |
| Egypt | 3758.584 | 5215.545 | 6241.197 | 7062.344 | 6535.302 ± 2836.586 |
| El Salvador | 69307.437 | 134526.133 | 170830.181 | 187630.176 | 232199.095 ± 265110.494 |
| Equatorial Guinea | 11.539 | 61.846 | 141.541 | 173.396 | 161.985 ± 131.292 |
| Eritrea | 203.984 | 223.231 | 239.286 | 410.513 | 330.614 ± 179.676 |
| Ethiopia | 1512.617 | 1667.298 | 1897.06 | 2559.308 | 2116.291 ± 542.644 |
| Fiji | 3458.669 | 3741.116 | 4123.321 | 42291.678 | 77134.878 ± 250827.382 |
| Gabon | 1185.205 | 1481.602 | 1551.966 | 1642.335 | 1584.201 ± 208.794 |
| Gambia | 1126.038 | 1343.165 | 1390.775 | 1692.038 | 1599.333 ± 442.098 |
| Ghana | 18675.601 | 21762.414 | 30795.915 | 38427.695 | 33514.556 ± 14766.637 |
| Grenada | 101.75 | 120.125 | 202.182 | 338.838 | 287.176 ± 221.234 |
| Guatemala | 5560.872 | 6344.365 | 11069.156 | 12672.782 | 10177.863 ± 3207.025 |
| Guinea | 1144.859 | 1430.213 | 2063.999 | 2976.344 | 2278.782 ± 923.096 |
| Guinea-Bissau | 920.729 | 1108.536 | 1266.975 | 1405.714 | 1309.355 ± 292.787 |
| Guyana | 326.763 | 2056.461 | 17520.346 | 42577.647 | 23142.037 ± 20059.04 |
| Haiti | 10007.796 | 10894.086 | 14194.946 | 17901.813 | 15066.683 ± 4728.673 |
| Honduras | 11055.667 | 16674.78 | 26021.213 | 30593.682 | 27616.969 ± 13055.386 |
| India | 4119950.042 | 5002846.541 | 6108232.622 | 11272119.49 | 8106364.101 ± 4112544.958 |
| Indonesia | 677020.201 | 743154.821 | 787899.994 | 1249422.102 | 979124.277 ± 335242.594 |
| Jamaica | 1916.376 | 2213.158 | 8123.058 | 10310.416 | 7645.327 ± 5105.634 |
| Jordan | 54.4 | 126.146 | 367.387 | 916.369 | 566.674 ± 559.235 |
| Kenya | 17150.566 | 18868.854 | 24180.823 | 35574.516 | 27460.256 ± 10053.803 |
| Kiribati | 1786.698 | 1904.563 | 1995.101 | 2418.702 | 2135.64 ± 318.501 |
| Kuwait | 113.432 | 267.387 | 405.001 | 1264.368 | 689.013 ± 560.919 |
| Lao People's Democratic Republic | 2060.947 | 2488.764 | 3284.673 | 6868.465 | 5072.027 ± 3516.903 |
| Lebanon | 155.236 | 281.053 | 518.454 | 1574.188 | 950.818 ± 940.58 |
| Liberia | 1756.588 | 2501.808 | 2659.997 | 6819.147 | 4274.901 ± 2521.671 |
| Madagascar | 910.517 | 1201.947 | 1552.688 | 2084.299 | 1629.919 ± 472.762 |
| Malawi | 746.697 | 872.183 | 968.788 | 1202.024 | 1168.364 ± 554.102 |
| Malaysia | 52865.067 | 87195.108 | 119826.944 | 297623.625 | 185825.799 ± 122540.493 |
| Maldives | 927.739 | 2084.32 | 2925.933 | 4172.311 | 2997.892 ± 1139.817 |
| Mali | 1046.885 | 1434.701 | 1819.954 | 2172.093 | 1832.502 ± 510.965 |
| Marshall Islands | 985.512 | 1324.689 | 1888.93 | 2155.685 | 1785.769 ± 501.426 |
| Mauritania | 174.367 | 203.452 | 268.897 | 292.961 | 269.544 ± 72.098 |
| Mauritius | 4170.806 | 5981.238 | 6978.246 | 72959.009 | 199911.016 ± 712536.031 |
| Mexico | 198603.535 | 267752.223 | 422066.928 | 981870.027 | 697346.258 ± 529576.07 |
| Micronesia (Federated States of) | 131.652 | 141.156 | 151.611 | 219.436 | 183.169 ± 63.79 |
| Mozambique | 6454.851 | 6827.145 | 7619.213 | 8497.043 | 7726.473 ± 968.759 |
| Myanmar | 17053.336 | 24547.057 | 26752.348 | 39868.833 | 35684.192 ± 18016.572 |
| Nauru | 2.316 | 2.992 | 8.555 | 40.861 | 29.175 ± 39.271 |
| Nepal | 43161.858 | 49278.688 | 63289.882 | 124693.91 | 154928.465 ± 197634.785 |
| Nicaragua | 8906.891 | 10557.568 | 15514.538 | 25315.934 | 18171.276 ± 8375.52 |
| Niger | 625.567 | 665.012 | 982.779 | 1099.165 | 979.619 ± 320.267 |
| Nigeria | 139977.437 | 194550.106 | 235602.71 | 304830.43 | 257939.319 ± 92314.699 |
| Niue | 0.223 | 0.234 | 0.283 | 0.407 | 0.321 ± 0.099 |
| Oman | 50.674 | 93.667 | 105.726 | 123.868 | 271.98 ± 596.749 |
| Pakistan | 258988.302 | 306441.137 | 359023.652 | 754350.594 | 494894.93 ± 241761.673 |
| Palau | 204.952 | 228.13 | 385.524 | 1718.553 | 2541.438 ± 5873.54 |
| Panama | 4775.468 | 8129.691 | 10420.461 | 11769.199 | 10832.794 ± 4341.413 |
| Papua New Guinea | 80.118 | 93.755 | 109.506 | 266.894 | 252.979 ± 266.589 |
| Paraguay | 20280.248 | 24182.806 | 31996.725 | 51876.889 | 39889.529 ± 19989.868 |
| Peru | 26735.492 | 37548.571 | 56860.339 | 68029.958 | 58763.333 ± 26227.348 |
| Philippines | 40083.383 | 71286.948 | 139981.91 | 283207.714 | 215737.861 ± 201521.831 |
| Rwanda | 323.754 | 359.999 | 647.876 | 848.997 | 1003.459 ± 1119.96 |
| Saint Kitts and Nevis | 7.761 | 9.262 | 21.985 | 24.737 | 18.401 ± 7.591 |
| Saint Lucia | 104.894 | 109.025 | 134.283 | 193.919 | 159.928 ± 63.441 |
| Saint Vincent and the Grenadines | 87.622 | 102.531 | 205.066 | 283.402 | 201.719 ± 94.748 |
| Samoa | 200.18 | 217.803 | 237.145 | 376.397 | 334.063 ± 183.152 |
| Sao Tome and Principe | 420.588 | 568.374 | 633.887 | 655.254 | 596.811 ± 82.32 |
| Saudi Arabia | 28.996 | 38.05 | 69.616 | 142.756 | 496.592 ± 1624.094 |
| Senegal | 888.756 | 1102.414 | 1502.324 | 1750.622 | 1493.309 ± 425.004 |
| Seychelles | 2323.051 | 4162.142 | 4554.028 | 4983.01 | 4910.277 ± 1476.561 |
| Sierra Leone | 5240.3 | 7194.243 | 7887.001 | 9380.62 | 8297.442 ± 1949.645 |
| Singapore | 62756.516 | 154878.961 | 201491.916 | 263235.424 | 382176.305 ± 704780.088 |
| Solomon Islands | 456.306 | 509.193 | 577.335 | 860.585 | 749.387 ± 359.304 |
| Somalia | 800.945 | 900.552 | 997.133 | 1145.864 | 1043.774 ± 214.135 |
| South Sudan | 7.408 | 11.322 | 14.832 | 49 | 94.459 ± 184.643 |
| Sri Lanka | 27961.863 | 37394.23 | 84116.816 | 203574.455 | 133533.502 ± 124525.649 |
| Sudan | 745.385 | 921.507 | 1149.704 | 1516.804 | 1227.171 ± 354.864 |
| Suriname | 461.149 | 823.904 | 914.38 | 1539.33 | 1114.559 ± 529.337 |
| Syrian Arab Republic | 73.658 | 112.266 | 206.872 | 988.643 | 554.381 ± 599.696 |
| Thailand | 91342.2 | 113786.392 | 215845.688 | 716081.489 | 488937.506 ± 546761.941 |
| Timor-Leste | 44.272 | 97.47 | 103.716 | 683.557 | 338.149 ± 344.223 |
| Togo | 4643.129 | 5415.908 | 5818.727 | 8186.113 | 6880.884 ± 2123.666 |
| Tonga | 1613.55 | 13764.257 | 15016.673 | 17672.944 | 33675.471 ± 63366.634 |
| Trinidad and Tobago | 12317.963 | 14960.088 | 16211.747 | 18393.808 | 19157.728 ± 11999.029 |
| Tuvalu | 3.305 | 5.039 | 9.252 | 23.505 | 24.493 ± 33.653 |
| Uganda | 2415.044 | 2873.124 | 3118.537 | 3663.238 | 3822.468 ± 1942.6 |
| United Republic of Tanzania | 491.19 | 548.883 | 1009.638 | 1967.759 | 1992.553 ± 2689.895 |
| United States of America | 16.139 | 19.894 | 83.102 | 131.368 | 198.802 ± 362.225 |
| Vanuatu | 108.564 | 121.74 | 196.399 | 461.144 | 282.608 ± 178.925 |
| Venezuela (Bolivarian Republic of) | 62751.708 | 68239.391 | 80439.624 | 126504.62 | 103319.738 ± 46410.625 |
| Viet Nam | 270858.991 | 330513.911 | 342925.1 | 386439.601 | 391530.034 ± 121645.18 |
| Yemen | 1415.124 | 2102.263 | 2390.502 | 2814.194 | 2642.666 ± 953.535 |
| Zambia | 854.248 | 942.23 | 1207.425 | 1413.389 | 1301.798 ± 490.602 |
| Zimbabwe | 323.42 | 351.528 | 466.954 | 530.163 | 449.634 ± 86.426 |

*Summary statistics include minimum (Min), first quartile (Q1), median, third quartile (Q3), and mean ± standard deviation (Mean ± SD) of dengue incidence per country. Countries with any zero incidence over 2000–2021 were excluded.*

**Table S7.** Yearly summary of dengue incidence (female) across all affected countries, 2000–2021.

| **Year** | **Min** | **Q1** | **Median** | **Q3** | **Mean_SD** |
| --- | --- | --- | --- | --- | --- |
| 2000 | 0.294 | 514.862 | 2361.636 | 17278.314 | 93585.126 ± 529221.6 |
| 2001 | 0.283 | 472.073 | 2175.192 | 17469.961 | 95935.047 ± 541421.263 |
| 2002 | 0.268 | 373.882 | 2074.515 | 16666.251 | 98672.182 ± 552863.938 |
| 2003 | 0.253 | 298.895 | 2123.963 | 15692.149 | 101620.833 ± 564483.099 |
| 2004 | 0.24 | 254.085 | 2339.235 | 15296.914 | 104677.428 ± 577101.345 |
| 2005 | 0.232 | 253.843 | 2482.979 | 15870.766 | 107666.988 ± 591385.318 |
| 2006 | 0.228 | 391.252 | 2706.685 | 19546.005 | 111756.401 ± 609755.328 |
| 2007 | 0.225 | 522.228 | 2781.748 | 21246.719 | 117666.311 ± 633413.67 |
| 2008 | 0.223 | 556.374 | 2936.435 | 20629.229 | 124514.855 ± 660940 |
| 2009 | 0.225 | 642.734 | 3340.252 | 20600.909 | 131346.743 ± 690415.381 |
| 2010 | 0.229 | 579.386 | 3534.778 | 20902.218 | 137141.593 ± 719386.135 |
| 2011 | 0.247 | 618.056 | 3559.198 | 21789.46 | 144077.373 ± 759925.162 |
| 2012 | 0.284 | 627.432 | 3409.614 | 24229.797 | 153746.45 ± 819405.032 |
| 2013 | 0.325 | 568.11 | 3309.965 | 26395.088 | 164119.53 ± 886316.971 |
| 2014 | 0.365 | 428.727 | 2668.132 | 35132.723 | 280869.587 ± 1522447.175 |
| 2015 | 0.398 | 487.26 | 2617.224 | 38775.318 | 301957.159 ± 1634471.104 |
| 2016 | 0.41 | 476.507 | 2709.927 | 37515.69 | 296882.921 ± 1632269.691 |
| 2017 | 0.417 | 571.364 | 3329.43 | 42239.769 | 314759.447 ± 1601367.79 |
| 2018 | 0.438 | 554.006 | 3654.261 | 40881.5 | 285488.428 ± 1554609.497 |
| 2019 | 0.464 | 722.481 | 4357.734 | 35971.359 | 294590.977 ± 1598486.999 |
| 2020 | 0.517 | 774.133 | 5924.381 | 44614.38 | 366791.659 ± 1818884.369 |
| 2021 | 0.504 | 944.782 | 4759.824 | 46485.898 | 392576.523 ± 2252283.554 |

Note: *Values are dengue incidence per country per year. Min, Q1, Median, and Q3 represent the minimum, first quartile, median, and third quartile across all countries with non-zero incidence from 2000–2021. Mean ± SD represents the mean incidence ± standard deviation across all countries. Countries with any zero incidence over the period were excluded.*

**Table S8.** Yearly summary of dengue incidence (both sexes) across all affected countries, 2000–2021.

| **Year** | **Min** | **Q1** | **Median** | **Q3** | **Mean ± SD** |
| --- | --- | --- | --- | --- | --- |
| 2000 | 0.541 | 981.816 | 4243.638 | 31908.754 | 176097.567 ± 1000469.211 |
| 2001 | 0.52 | 910.778 | 3954.848 | 31348.414 | 180543.94 ± 1024109.062 |
| 2002 | 0.494 | 709.814 | 3835.794 | 29727.044 | 185725.054 ± 1046890.147 |
| 2003 | 0.468 | 561.791 | 3916.563 | 28074.343 | 191273.242 ± 1070141.832 |
| 2004 | 0.447 | 523.279 | 4283.191 | 27912.275 | 196991.614 ± 1094980.499 |
| 2005 | 0.432 | 542.552 | 4535.995 | 29117.441 | 202527.269 ± 1122310.235 |
| 2006 | 0.423 | 765.882 | 5002.422 | 36072.435 | 209988.892 ± 1156385.15 |
| 2007 | 0.417 | 971.123 | 5141.941 | 39494.603 | 220690.552 ± 1199423.759 |
| 2008 | 0.414 | 1059.067 | 5340.938 | 38426.606 | 233030.692 ± 1248862.822 |
| 2009 | 0.415 | 1193.738 | 6074.63 | 38391.27 | 245245.777 ± 1301325.303 |
| 2010 | 0.423 | 1092.469 | 6407.016 | 38819.306 | 255536.59 ± 1352453.58 |
| 2011 | 0.455 | 1184.513 | 6473.74 | 40536.461 | 267944.129 ± 1423886.454 |
| 2012 | 0.516 | 1153.585 | 6201.948 | 44671.773 | 285425.069 ± 1529121.366 |
| 2013 | 0.587 | 1077.486 | 6057.104 | 48359.384 | 304255.988 ± 1647980.9 |
| 2014 | 0.655 | 843.374 | 4954.97 | 63334.34 | 519395.035 ± 2810907.763 |
| 2015 | 0.712 | 907.134 | 4917.713 | 70077.41 | 558312.644 ± 3018436.459 |
| 2016 | 0.734 | 864.846 | 5153.986 | 68474.733 | 548856.72 ± 3017767.215 |
| 2017 | 0.745 | 1062.425 | 6244.548 | 77791.625 | 584655.597 ± 2980815.876 |
| 2018 | 0.784 | 1033.694 | 6803.158 | 75385.575 | 529403.718 ± 2910551.032 |
| 2019 | 0.829 | 1342.751 | 8087.795 | 67021.048 | 545120.753 ± 3002409.131 |
| 2020 | 0.927 | 1436.475 | 10978.63 | 83072.431 | 673867.48 ± 3399972.324 |
| 2021 | 0.92 | 1749.762 | 9342.737 | 87221.003 | 725374.481 ± 4201987.909 |

Note: Values are dengue incidence per country per year. Min, Q1, Median, and Q3 represent the minimum, first quartile, median, and third quartile across all countries with non-zero incidence from 2000–2021. Mean ± SD represents the mean incidence ± standard deviation across all countries. Countries with any zero incidence over the period were excluded.

**Table S9.** Country-wise summary of average dengue incidence (Both sexes) from 2000–2021

| **Country** | **Min** | **Q1** | **Median** | **Q3** | **Mean_SD** |
| --- | --- | --- | --- | --- | --- |
| Afghanistan | 135.92 | 177.692 | 211.519 | 292 | 237.914 ± 77.043 |
| Angola | 2668.014 | 5000.948 | 11587.02 | 15760.275 | 11329.833 ± 6558.731 |
| Antigua and Barbuda | 28.375 | 29.845 | 32.75 | 77.925 | 49.947 ± 26.936 |
| Argentina | 37149.427 | 39974.823 | 45901.052 | 85730.322 | 73884.734 ± 60452.593 |
| Australia | 6337.416 | 7407.743 | 11885.551 | 16435.713 | 17388.887 ± 25718.99 |
| Bahamas | 4008.16 | 4844.562 | 5245.33 | 5822.274 | 5608.639 ± 1612.256 |
| Bangladesh | 201619.937 | 231580.434 | 309970.116 | 707993.963 | 436266.914 ± 244892.859 |
| Barbados | 26636.455 | 31449.744 | 33362.535 | 65085.385 | 49060.466 ± 28010.613 |
| Belize | 107.503 | 205.017 | 704.231 | 796.568 | 665.406 ± 491.742 |
| Benin | 10138.431 | 13290.67 | 14384.841 | 20735.461 | 16919.49 ± 5461.557 |
| Bhutan | 101.743 | 177.488 | 564.185 | 997.22 | 805.411 ± 759.38 |
| Bolivia (Plurinational State of) | 14179.994 | 20155.789 | 30629.483 | 81495.451 | 49912.318 ± 37776.825 |
| Brazil | 7289619.852 | 7489641.546 | 9353503.045 | 21623922.19 | 13879334.999 ± 7673614.065 |
| Brunei Darussalam | 94.687 | 139.162 | 352.197 | 394.797 | 310.26 ± 157.08 |
| Burkina Faso | 3297.889 | 4747.186 | 7305.404 | 9786.795 | 7671.816 ± 3355.817 |
| Burundi | 350.455 | 465.46 | 682.848 | 836.447 | 740.606 ± 389.392 |
| Cabo Verde | 18350.231 | 21394.28 | 22240.37 | 326516.757 | 366997.901 ± 736689.799 |
| Cambodia | 13882.587 | 20877.998 | 24561.308 | 25725.303 | 23589.101 ± 5048.788 |
| Cameroon | 14146.201 | 15582.181 | 25291.801 | 29470.198 | 24211.002 ± 8095.906 |
| Central African Republic | 2589.827 | 3433.089 | 4057.796 | 6733.222 | 8919.71 ± 15977.88 |
| Chad | 1001.305 | 1289.035 | 1464.993 | 1821.564 | 1533.631 ± 367.033 |
| China | 7752.227 | 8463.303 | 10202.165 | 34796.128 | 36185.541 ± 64064.373 |
| Colombia | 254389.451 | 319025.118 | 582307.958 | 966185.853 | 667114.376 ± 382199.71 |
| Comoros | 34088.255 | 36862.087 | 40358.564 | 43253.301 | 39900.307 ± 3487.675 |
| Congo | 6019.505 | 7509.981 | 8177.079 | 9400.19 | 8276.393 ± 1265.985 |
| Cook Islands | 1.611 | 2.018 | 2.856 | 7.74 | 4.668 ± 3.068 |
| Costa Rica | 50664.336 | 127544.838 | 142647.613 | 158543.784 | 138716.469 ± 41965.837 |
| Cuba | 38032.428 | 38502.406 | 39688.94 | 83526.118 | 71037.864 ± 55217.715 |
| Democratic Republic of the Congo | 75028.411 | 294072.532 | 343293.746 | 385276.713 | 385864.525 ± 186656.456 |
| Djibouti | 1521.784 | 1883.154 | 2634.93 | 4027.239 | 2984.489 ± 1239.597 |
| Dominica | 88.823 | 162.688 | 310.421 | 866.11 | 568.299 ± 616.038 |
| Dominican Republic | 24818.932 | 30943.343 | 55613.519 | 63090.6 | 51777.443 ± 18799.34 |
| Ecuador | 31752.991 | 42355.999 | 53386.033 | 68515.115 | 57376.64 ± 20240.563 |
| Egypt | 7186.396 | 10001.431 | 12031.791 | 13647.655 | 12604.55 ± 5530.738 |
| El Salvador | 123756.8 | 233996.131 | 293574.595 | 323104.771 | 399946.565 ± 453455.991 |
| Equatorial Guinea | 21.361 | 118.585 | 285.442 | 349.171 | 326.03 ± 271.313 |
| Eritrea | 388.662 | 423.972 | 453.351 | 775.176 | 622.432 ± 337.838 |
| Ethiopia | 2866.441 | 3134.23 | 3598.56 | 4842.665 | 3994.203 ± 1026.236 |
| Fiji | 6614.27 | 7154.699 | 7870.039 | 78592.425 | 143443.284 ± 465259.977 |
| Gabon | 2150.397 | 2698.3 | 2821.629 | 3012.55 | 2884.021 ± 369.081 |
| Gambia | 2094.442 | 2483.31 | 2568.948 | 3115.236 | 2946.356 ± 800.418 |
| Ghana | 34617.919 | 40542.065 | 56695.704 | 70291.593 | 61576.95 ± 26737.234 |
| Grenada | 185.739 | 218.43 | 371.139 | 622.07 | 529.649 ± 411.434 |
| Guatemala | 10016.629 | 11417.841 | 19781.624 | 22424.457 | 18159.852 ± 5671.619 |
| Guinea | 2082.956 | 2610.877 | 3746.698 | 5406.383 | 4146.046 ± 1678.317 |
| Guinea-Bissau | 1687.592 | 2030.861 | 2321.232 | 2574.97 | 2401.059 ± 539.209 |
| Guyana | 599.465 | 3711.219 | 32938.253 | 78370.241 | 42726.454 ± 36792.216 |
| Haiti | 18273.339 | 19919.276 | 25790.506 | 32221.959 | 27562.112 ± 8738.02 |
| Honduras | 19922.968 | 30418.476 | 47092.966 | 55351.268 | 50017.015 ± 23527.05 |
| India | 8063735.261 | 9732444.818 | 11804565.04 | 21666058.16 | 15639858.474 ± 7843126.283 |
| Indonesia | 1260699.922 | 1391563.58 | 1475582.297 | 2330207.299 | 1829100.778 ± 624588.478 |
| Jamaica | 3531.894 | 4085.508 | 14817.989 | 18714.811 | 13931.797 ± 9262.561 |
| Jordan | 104.82 | 240.392 | 735.89 | 1746.506 | 1082.137 ± 1055.785 |
| Kenya | 33282.168 | 36425.964 | 46162.053 | 67304.781 | 52271.288 ± 18562.181 |
| Kiribati | 3320.126 | 3522.49 | 3647.169 | 4428.968 | 3924.069 ± 575.577 |
| Kuwait | 277.411 | 579.011 | 887.236 | 2707.106 | 1582.085 ± 1313.093 |
| Lao People's Democratic Republic | 3839.559 | 4606.082 | 6038.626 | 12658.15 | 9358.947 ± 6491.768 |
| Lebanon | 287.812 | 514.318 | 949.763 | 2941.597 | 1764.058 ± 1746.073 |
| Liberia | 3285.205 | 4717.311 | 5004.386 | 12909.822 | 8073.317 ± 4794.098 |
| Madagascar | 1691.941 | 2228.25 | 2881.471 | 3862.458 | 3006.532 ± 867.536 |
| Malawi | 1367.799 | 1586.542 | 1773.276 | 2162.652 | 2112.293 ± 952.225 |
| Malaysia | 100567.555 | 164091.285 | 230460.69 | 567250.591 | 355241.618 ± 236077.629 |
| Maldives | 1702.943 | 4786.942 | 6762.224 | 8605.142 | 6512.244 ± 2407.606 |
| Mali | 1948.645 | 2675.273 | 3386.771 | 4037.874 | 3409.529 ± 947.269 |
| Marshall Islands | 1819.051 | 2435.208 | 3617.422 | 4104.632 | 3366.426 ± 963.511 |
| Mauritania | 323.875 | 377.061 | 495.542 | 538.97 | 496.818 ± 130.819 |
| Mauritius | 6971.539 | 9880.434 | 11564.315 | 121991.55 | 333148.693 ± 1186112.265 |
| Mexico | 367222.776 | 491459.375 | 771604.332 | 1755271.814 | 1264268.526 ± 956664.114 |
| Micronesia (Federated States of) | 250.847 | 266.522 | 284.435 | 416.474 | 346.09 ± 119.915 |
| Mozambique | 11638.8 | 12394.02 | 13514.571 | 15121.092 | 13906.7 ± 1748.291 |
| Myanmar | 30729.26 | 43129.428 | 47055.653 | 69910.051 | 62943.895 ± 31772.709 |
| Nauru | 4.027 | 5.385 | 15.218 | 72.405 | 51.904 ± 70.282 |
| Nepal | 81098.672 | 91595.705 | 115914.8 | 224560.096 | 281465.729 ± 356444.632 |
| Nicaragua | 16468.687 | 19488.042 | 28177.814 | 45735.213 | 32920.126 ± 14785.041 |
| Niger | 1170.949 | 1244.167 | 1833.094 | 2046.502 | 1826.707 ± 592.457 |
| Nigeria | 266676.916 | 370366.061 | 438185.821 | 552582.909 | 474904.943 ± 158048.464 |
| Niue | 0.414 | 0.436 | 0.518 | 0.728 | 0.584 ± 0.173 |
| Oman | 113.504 | 192.583 | 223.943 | 270.032 | 620.129 ± 1400.784 |
| Pakistan | 503467.805 | 592638.411 | 691074.009 | 1446015.297 | 950729.404 ± 460085.899 |
| Palau | 410.389 | 457.04 | 802.853 | 3497.179 | 5217.51 ± 12060.014 |
| Panama | 8905.742 | 14994.072 | 19318.328 | 21480.574 | 19781.167 ± 7579.171 |
| Papua New Guinea | 154.773 | 182.229 | 213.924 | 507.279 | 474.551 ± 485.218 |
| Paraguay | 38373.415 | 45359.182 | 59651.759 | 97810.322 | 74378.973 ± 36632.896 |
| Peru | 49428.802 | 69505.069 | 105472.862 | 125732.53 | 108453.496 ± 47754.404 |
| Philippines | 74914.422 | 130113.308 | 257684.581 | 526279.844 | 396431.967 ± 366833.917 |
| Rwanda | 589.487 | 653.759 | 1174.942 | 1497.849 | 1734.846 ± 1828.417 |
| Saint Kitts and Nevis | 14.444 | 17.281 | 40.892 | 46.13 | 34.282 ± 14.156 |
| Saint Lucia | 191.018 | 197.743 | 247.737 | 356.171 | 292.234 ± 115.402 |
| Saint Vincent and the Grenadines | 167.448 | 193.598 | 388.189 | 536.583 | 382.093 ± 179.258 |
| Samoa | 389.694 | 423.552 | 460.152 | 691.452 | 634.065 ± 339.207 |
| Sao Tome and Principe | 786.027 | 1060.781 | 1181.857 | 1223.58 | 1113.596 ± 153.213 |
| Saudi Arabia | 60.897 | 80.525 | 157.883 | 342.117 | 1190.769 ± 3919.346 |
| Senegal | 1642.739 | 2049.704 | 2780.688 | 3243.354 | 2762.675 ± 779.672 |
| Seychelles | 4488.827 | 7930.643 | 8687.086 | 9631.015 | 9426.237 ± 2933.799 |
| Sierra Leone | 9505.793 | 13196.327 | 14529.959 | 17344.207 | 15287.63 ± 3678.727 |
| Singapore | 116330.858 | 297887.523 | 384469.202 | 505761.676 | 730700.377 ± 1353394.837 |
| Solomon Islands | 885.829 | 984.685 | 1114.292 | 1688.803 | 1450.949 ± 685.435 |
| Somalia | 1546.573 | 1735.616 | 1911.834 | 2199.882 | 2000.865 ± 397.587 |
| South Sudan | 13.948 | 21.986 | 28.225 | 95.58 | 184.676 ± 363.313 |
| Sri Lanka | 51499.746 | 67905.201 | 148676.362 | 352130.665 | 231538.949 ± 212358.128 |
| Sudan | 1411.407 | 1755.698 | 2183.206 | 2861.975 | 2322.188 ± 657.953 |
| Suriname | 855.953 | 1527.464 | 1694.888 | 2850.2 | 2065.744 ± 981.496 |
| Syrian Arab Republic | 129.608 | 204.893 | 402.31 | 1886.965 | 1038.243 ± 1113.113 |
| Thailand | 167353.271 | 203819.362 | 387054.697 | 1263298.452 | 852661.263 ± 931943.754 |
| Timor-Leste | 83.713 | 185 | 195.018 | 1262.549 | 625.404 ± 630.633 |
| Togo | 8534.165 | 9976.843 | 10732.918 | 15117.041 | 12694.422 ± 3937.115 |
| Tonga | 3073.533 | 25943.988 | 28508.541 | 33242.828 | 63255.591 ± 118561.884 |
| Trinidad and Tobago | 23049.336 | 27563.272 | 29898.874 | 33866.635 | 35441.784 ± 22404.16 |
| Tuvalu | 5.706 | 8.912 | 16.816 | 41.525 | 43.976 ± 60.792 |
| Uganda | 4415.047 | 5191.057 | 5757.373 | 6770.72 | 6803.89 ± 3021.182 |
| United Republic of Tanzania | 898.53 | 1005.387 | 1817.509 | 3542.812 | 3606.267 ± 4909.073 |
| United States of America | 30.223 | 37.171 | 152.526 | 239.289 | 362.524 ± 659.178 |
| Vanuatu | 208.251 | 232.476 | 367.167 | 826.429 | 518.411 ± 319.799 |
| Venezuela (Bolivarian Republic of) | 113381.863 | 124724.838 | 147691.286 | 230887.061 | 188629.761 ± 85072.828 |
| Viet Nam | 497531.575 | 598958.193 | 624058.767 | 708045.734 | 709296.19 ± 212089.307 |
| Yemen | 2714.096 | 3981.695 | 4411.547 | 5282.118 | 4948.813 ± 1755.594 |
| Zambia | 1579.063 | 1736.329 | 2220.713 | 2617.735 | 2364.467 ± 816.121 |
| Zimbabwe | 581.772 | 630.379 | 847.029 | 949.939 | 809.402 ± 154.957 |

*Summary statistics include minimum (Min), first quartile (Q1), median, third quartile (Q3), and mean ± standard deviation (Mean ± SD) of dengue incidence per country. Countries with any zero incidence over 2000–2021 were excluded.*

**Table S10.** Performance comparison of deep learning models (ConvLSTM, ANN, STCNN, FedFormer, and STGNN) in predicting dengue incidence across both sexes, males, and females from 2000 to 2021.

|  | **Set** | **RMSE** | **R2** | **AdjR2** | **MAE** |
| --- | --- | --- | --- | --- | --- |
| **Both** |  |  |  |  |  |
| **ConvLSTM** | **Train (5-CV avg)** | **609781.40179** | **0.86990** | **0.86587** | **81696.39063** |
|  | **Test (2018–2021)** | **1283892.598** | **0.676336058** | **0.675897489** | **235885.9489** |
| ANN | Train (5-CV avg) | 1359205.65302 | 0.34327 | 0.32291 | 326074.56250 |
|  | Test (2018–2021) | 1578933.31700 | 0.51049 | 0.49687 | 452054.80488 |
| STCNN | Train (5-CV avg) | 1044555.794 | 0.637606418 | 0.637060645 | 176745.8594 |
|  | Test (2018–2021) | 1387407.13633 | 0.62204 | 0.61153 | 183887.33599 |
| FedFormer | Train (2000–2017) | 796971.2548 | 0.814285457 | 0.813049981 | 240904.75 |
|  | Test (2018–2021) | 1460519.6 | 0.581156552 | 0.568305009 | 341181.0625 |
| STGNN | Train (2000–2017) | 1842718.088 | 0.007161856 | 0.001161039 | 247102.6875 |
|  | Test (2018–2021) | 2257478.429 | -0.000654817 | -0.02848947 | 305841.1563 |
| **Male** |  |  |  |  |  |
| **ConvoLSTM** | **Train (5-CV avg)** | **283209.5595** | **0.887053287** | **0.883551063** | **36794.40234** |
|  | **Test (2018–2021)** | **619585.9931** | **0.663525224** | **0.663069296** | **109620.2539** |
| ANN | Train (5-CV avg) | 611393.976 | 0.388402319 | 0.36943805 | 146678.625 |
|  | Test (2018–2021) | 733579.9541 | 0.528323498 | 0.51520315 | 208553.0344 |
| STCNN | Train (5-CV avg) | 434022.1146 | 0.690038848 | 0.689572039 | 75114.46094 |
|  | Test (2018–2021) | 715281.5795 | 0.551560962 | 0.539086997 | 89868.75506 |
| FedFormer | Train (2000–2017) | 593753.3283 | 0.537675977 | 0.534600341 | 107002.1016 |
|  | Test (2018–2021) | 715675.0487 | 0.551067472 | 0.537292694 | 131161.625 |
| STGNN | Train (2000–2017) | 878053.0935 | -0.011057496 | -0.017168403 | 119022.8672 |
|  | Test (2018–2021) | 1075052.424 | -0.012998343 | -0.041176319 | 143645.1094 |
| **Female** |  |  |  |  |  |
| **ConvLSTM** | **Train (2000–2017)** | **271285.8767** | **0.922933877** | **0.92242119** | **95343.125** |
|  | **Test (2018–2021)** | **456788.1537** | **0.852497697** | **0.847971824** | **123378.9297** |
| ANN | Train (5-CV avg) | 702821.7002 | 0.375317144 | 0.355947133 | 169193.8594 |
|  | Test (2018–2021) | 832312.4911 | 0.510286569 | 0.496664499 | 245354.177 |
| STCNN | Train (5-CV avg) | 565158.3843 | 0.620996416 | 0.620425627 | 96907.85938 |
|  | Test (2018–2021) | 564023.4977 | 0.775113396 | 0.774808672 | 165497.7028 |
| FedFormer | Train (5-CV avg) | 425227.7855 | 0.803192723 | 0.797090172 | 57802.63281 |
|  | Test (2018–2021) | 1038040.182 | 0.238276209 | 0.217087786 | 160536.5732 |
| STGNN | Train (2000–2017) | 975243.55 | 0.004055262 | -0.001964331 | 132518.1563 |
|  | Test (2018–2021) | 1191385.495 | -0.003399372 | -0.03131032 | 164307.1094 |

*RMSE :Root Mean Square Error; MAE :Mean Absolute Error; R² :Coefficient of Determination; Adj. R² :Adjusted Coefficient of Determination; MAPE :Mean Absolute Percentage Error; ANN :Artificial Neural Network; ConvLSTM :Convolutional Long Short-Term Memory; STCNN :Spatiotemporal Convolutional Neural Network; FedFormer :Federated Transformer; STGNN :Spatiotemporal Graph Neural Network.*


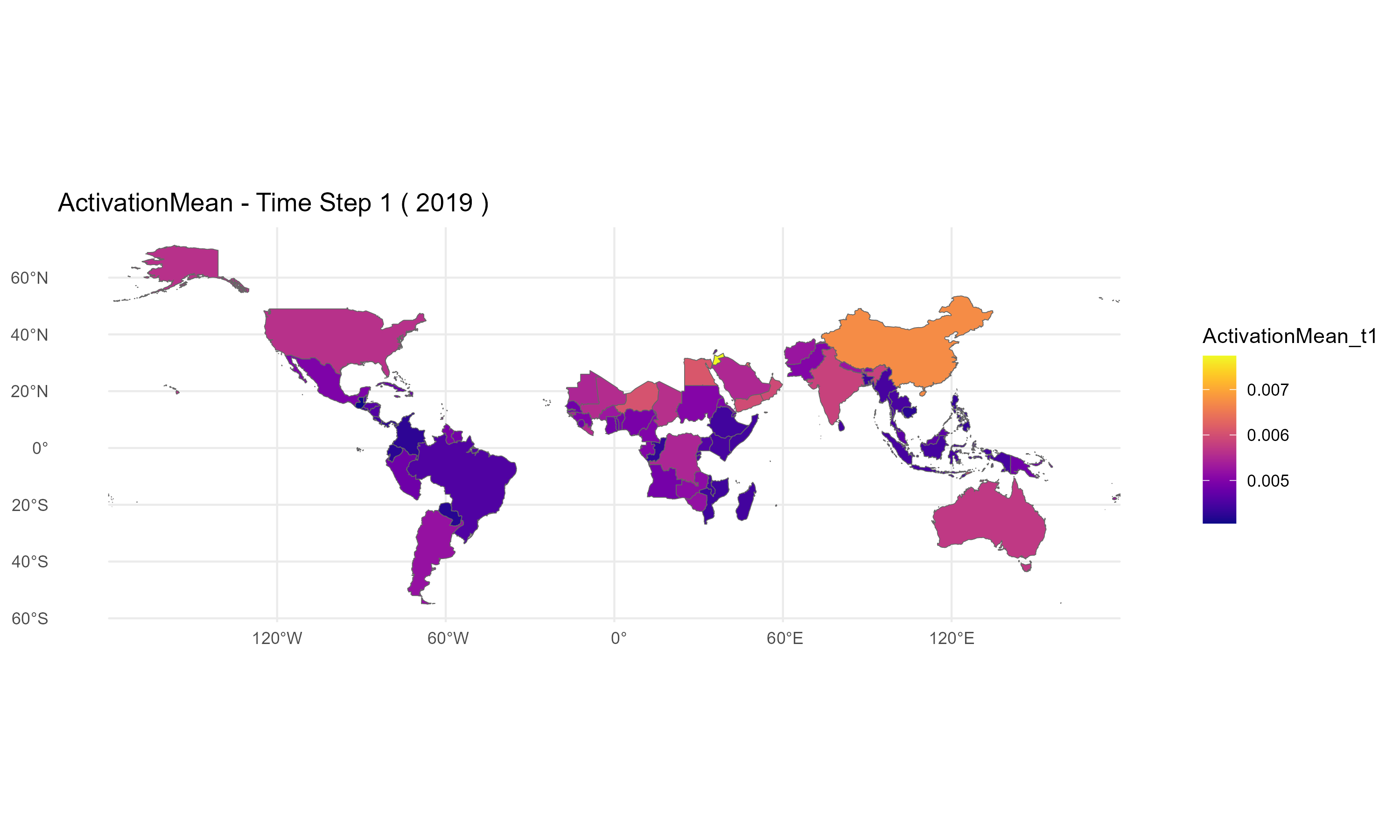

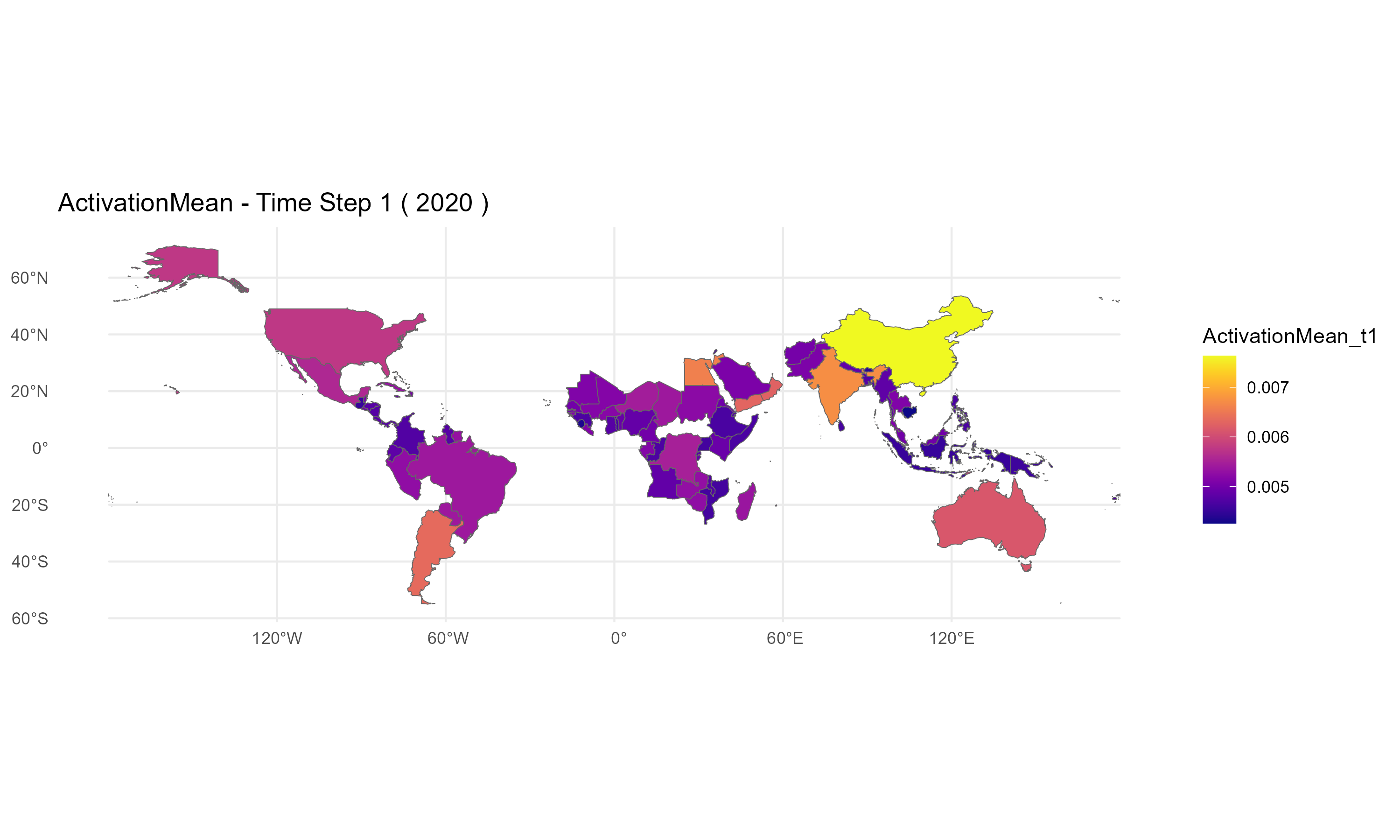

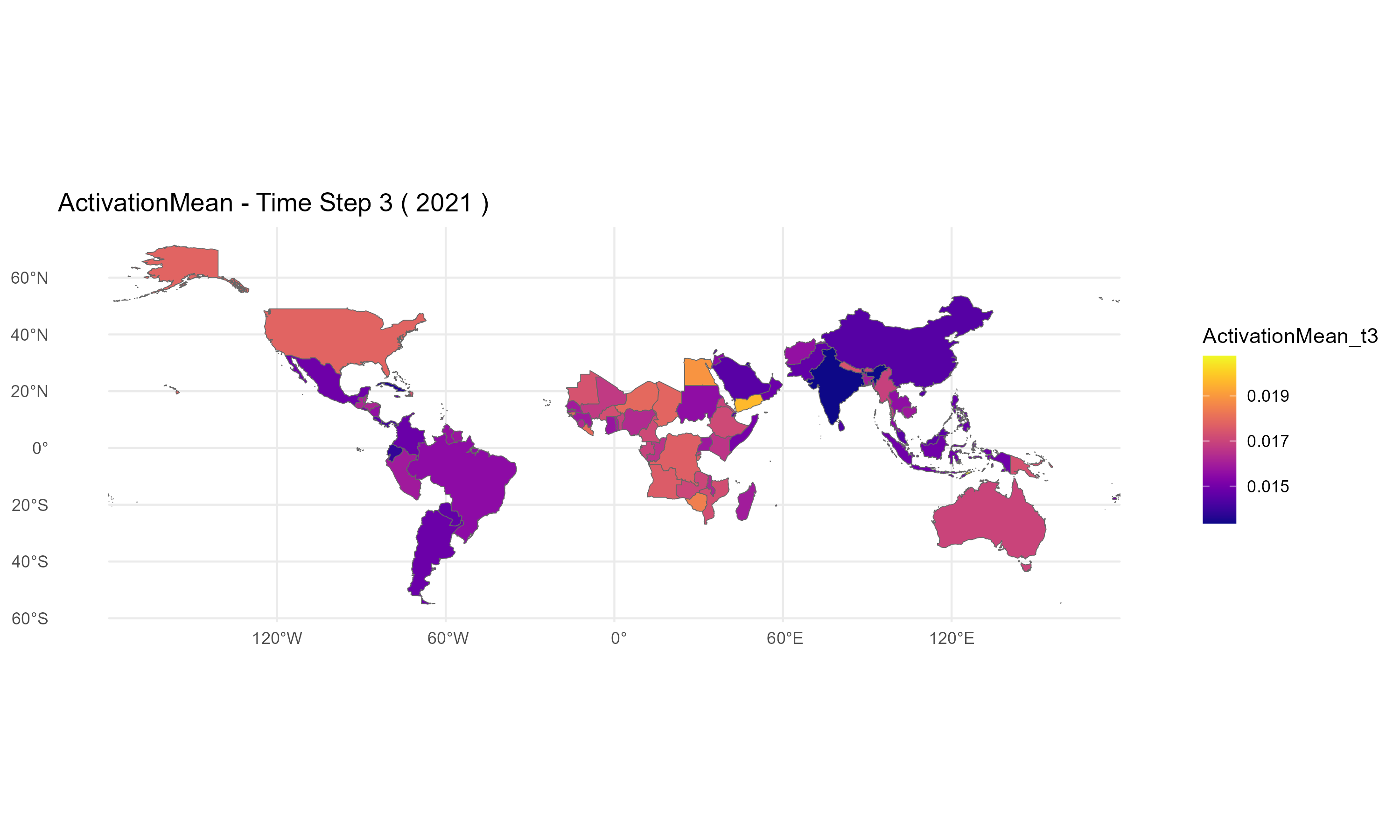

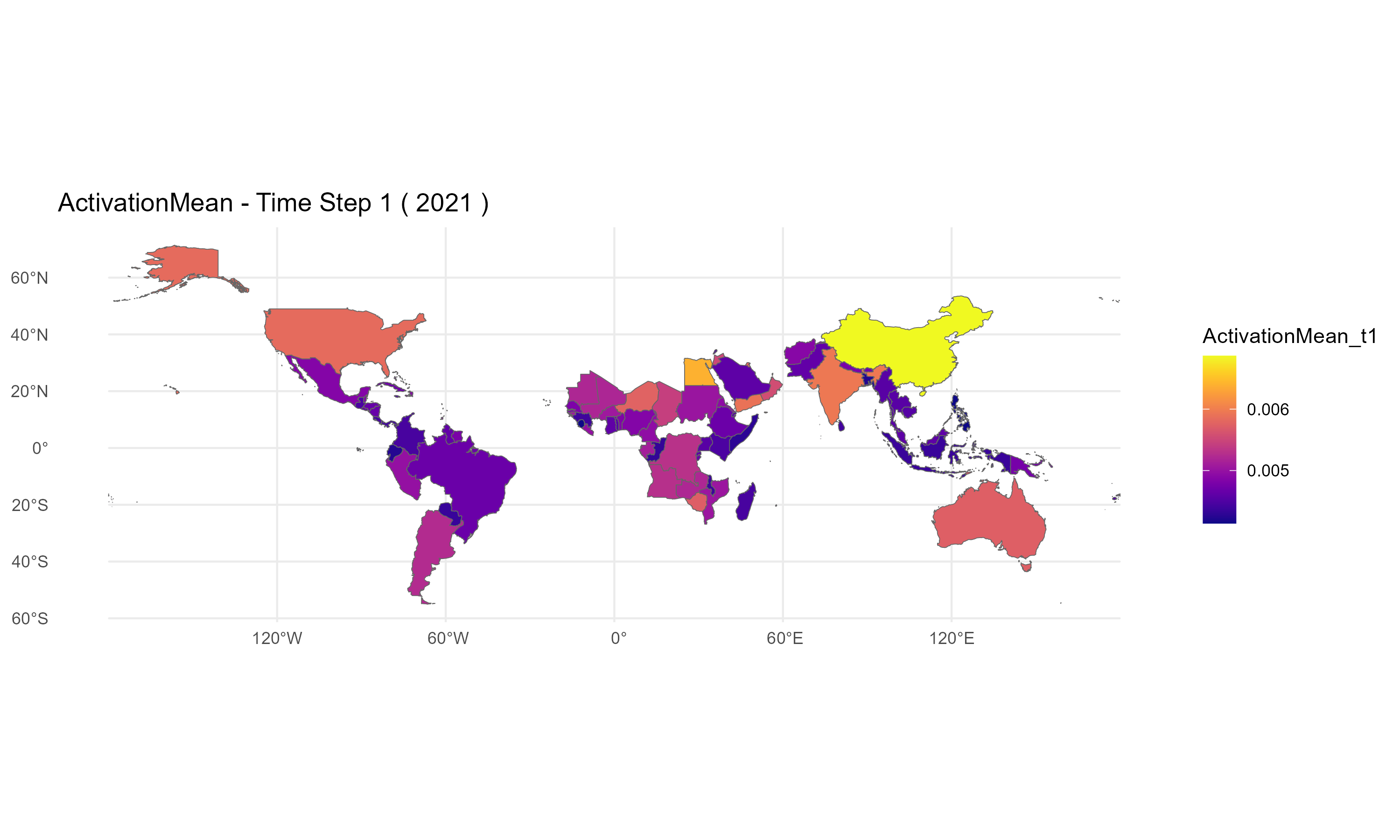

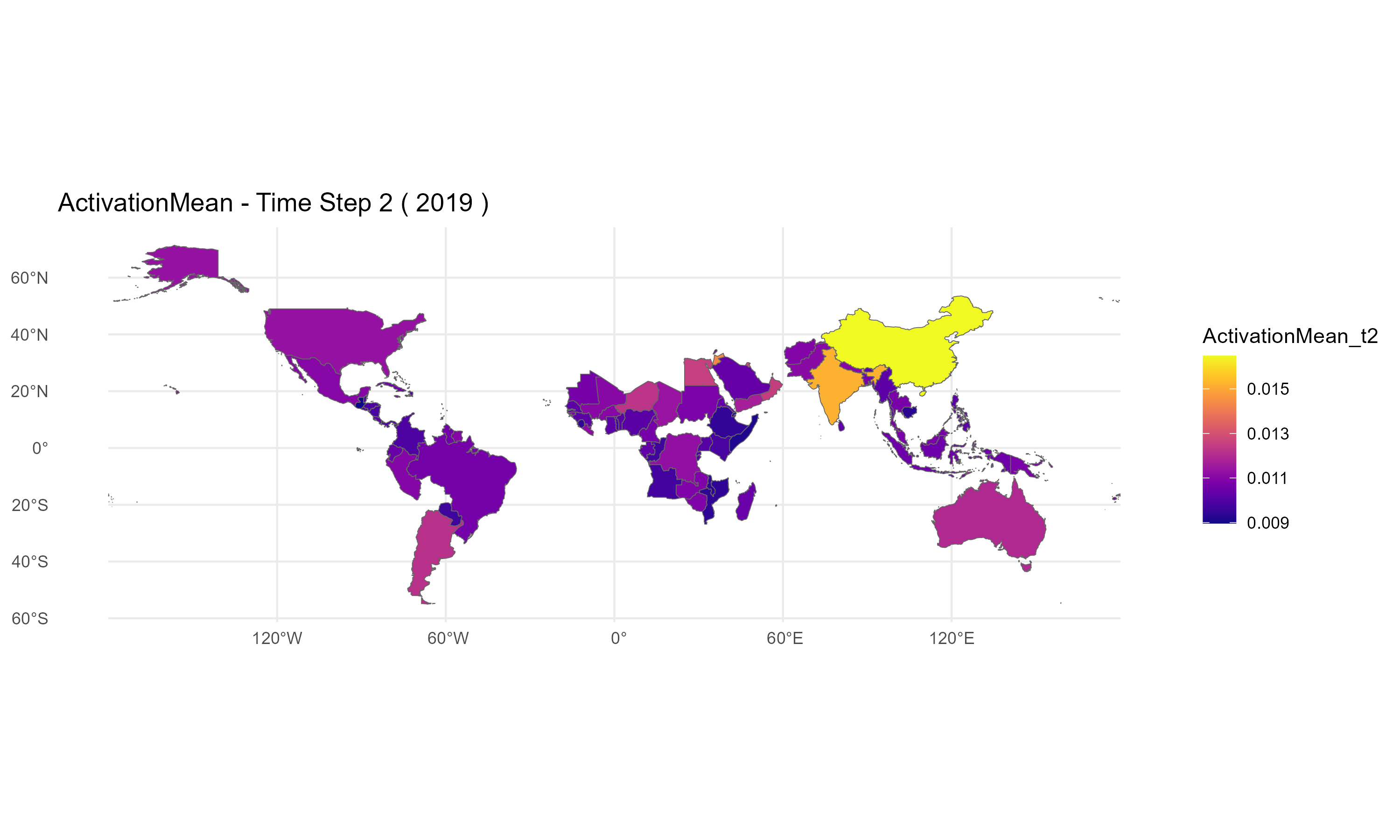

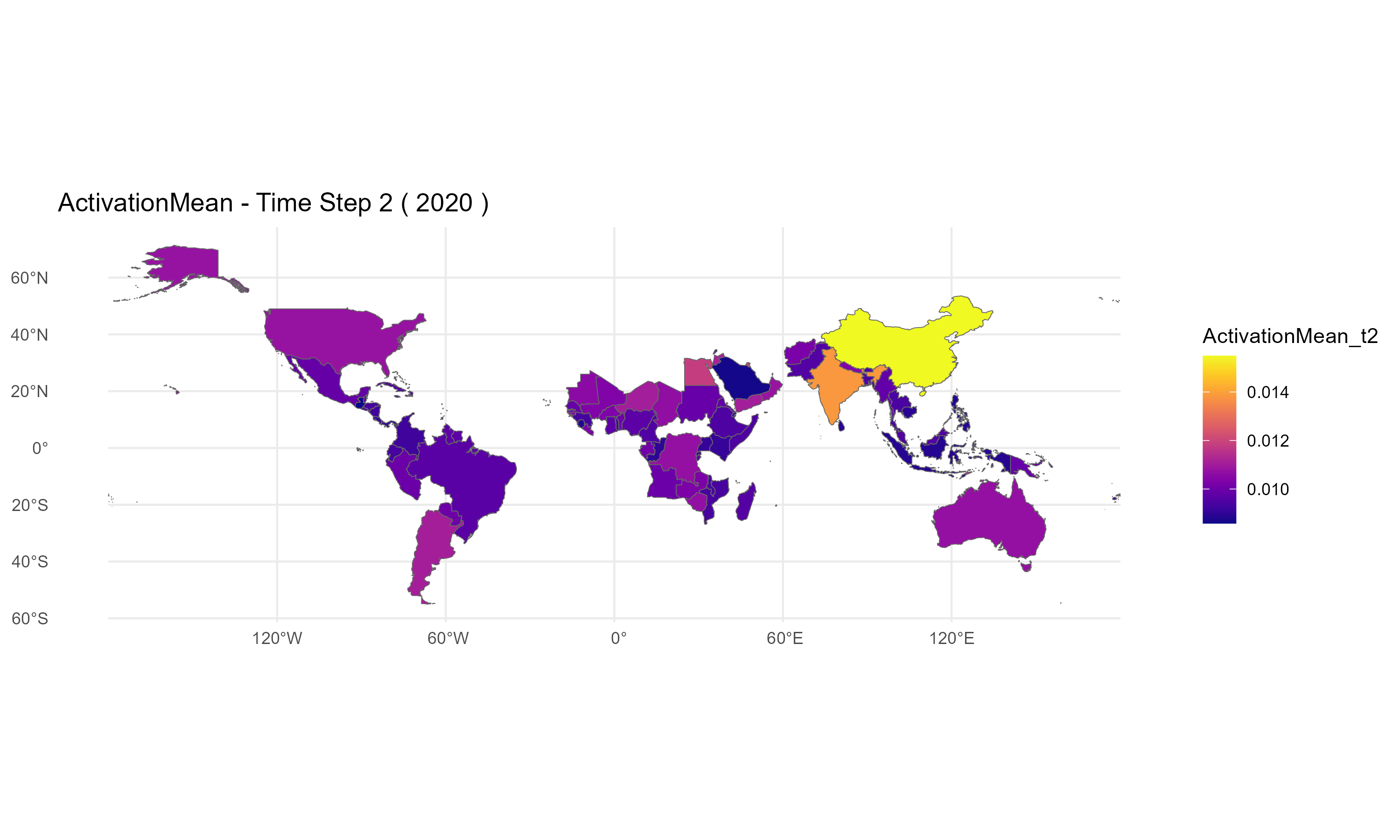

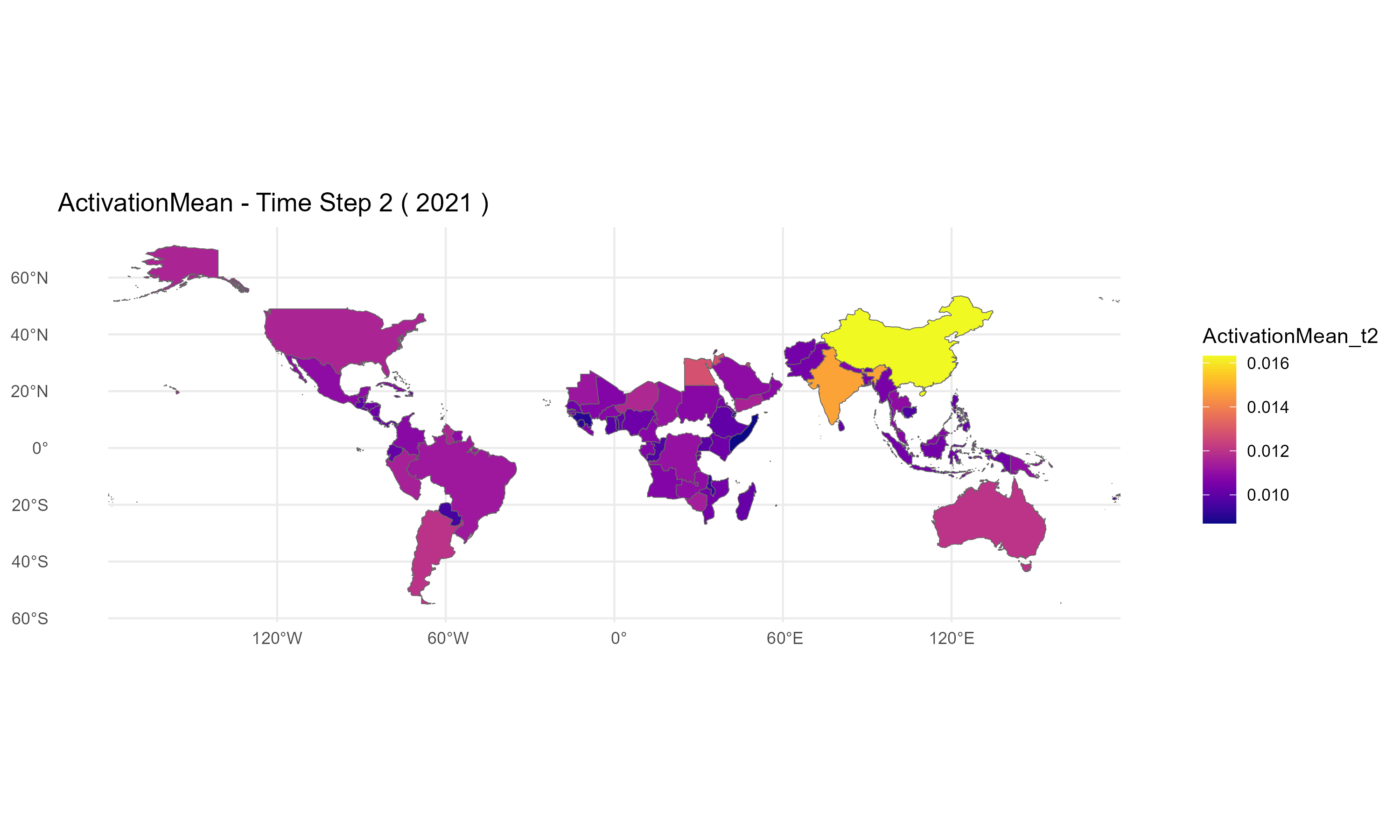

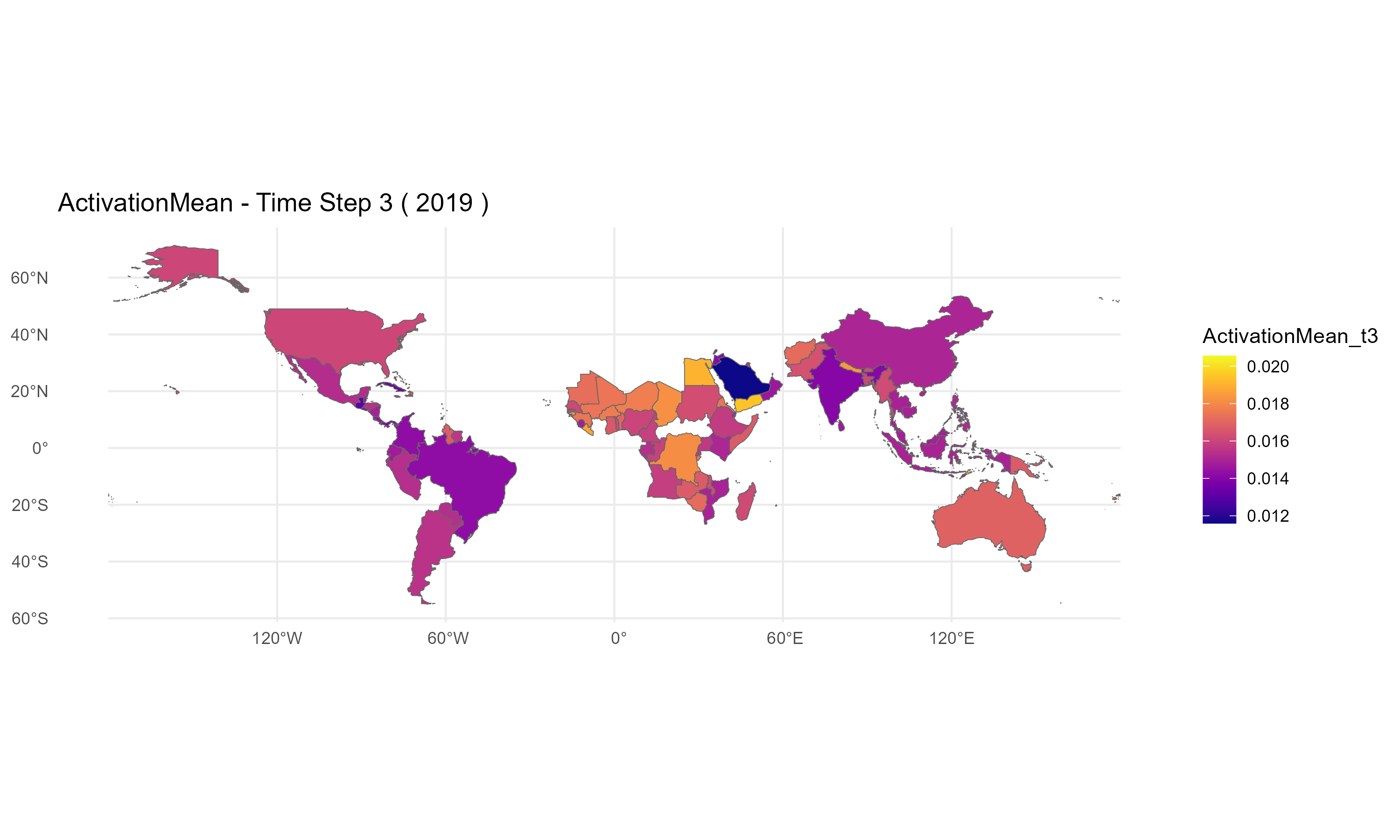

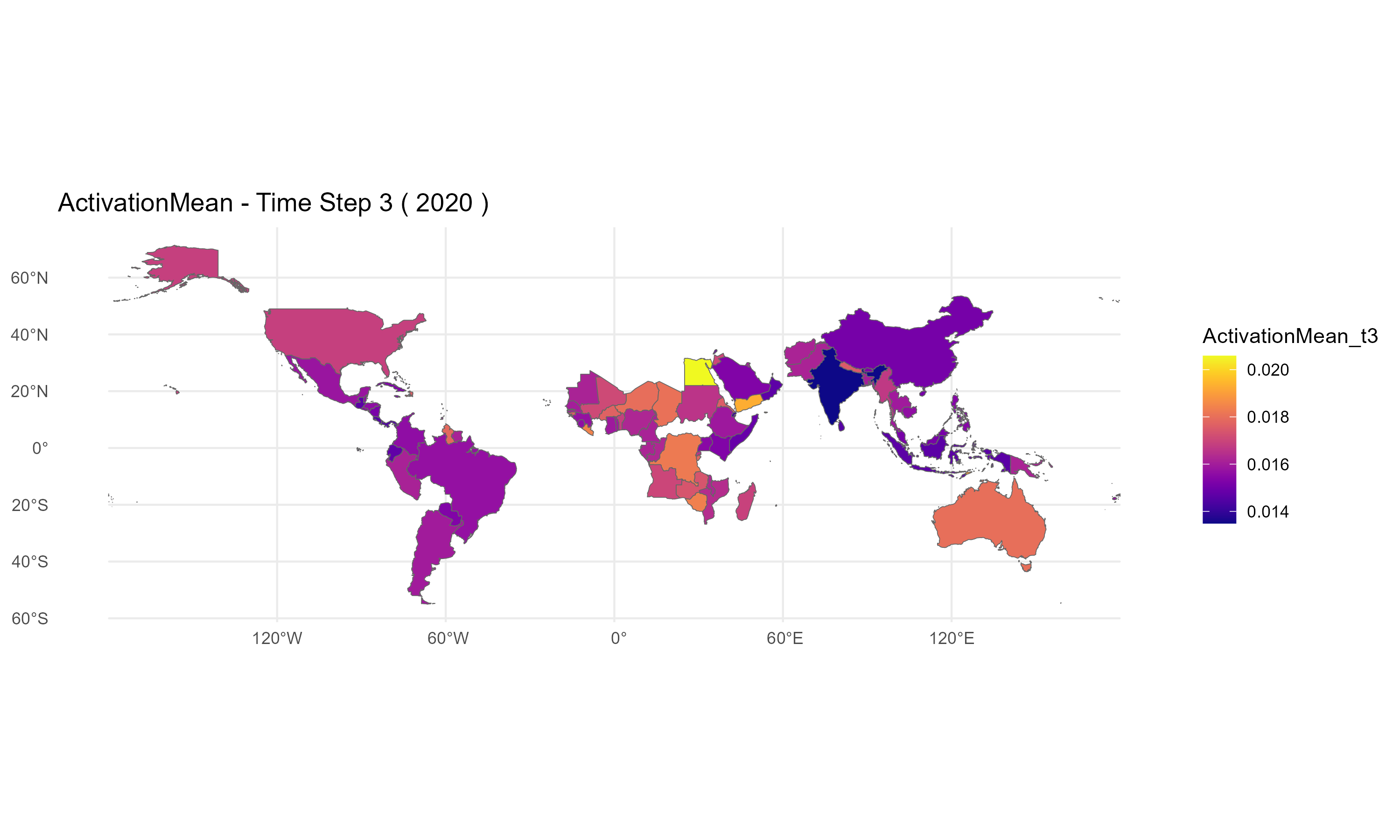

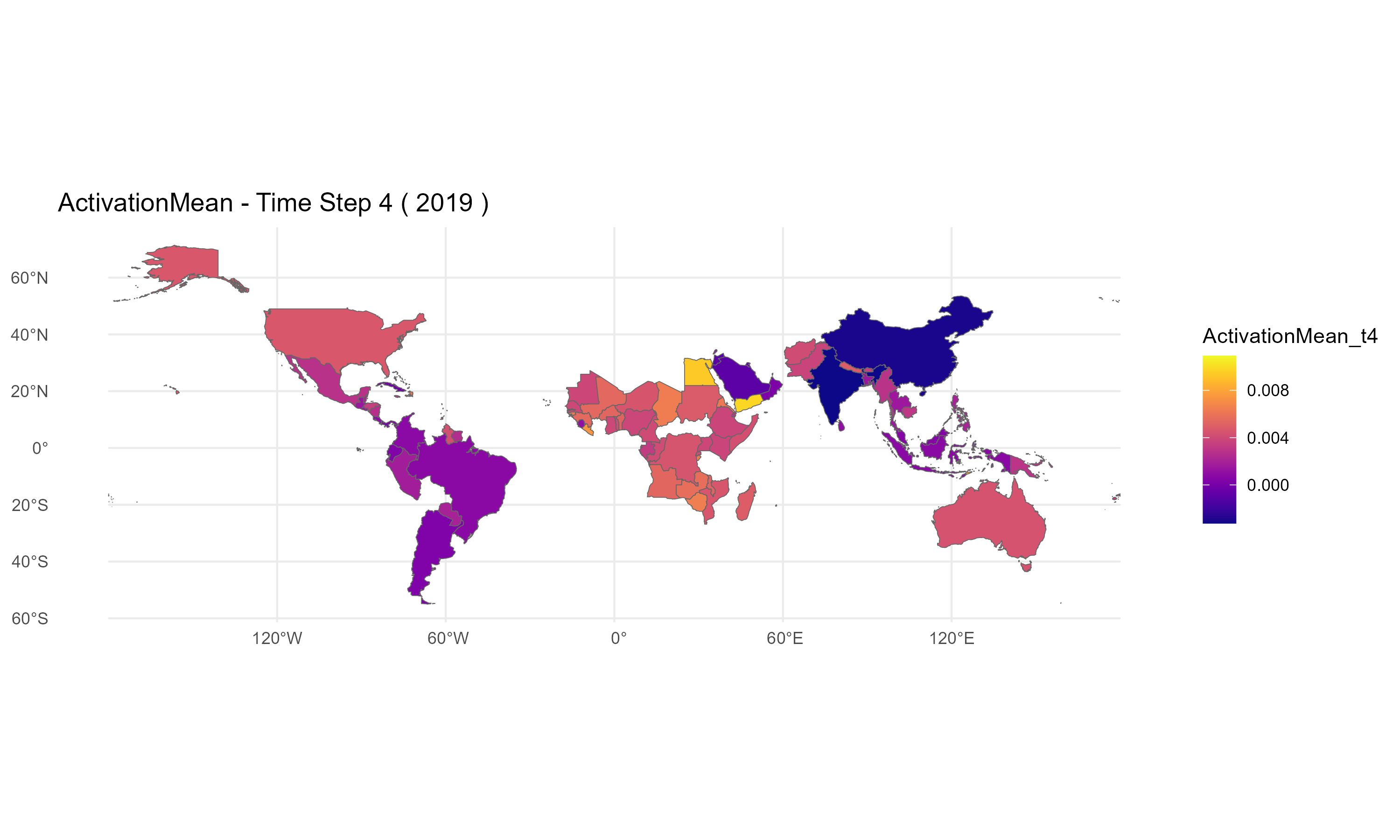

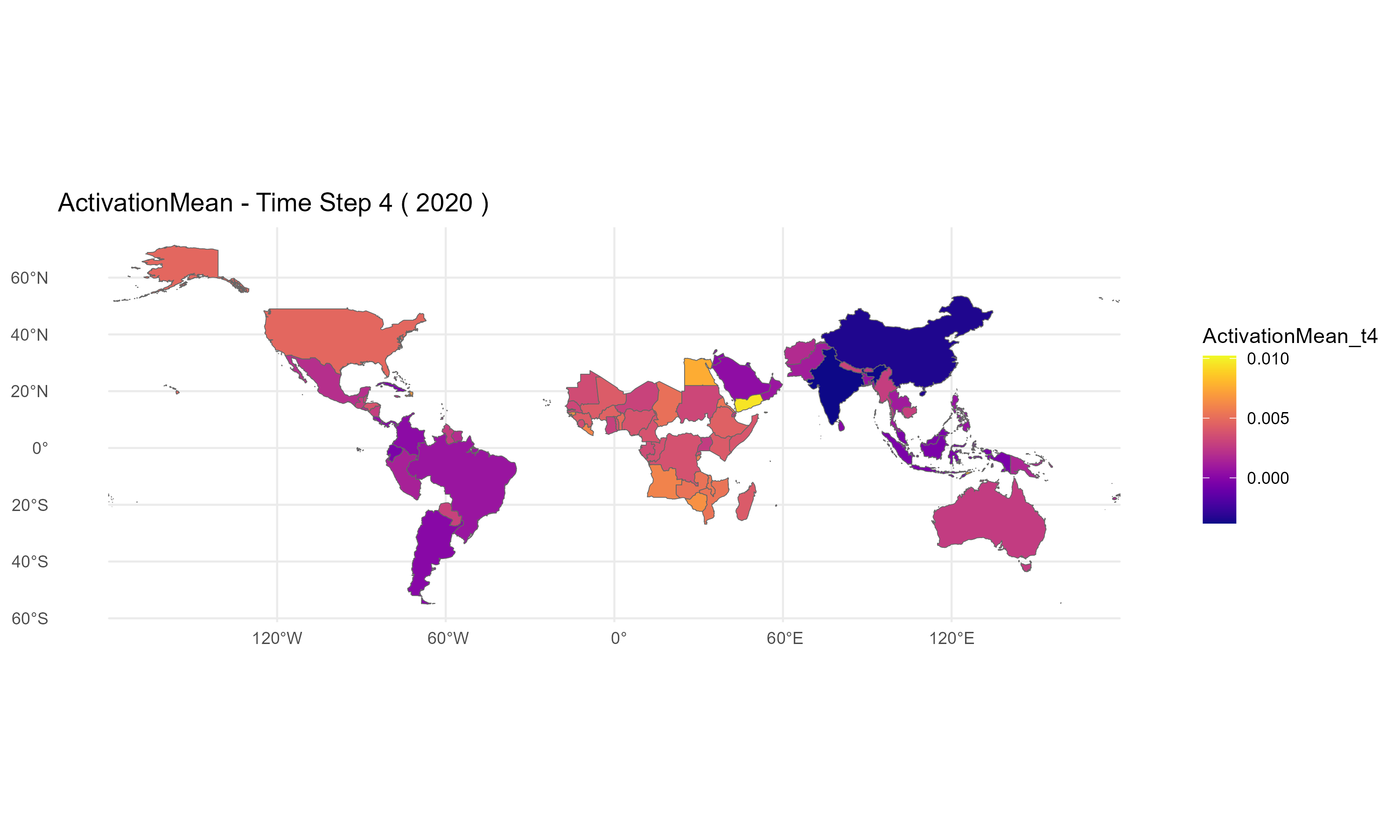

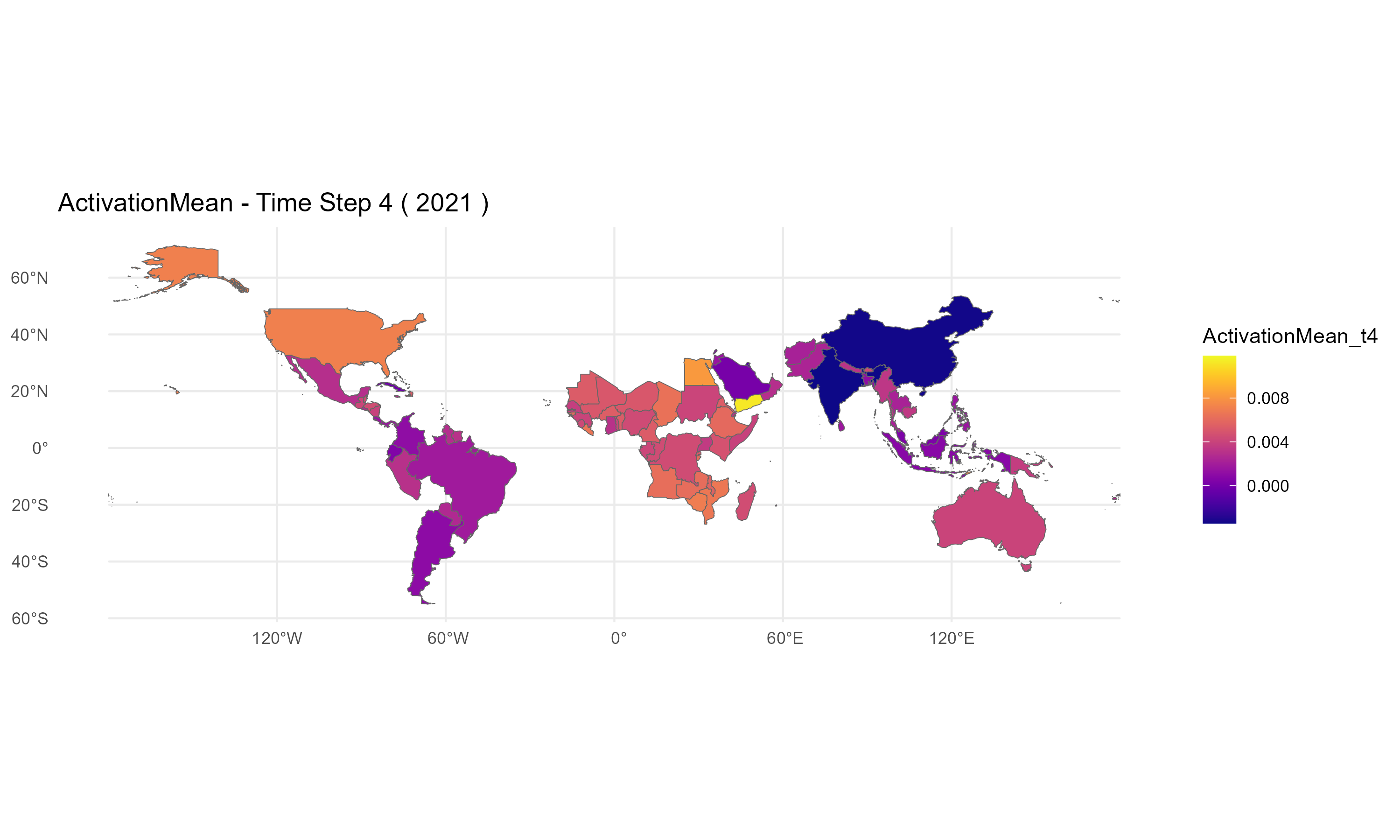

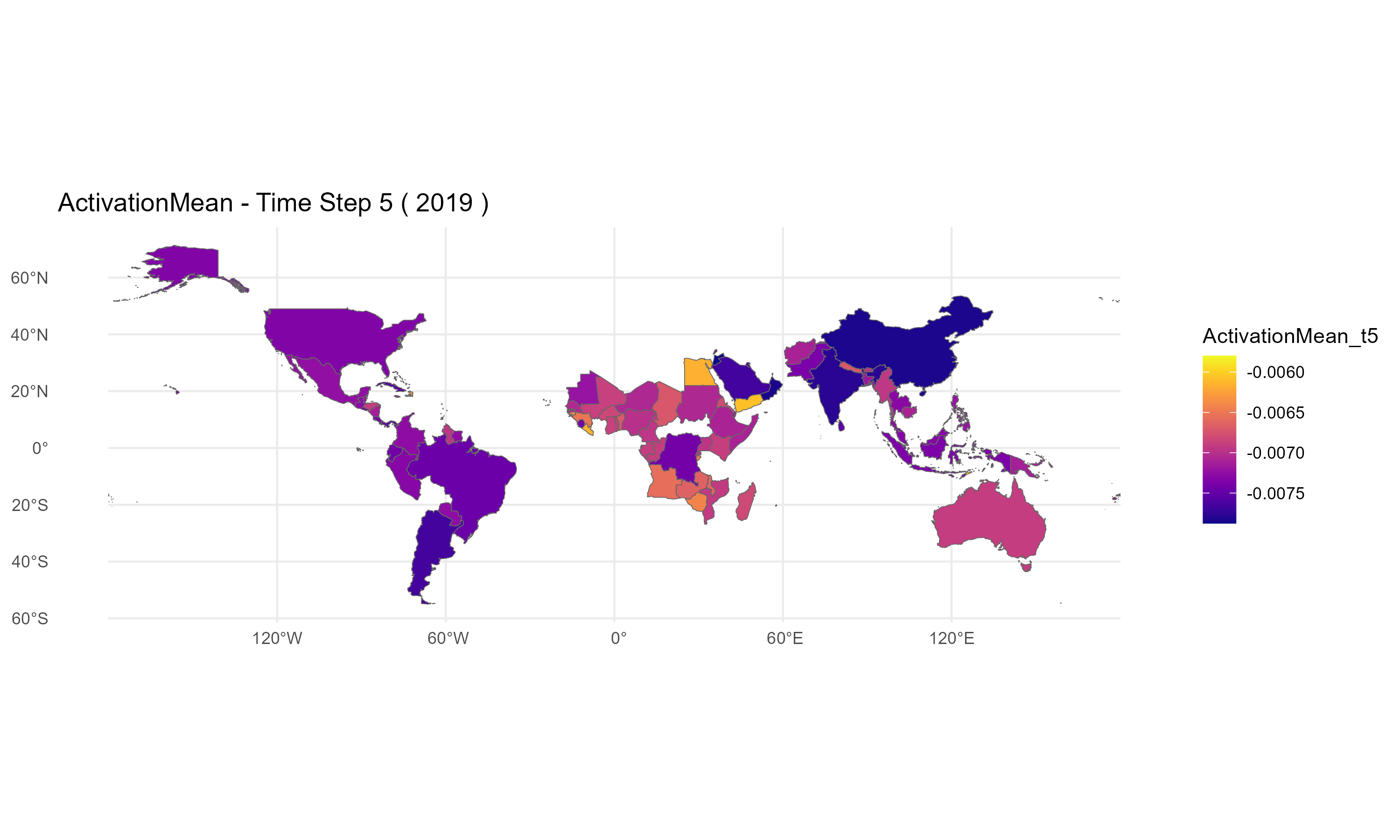

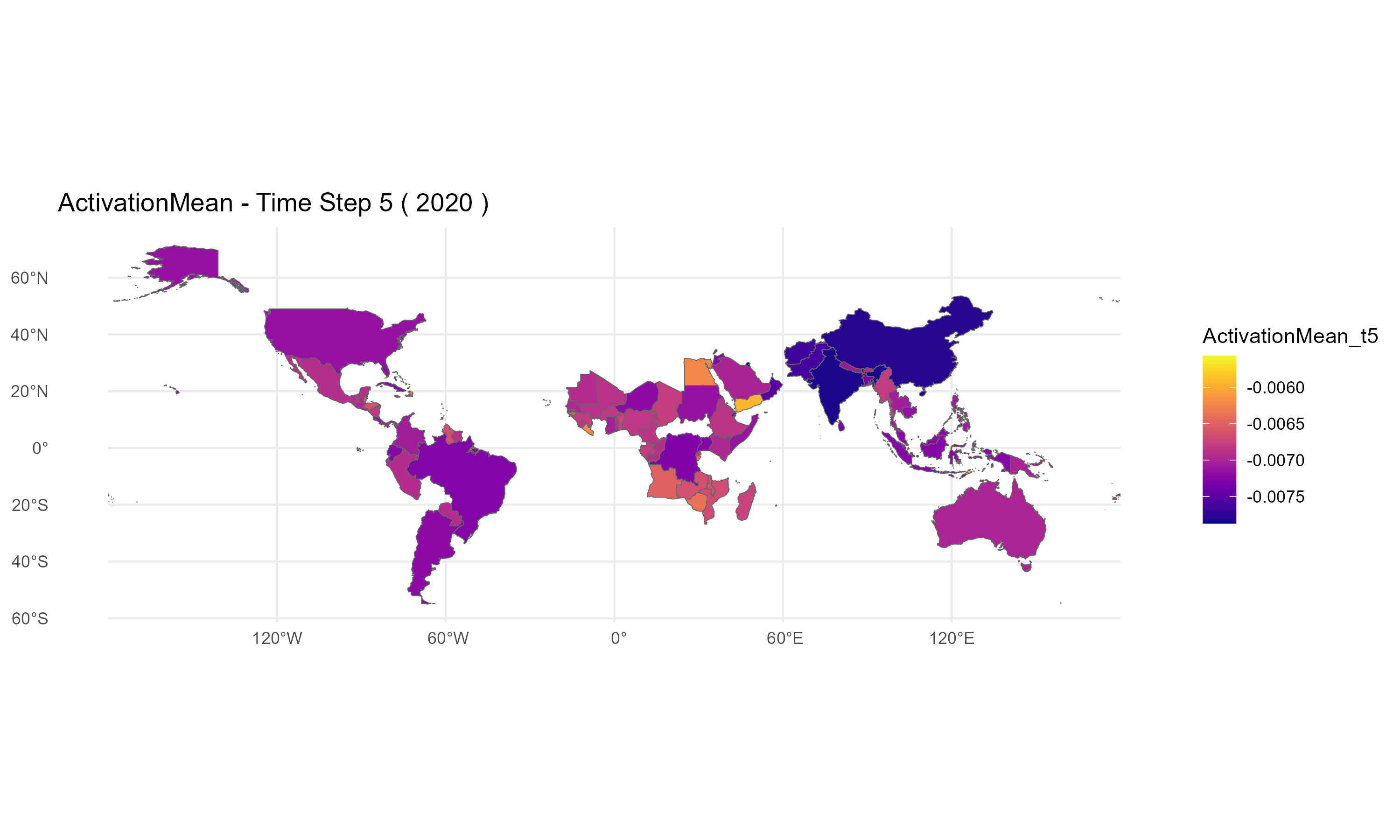

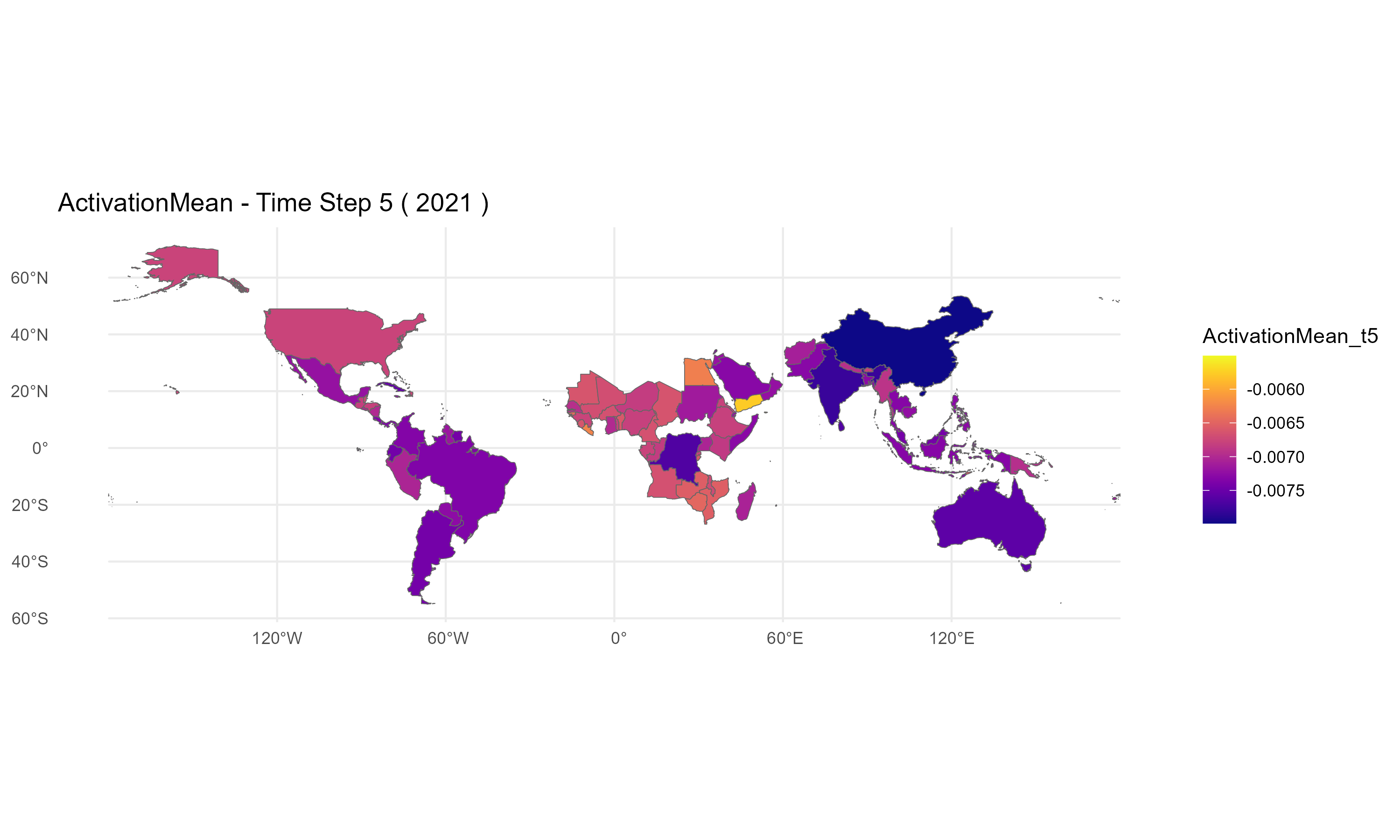

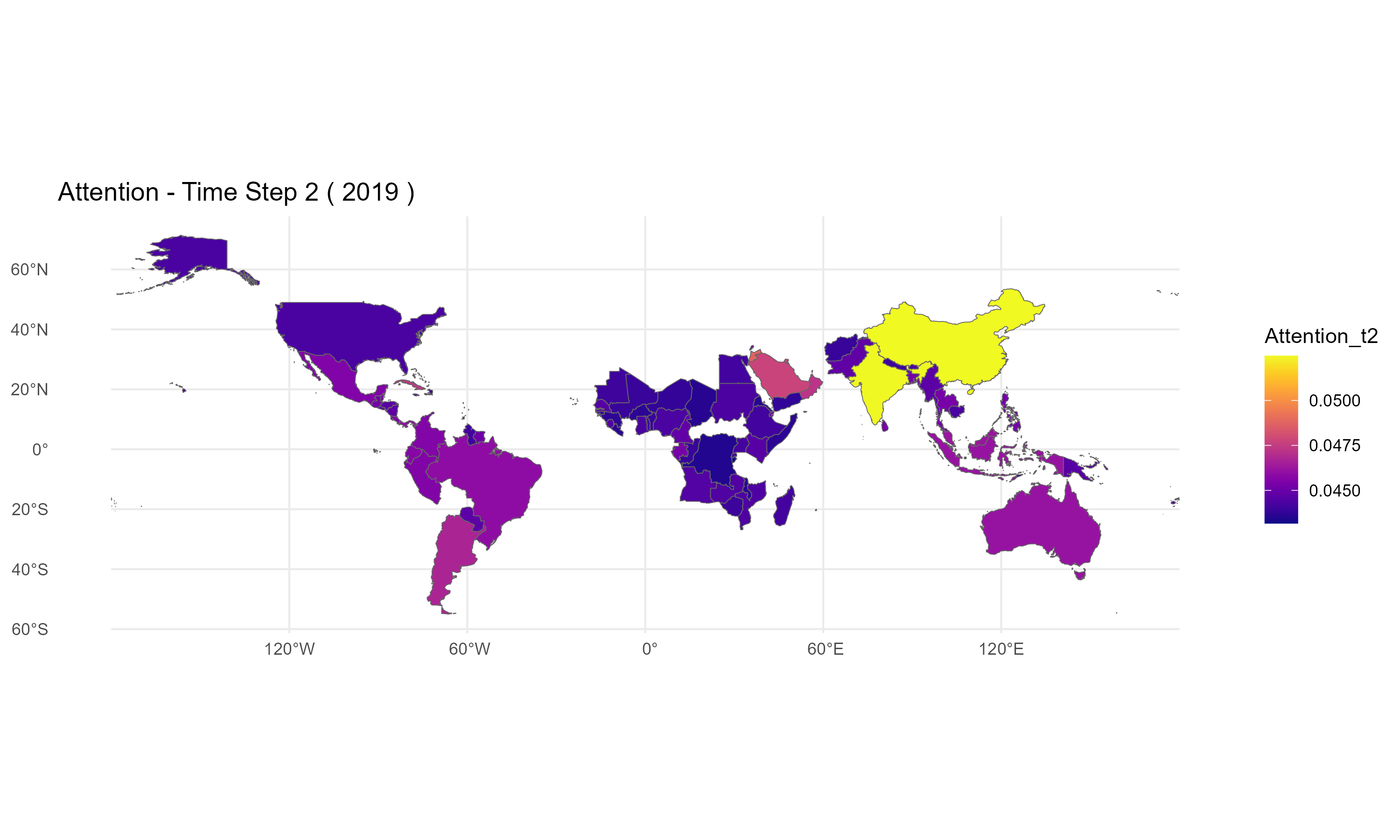

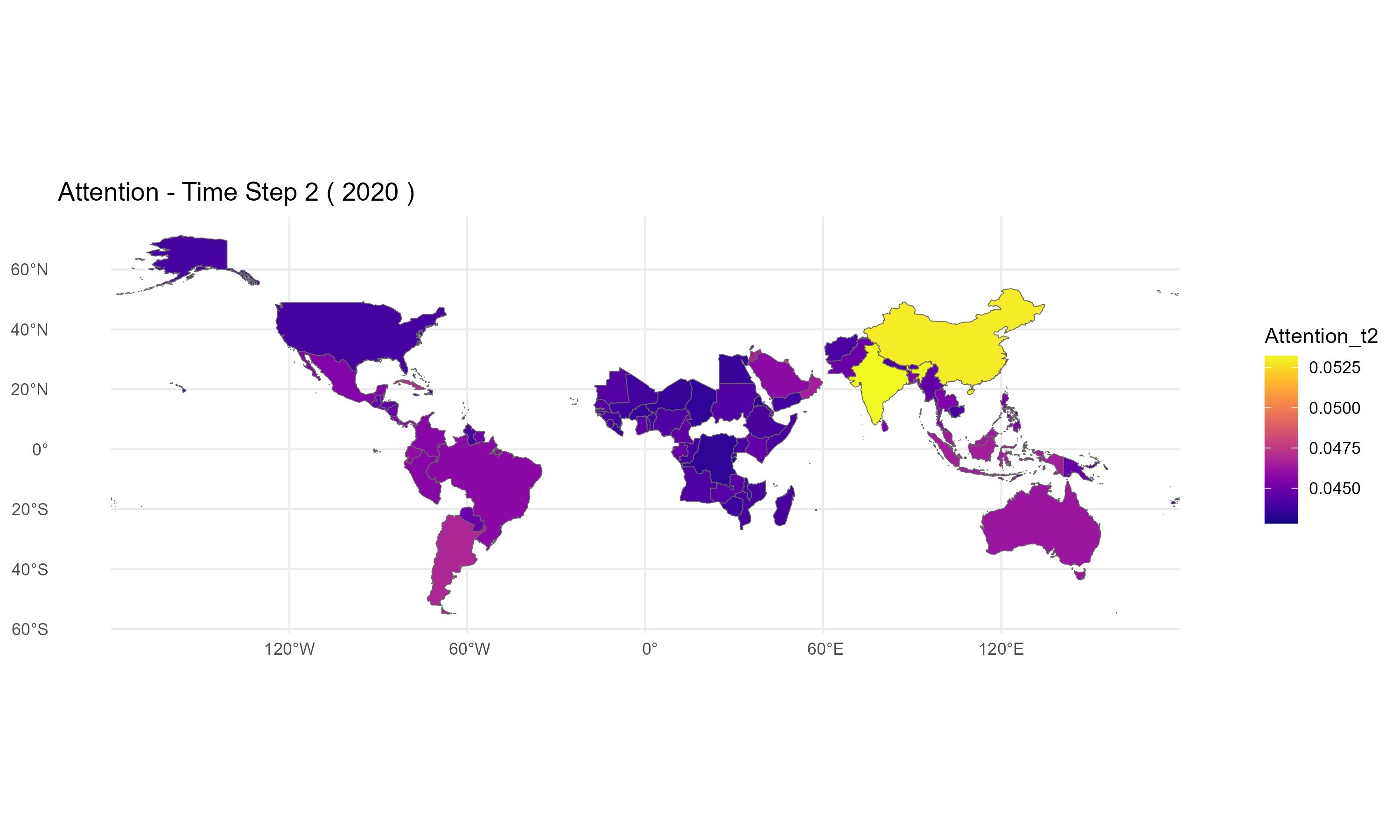

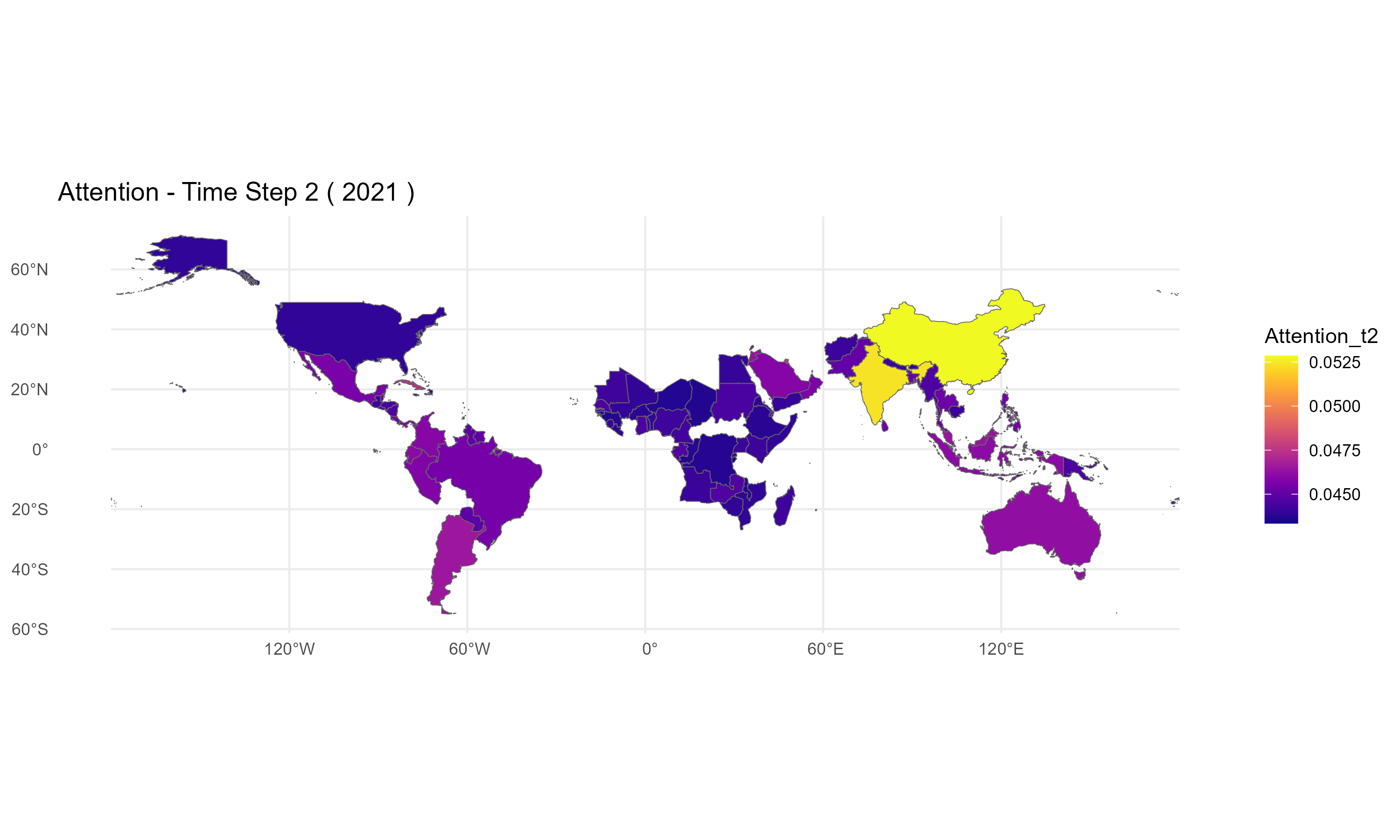

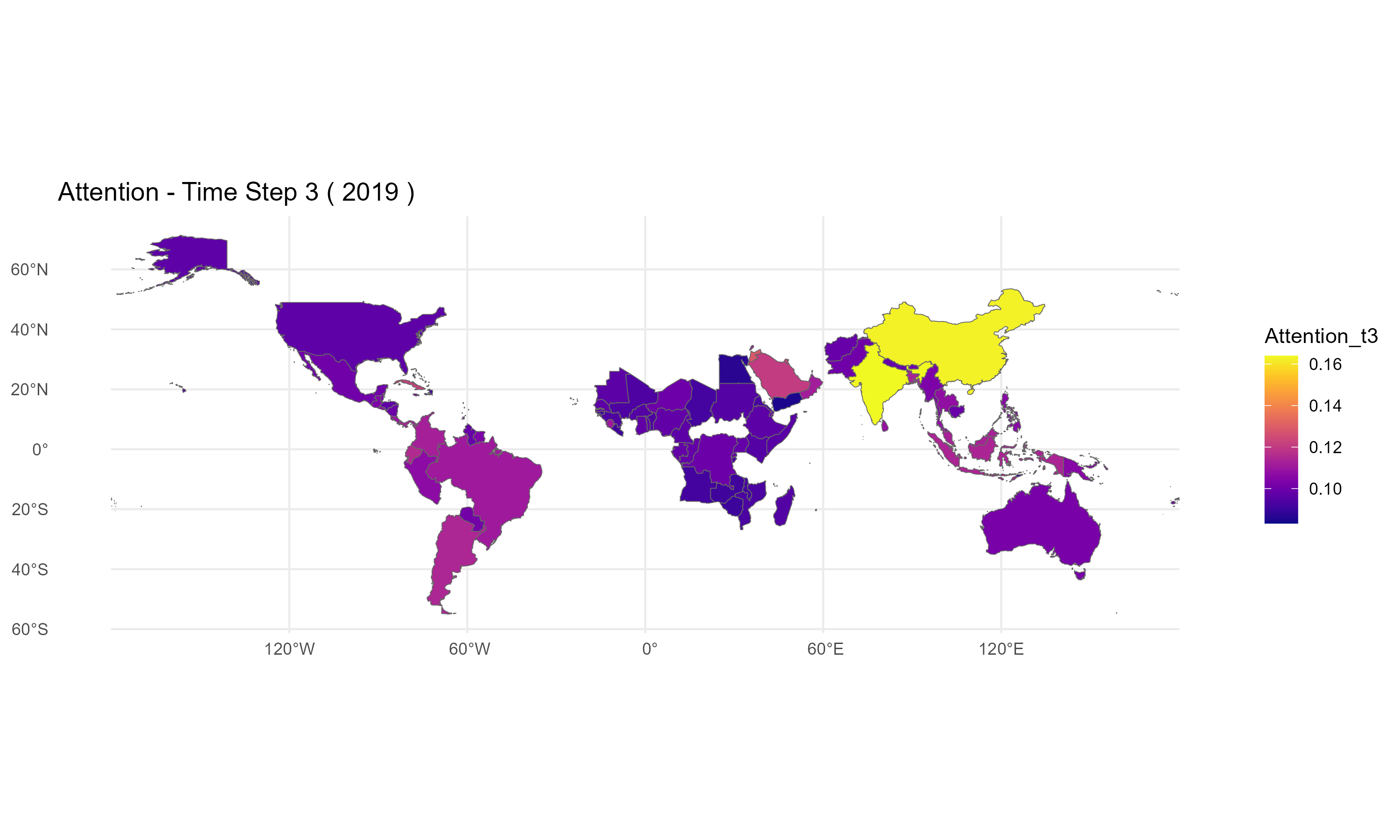

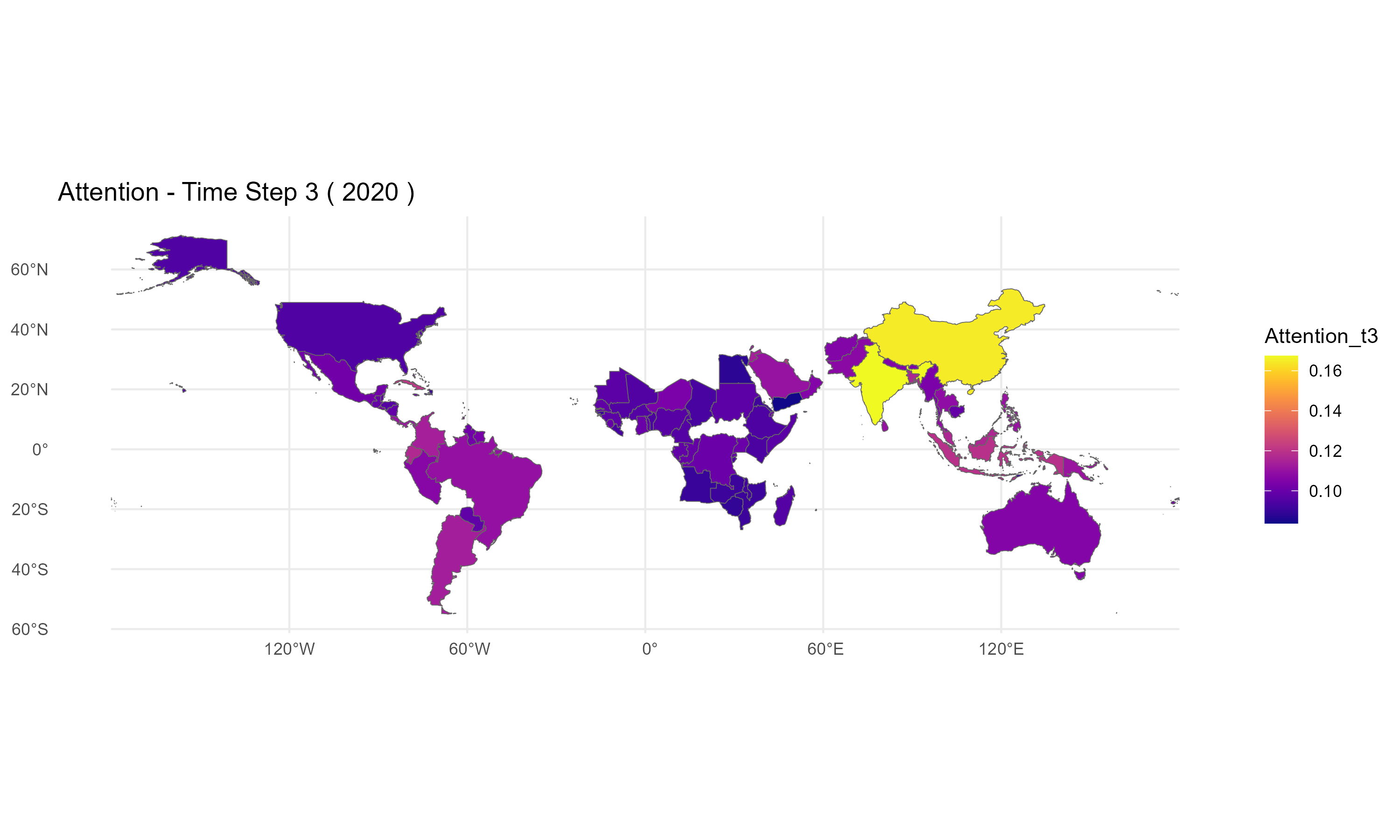

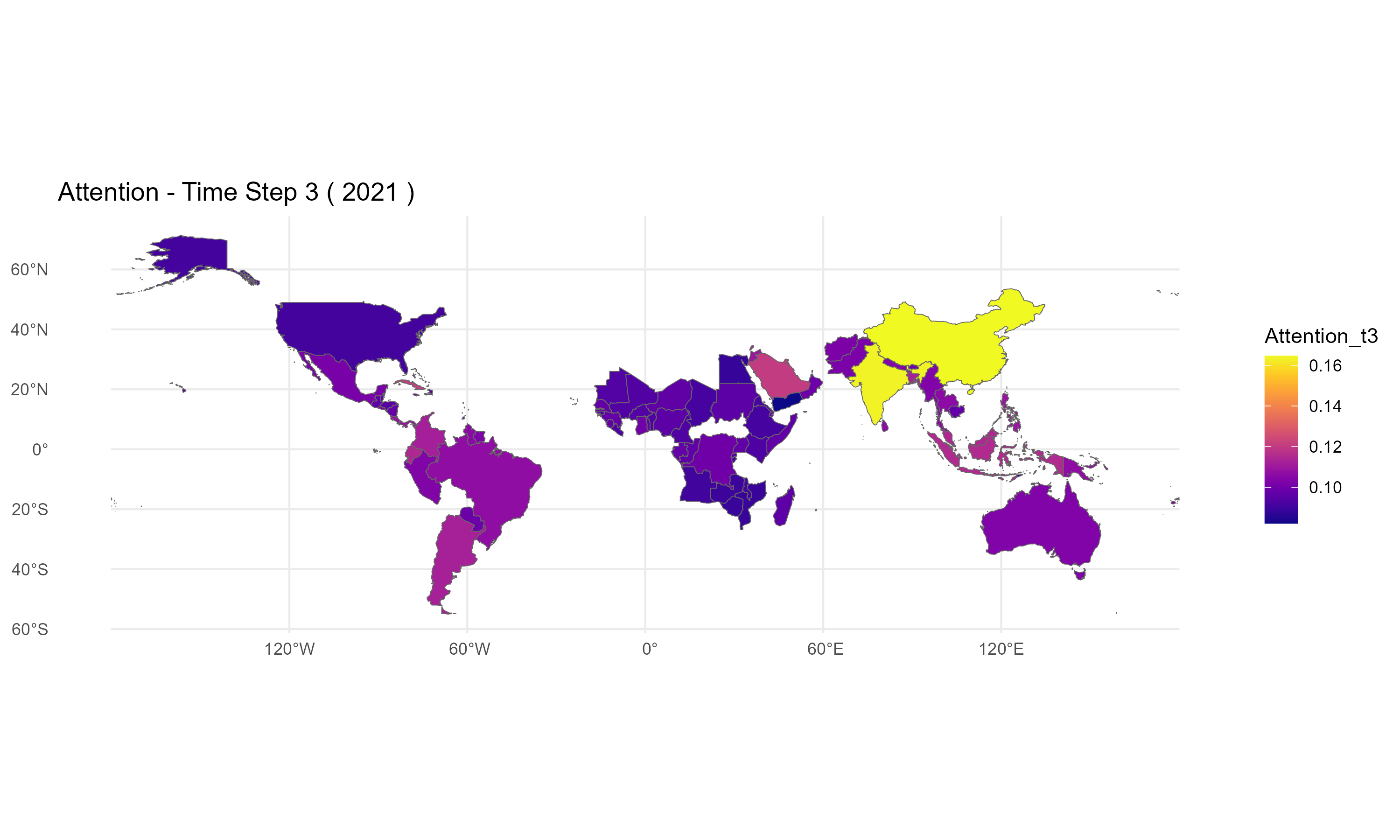

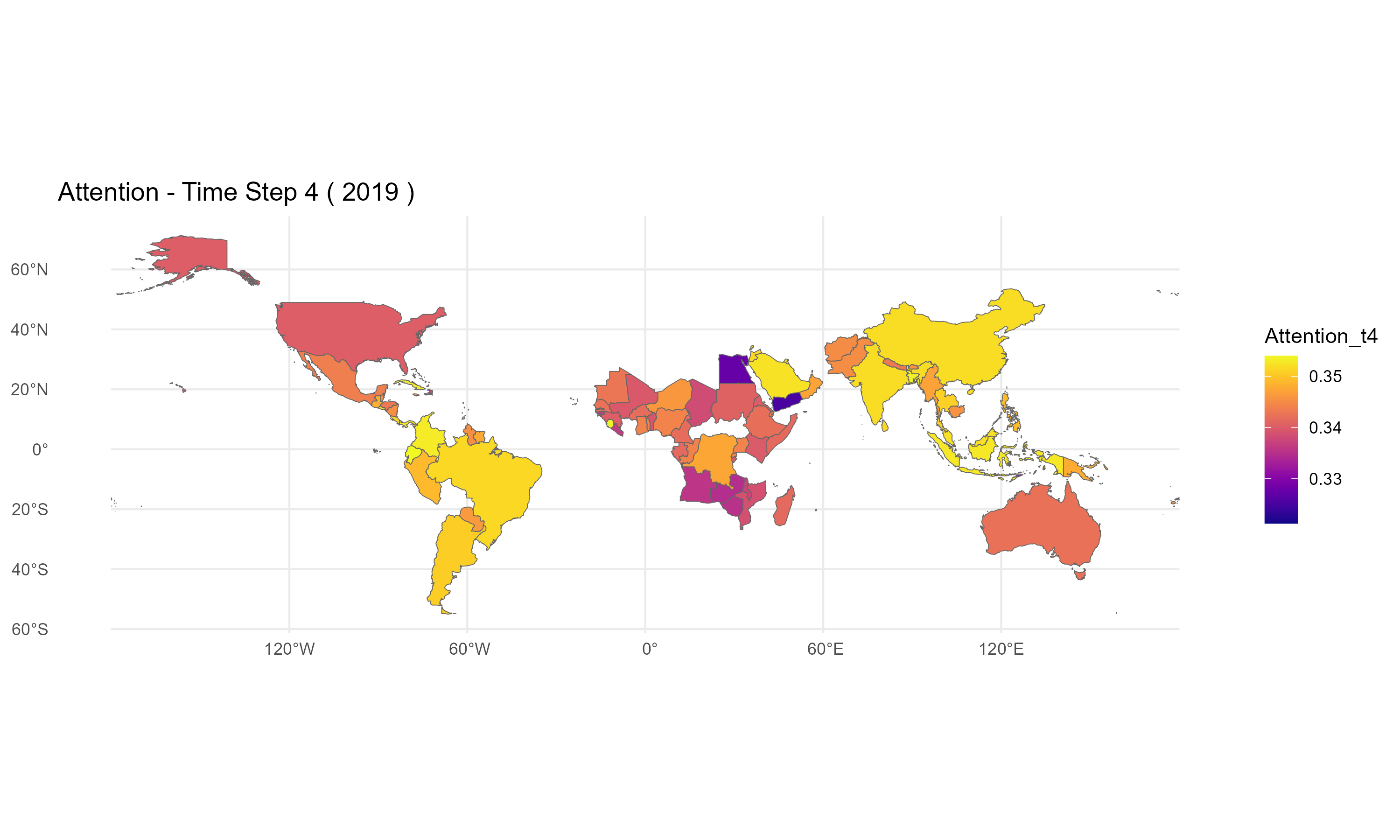

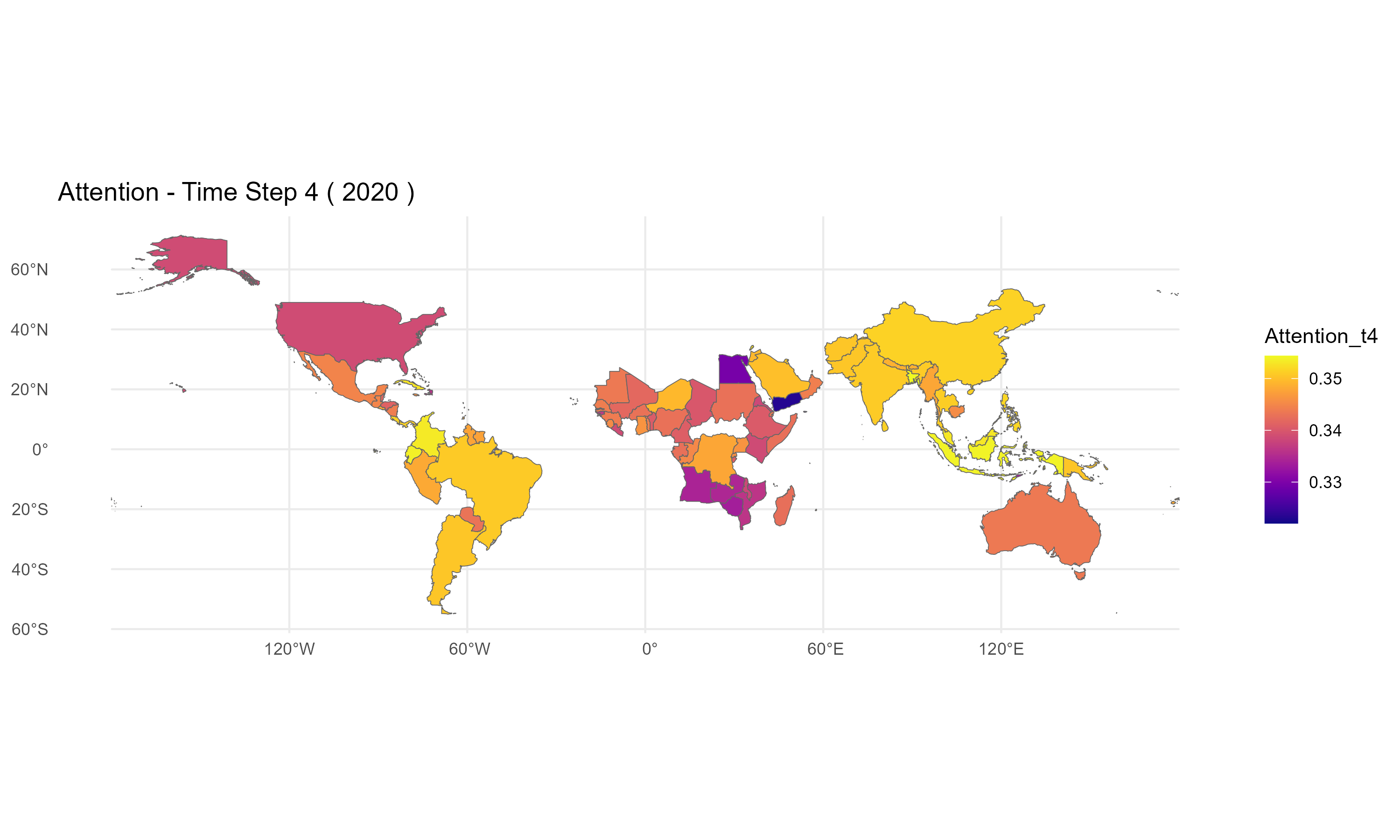

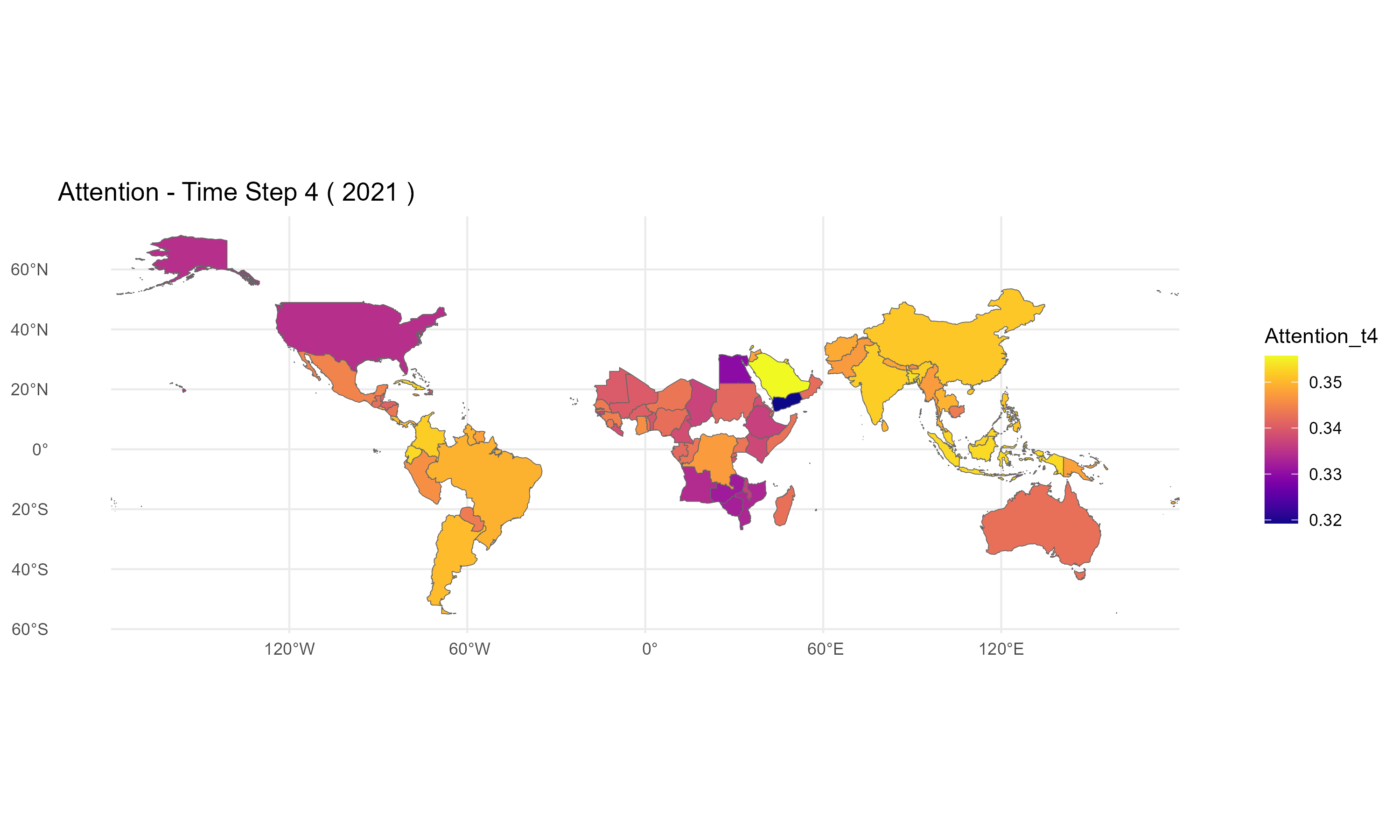

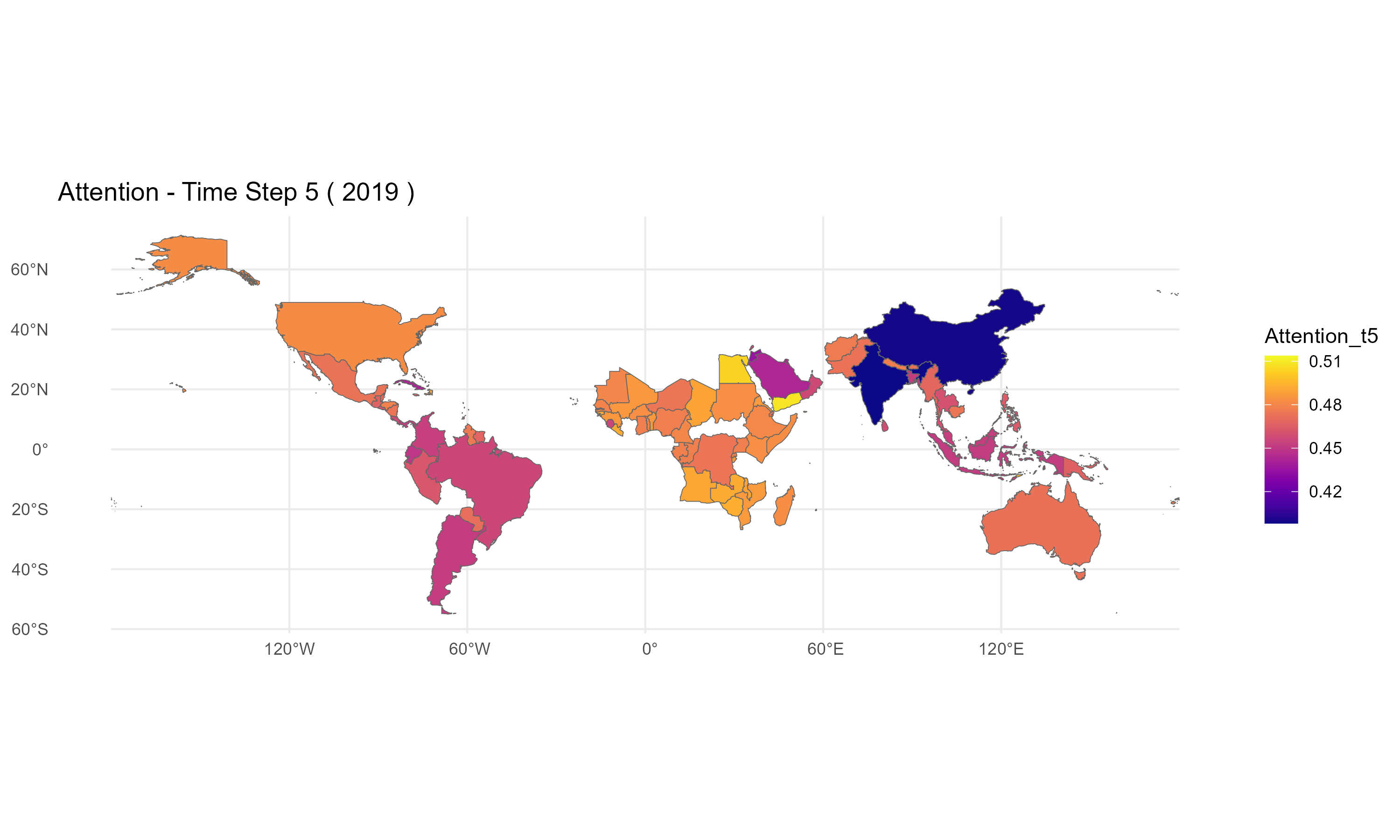

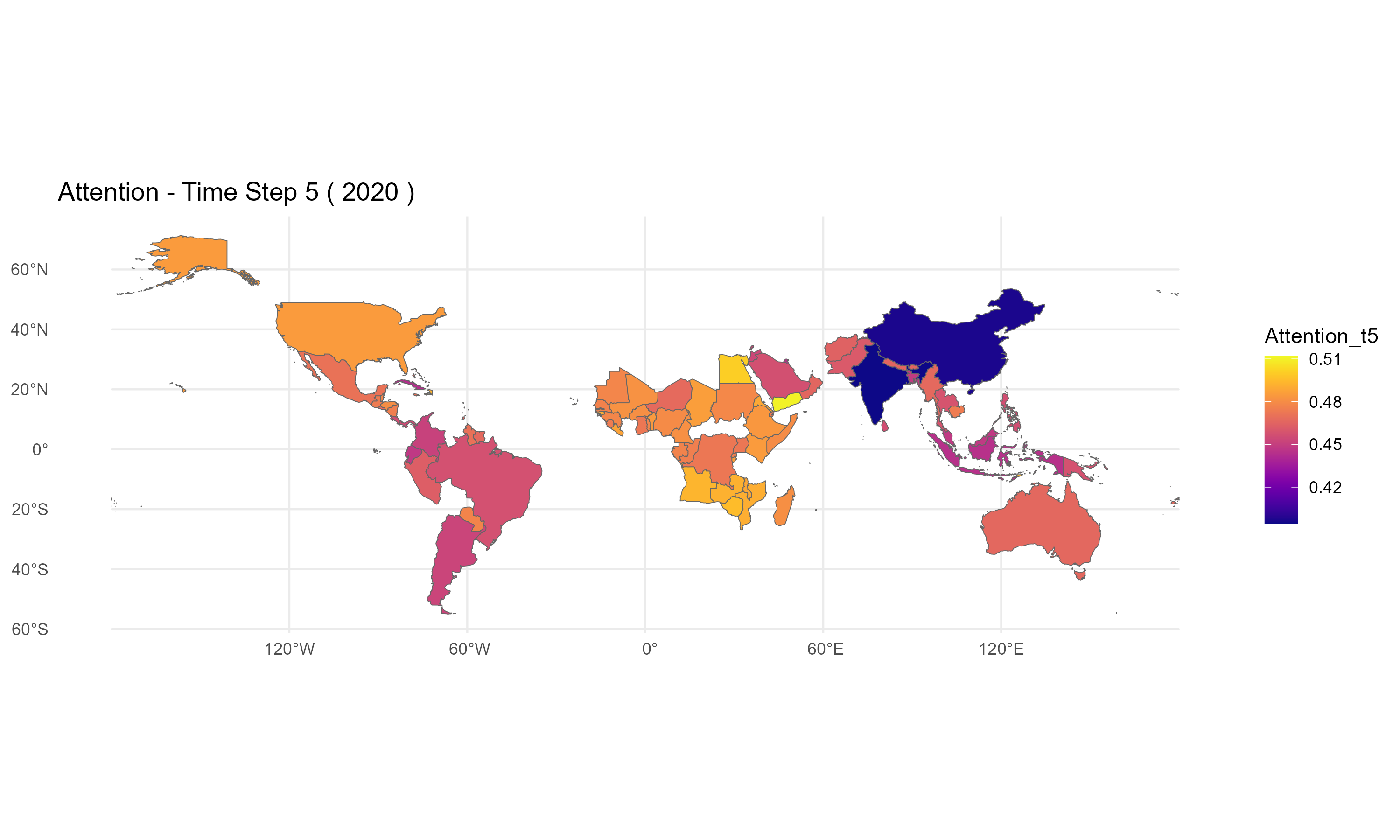

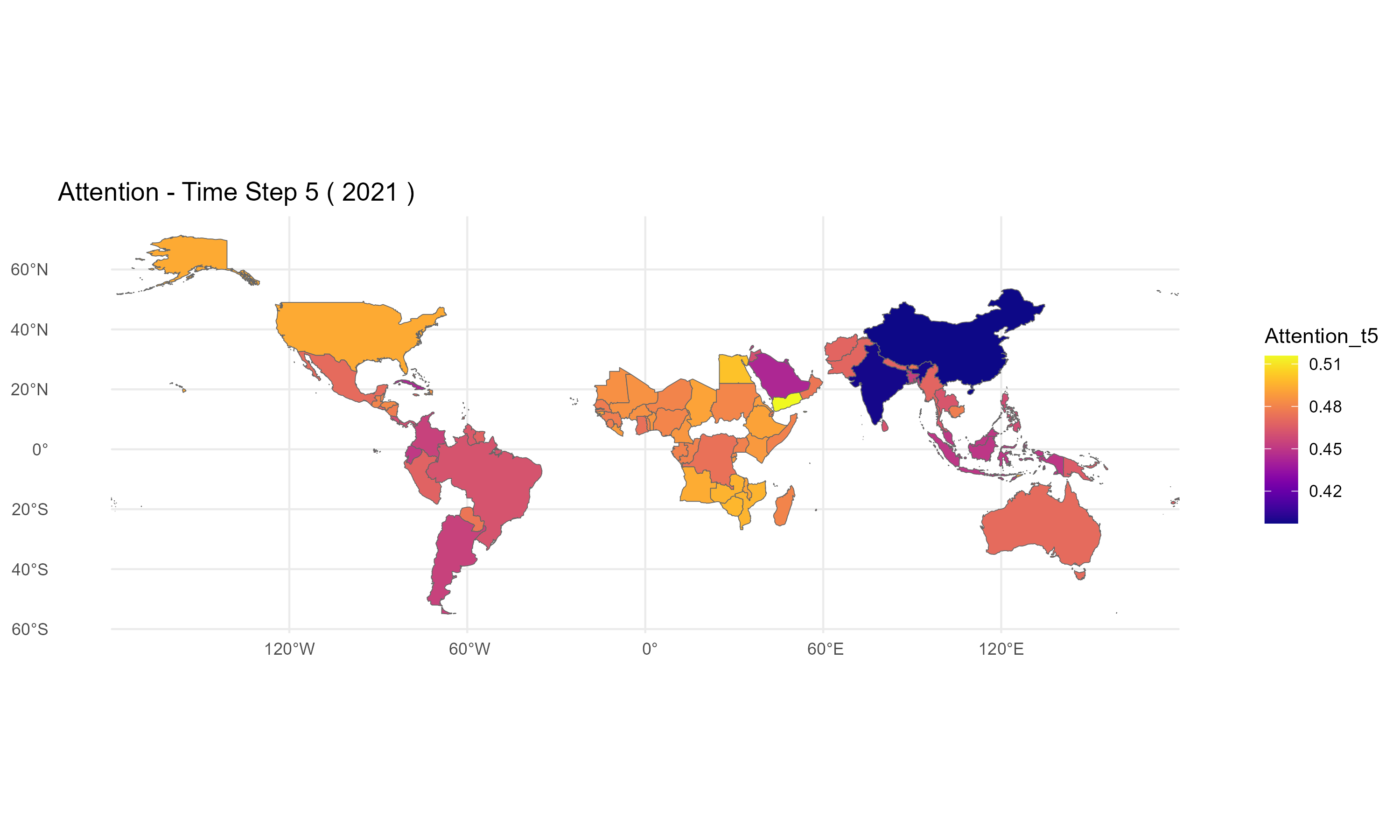

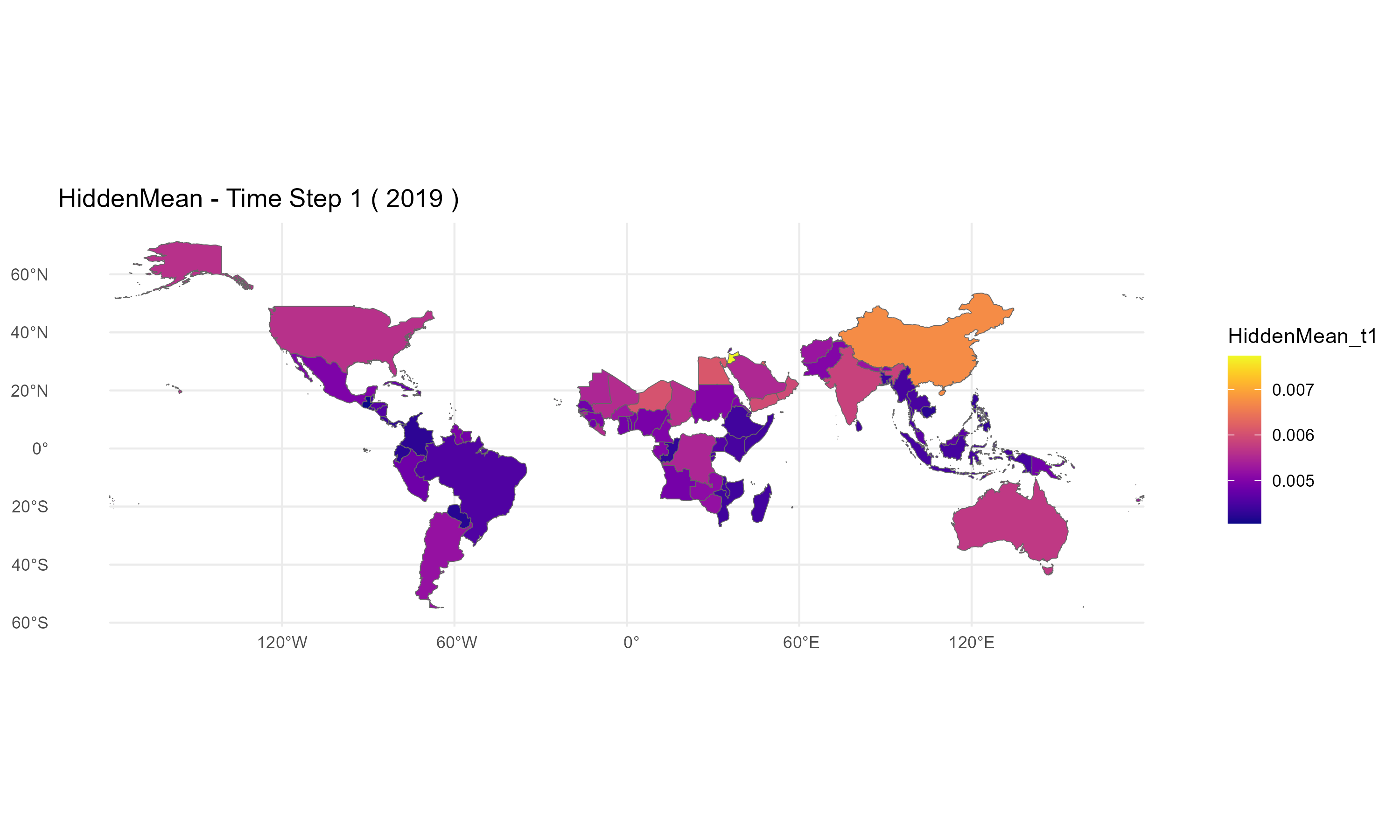

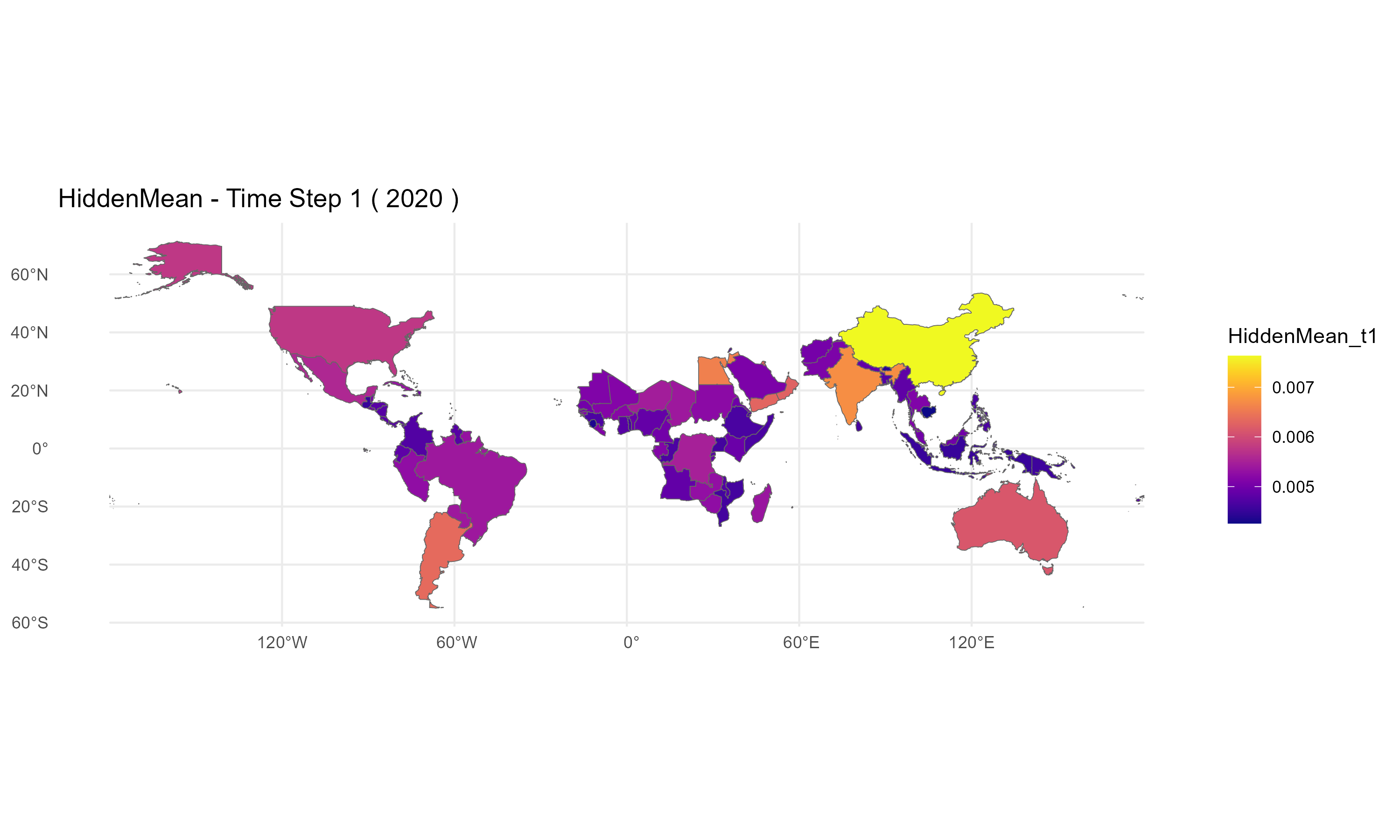

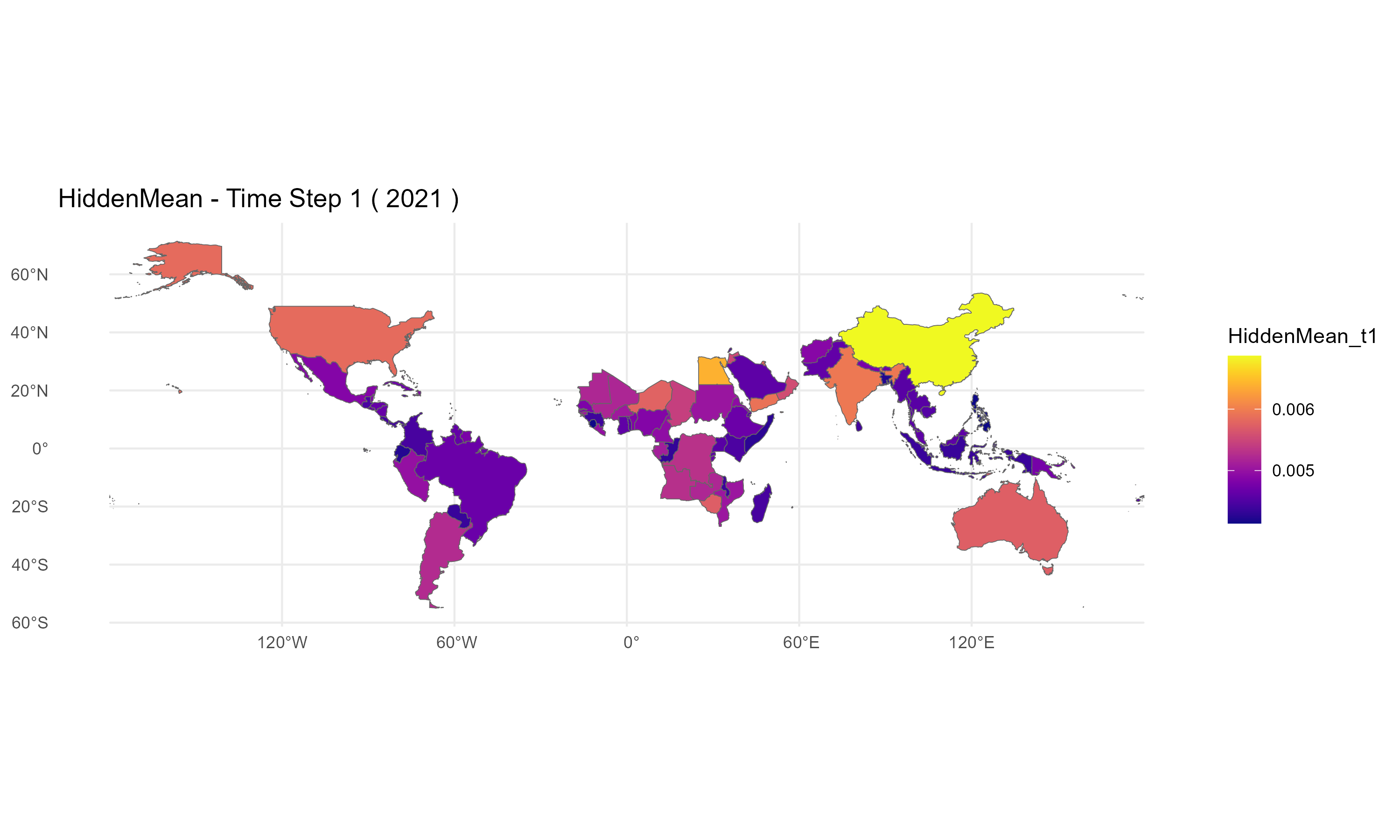

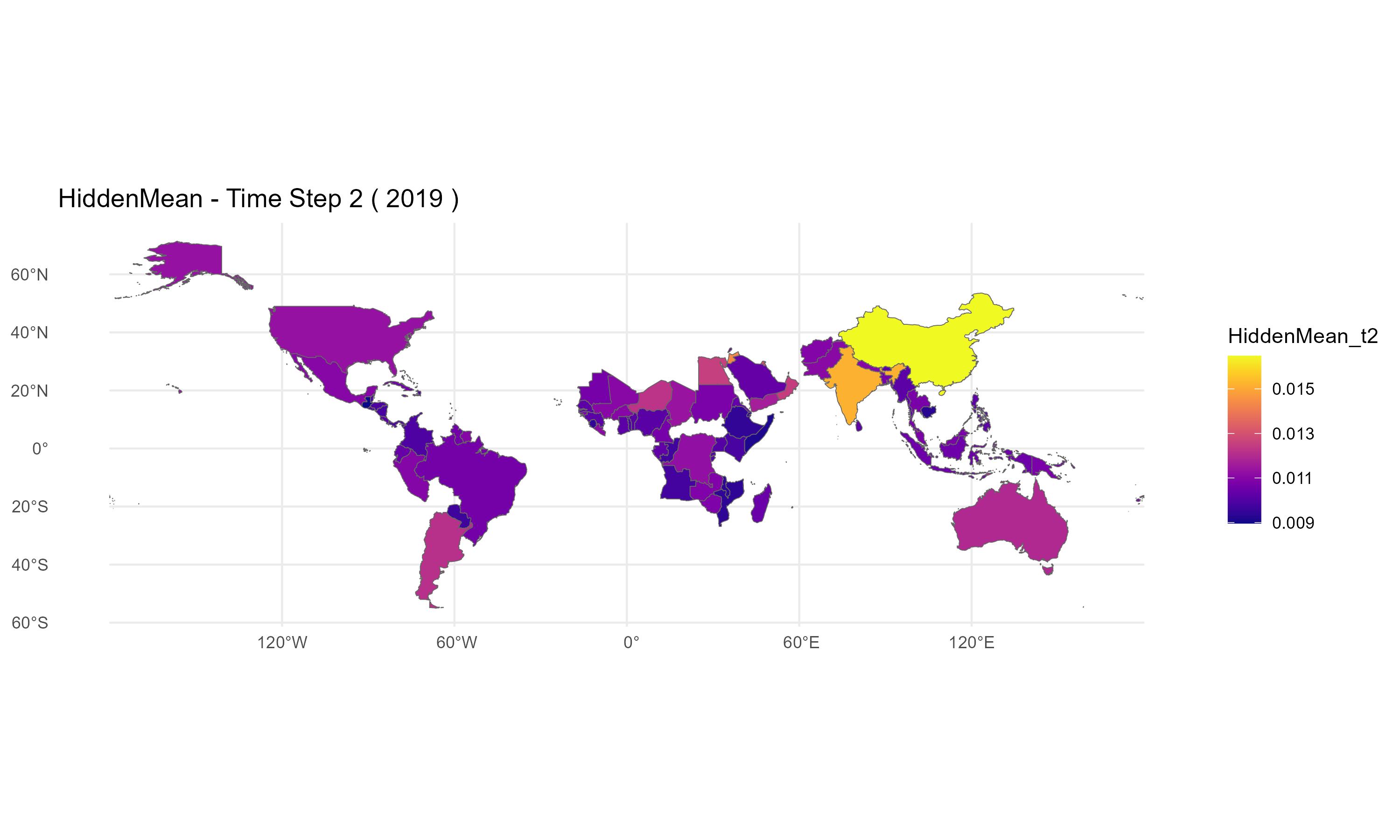

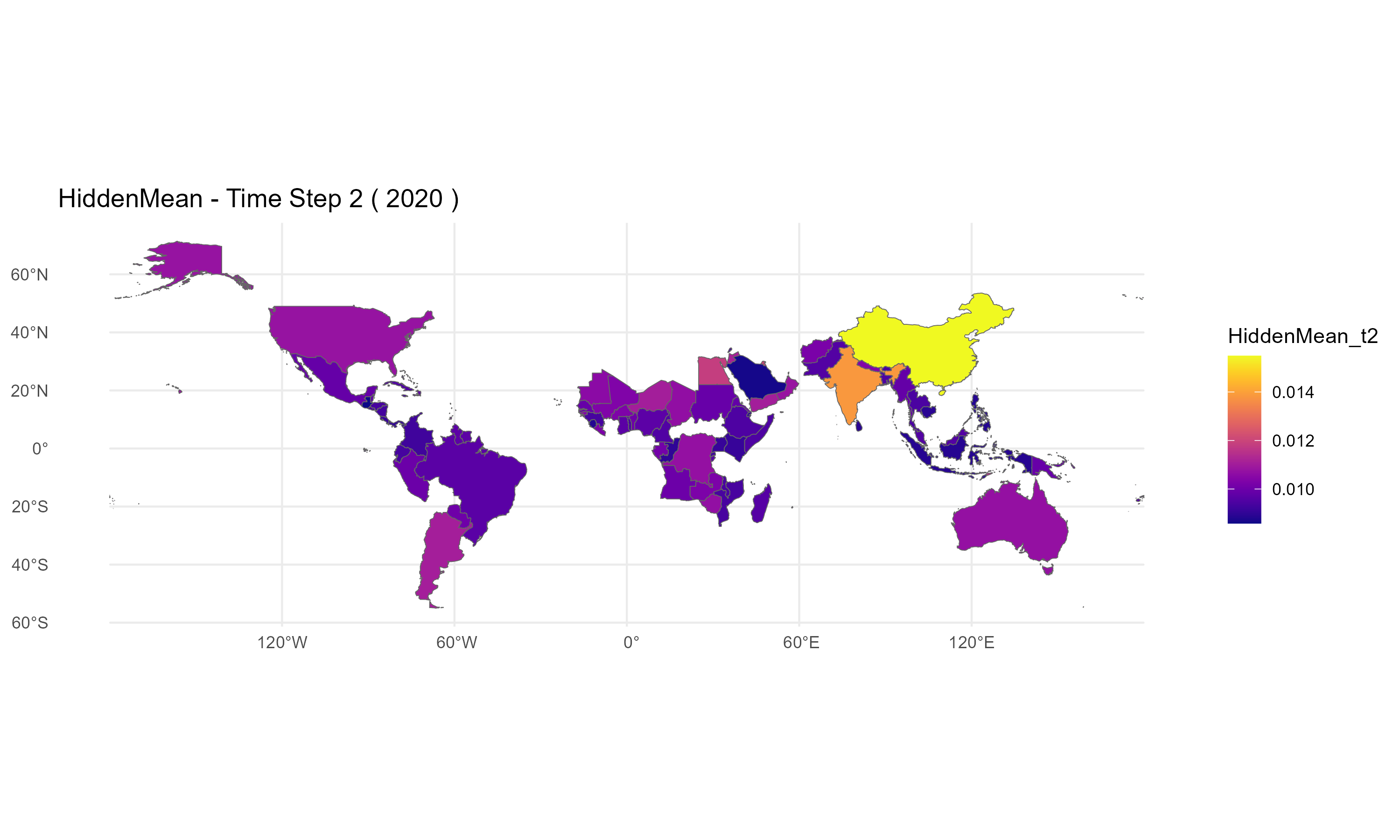

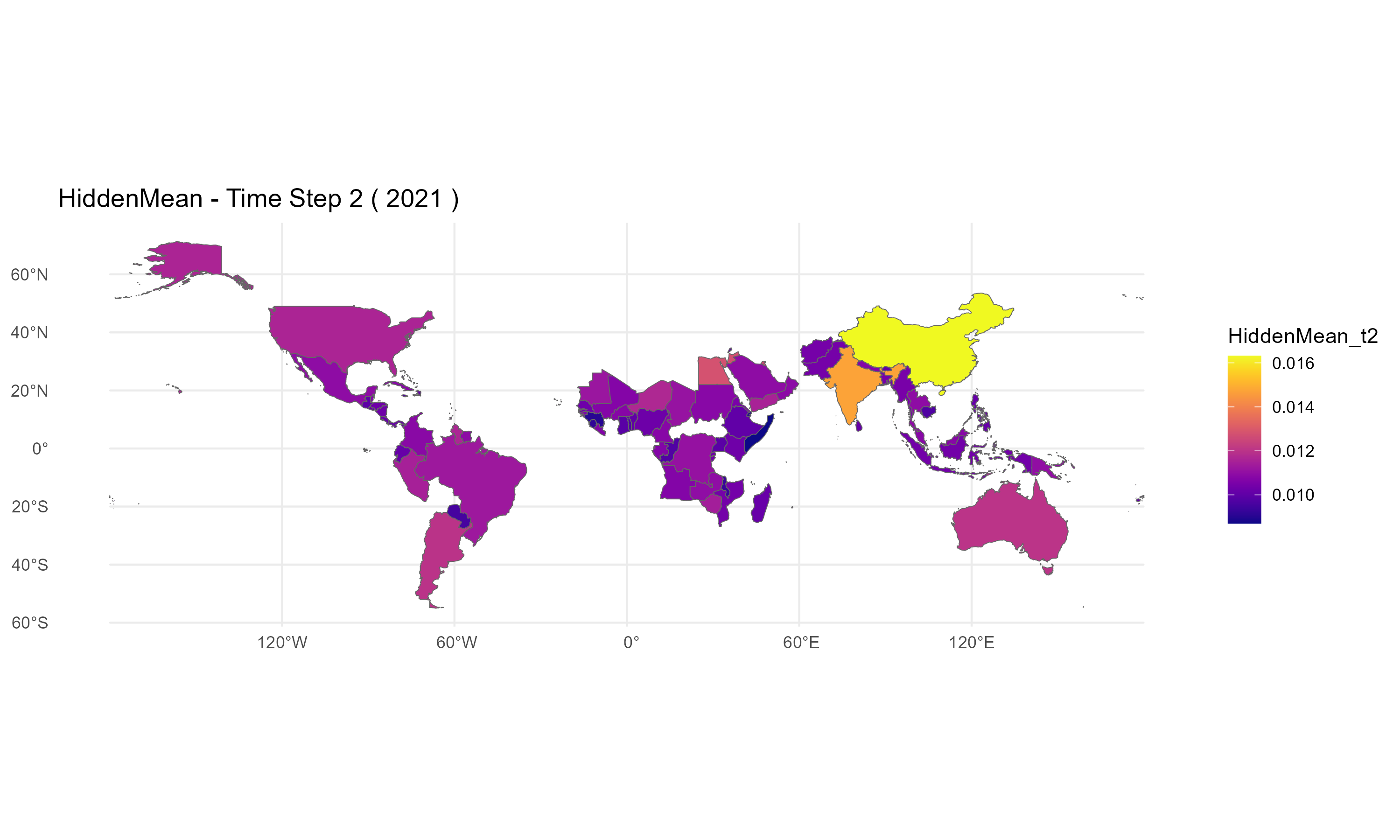

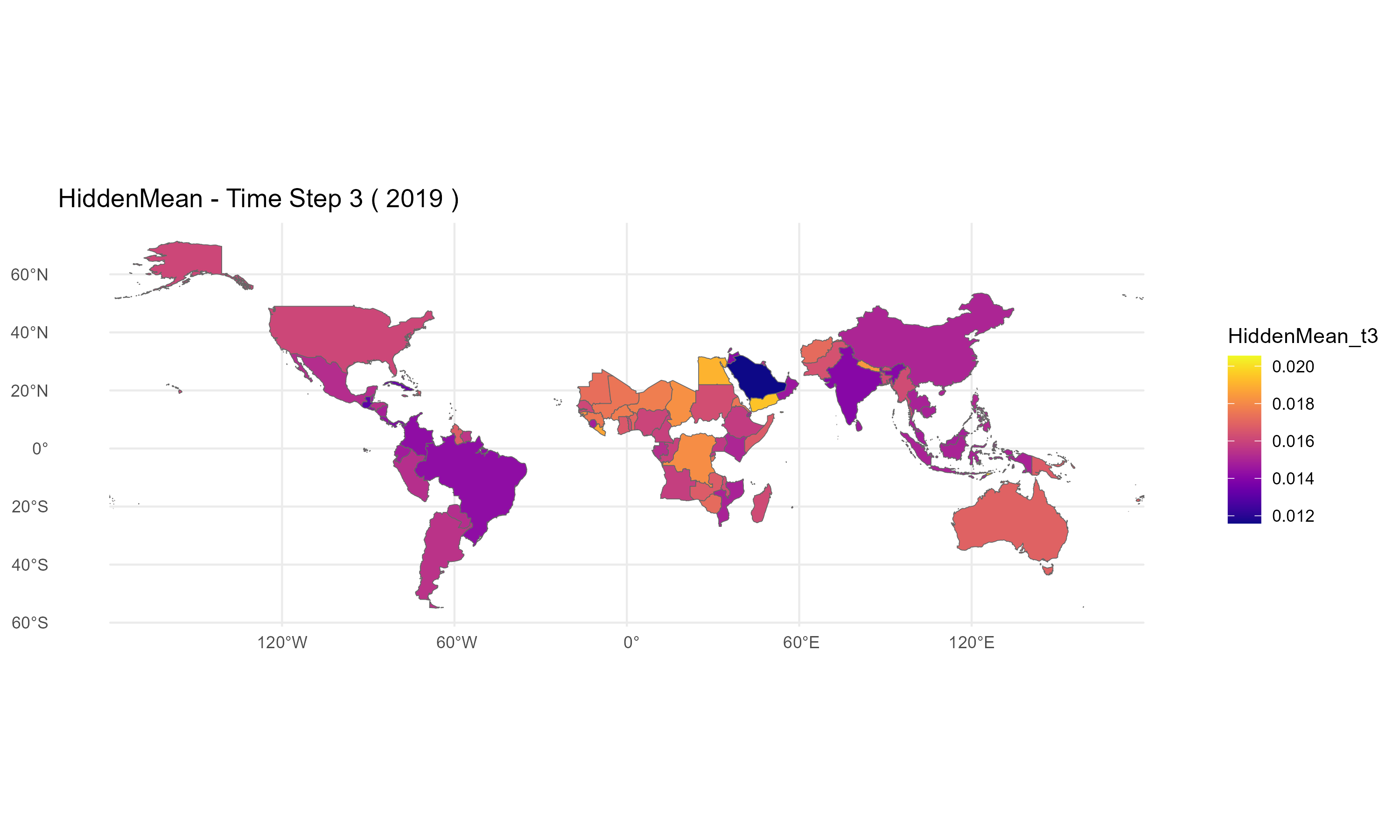

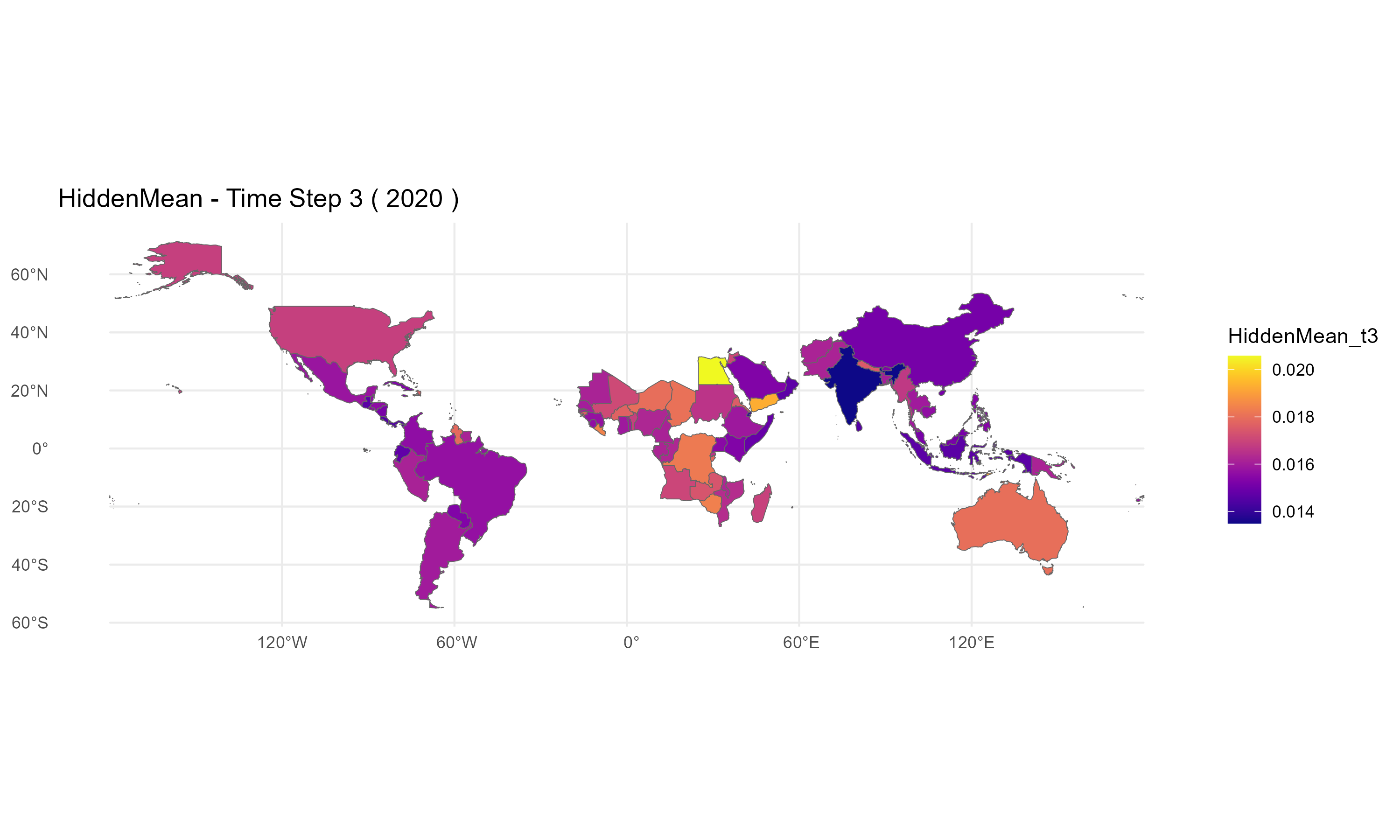

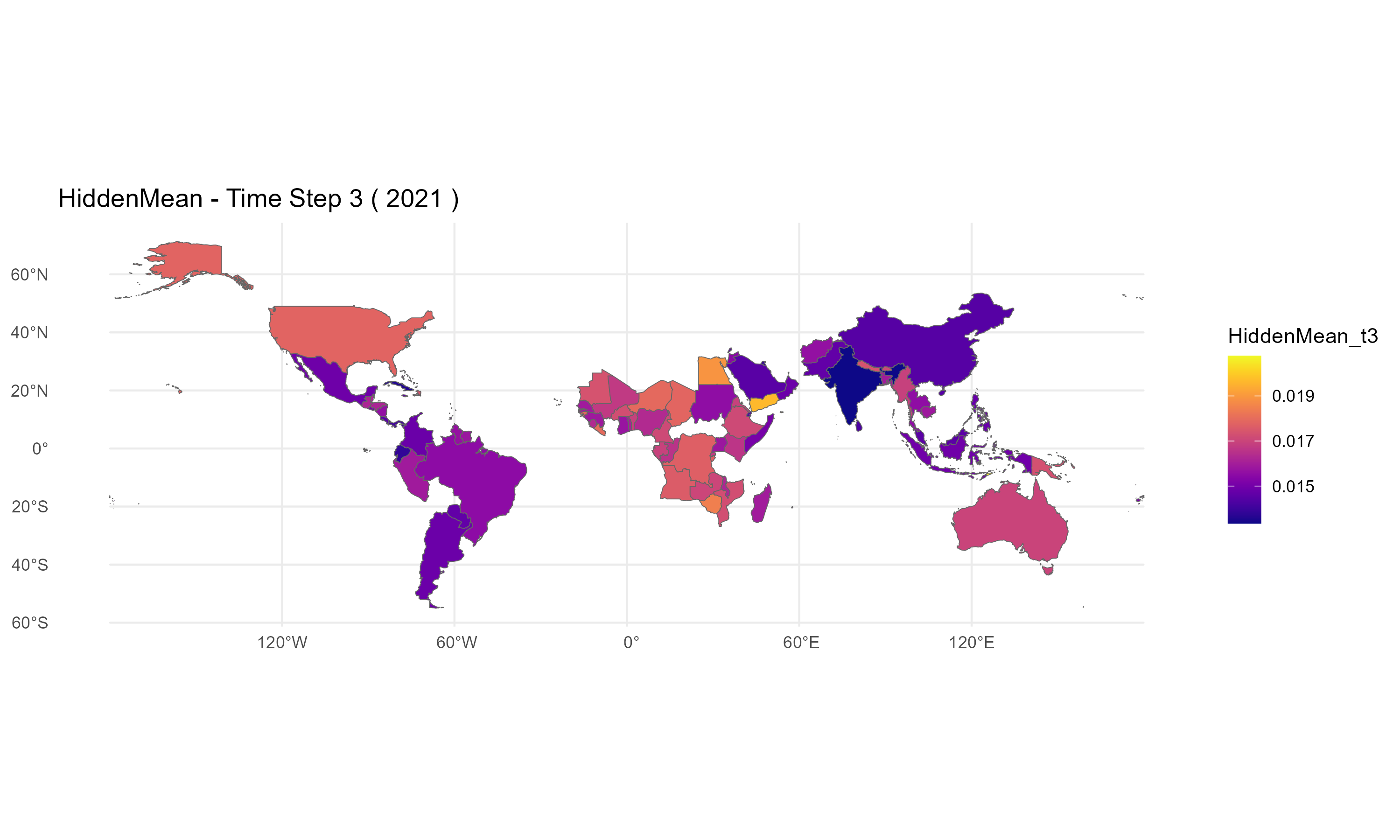

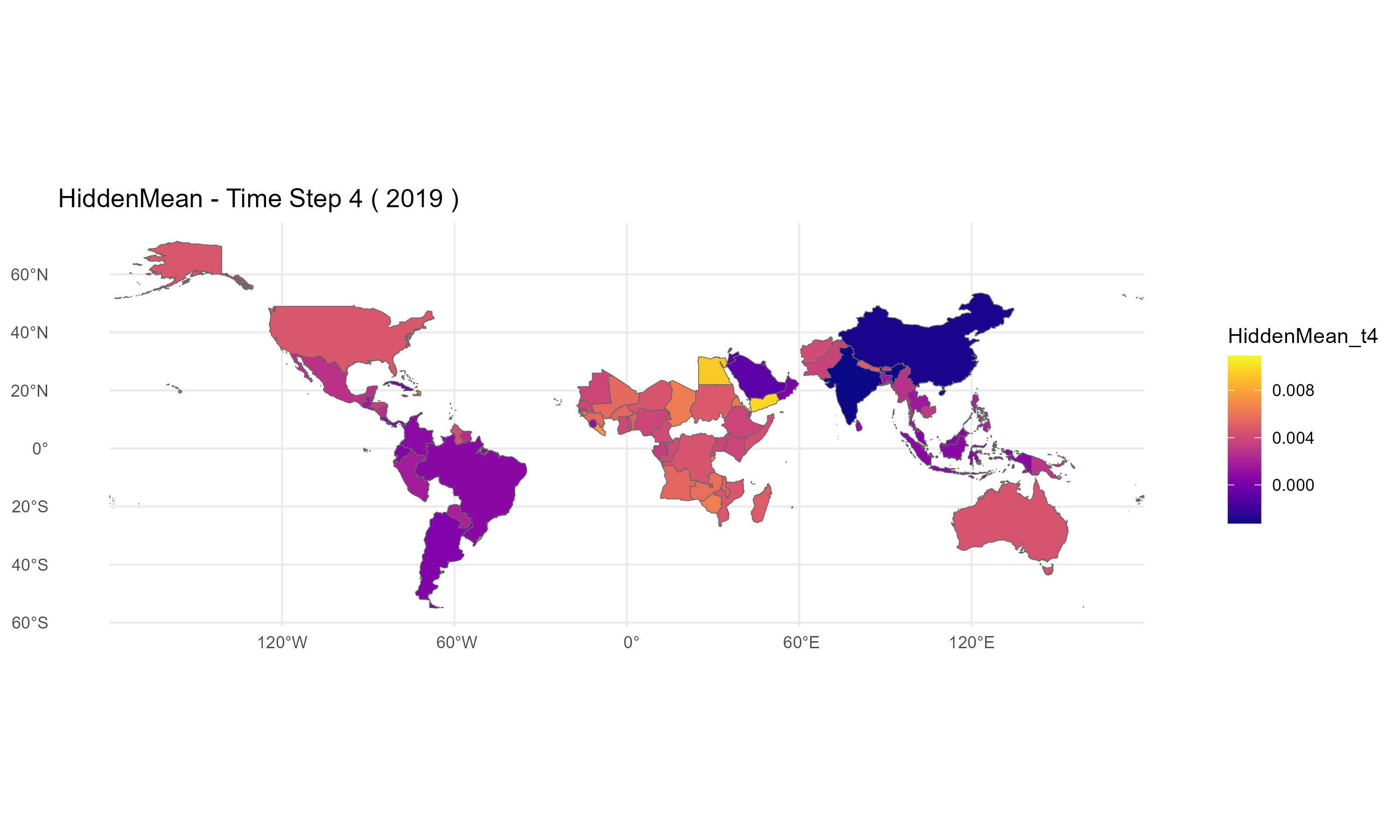

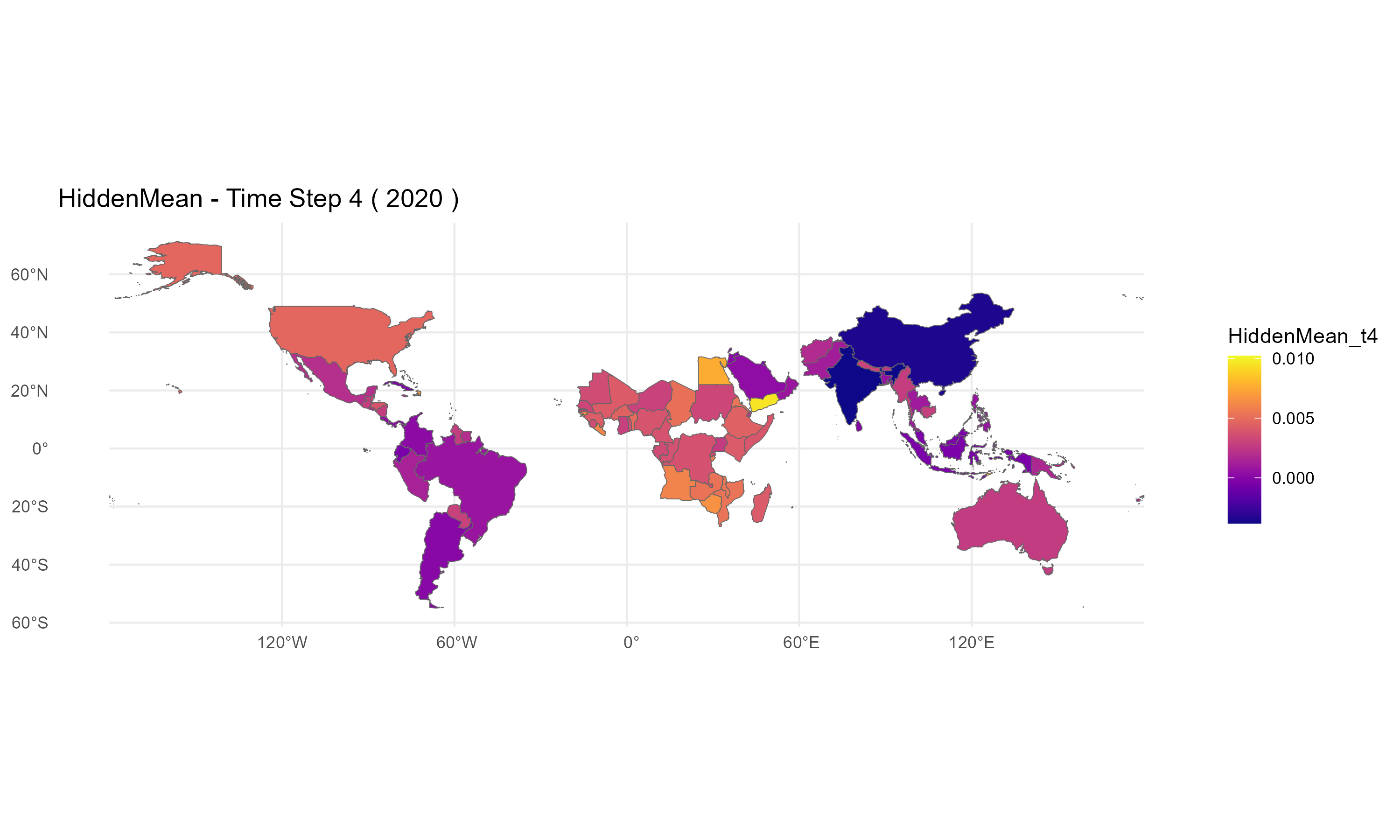

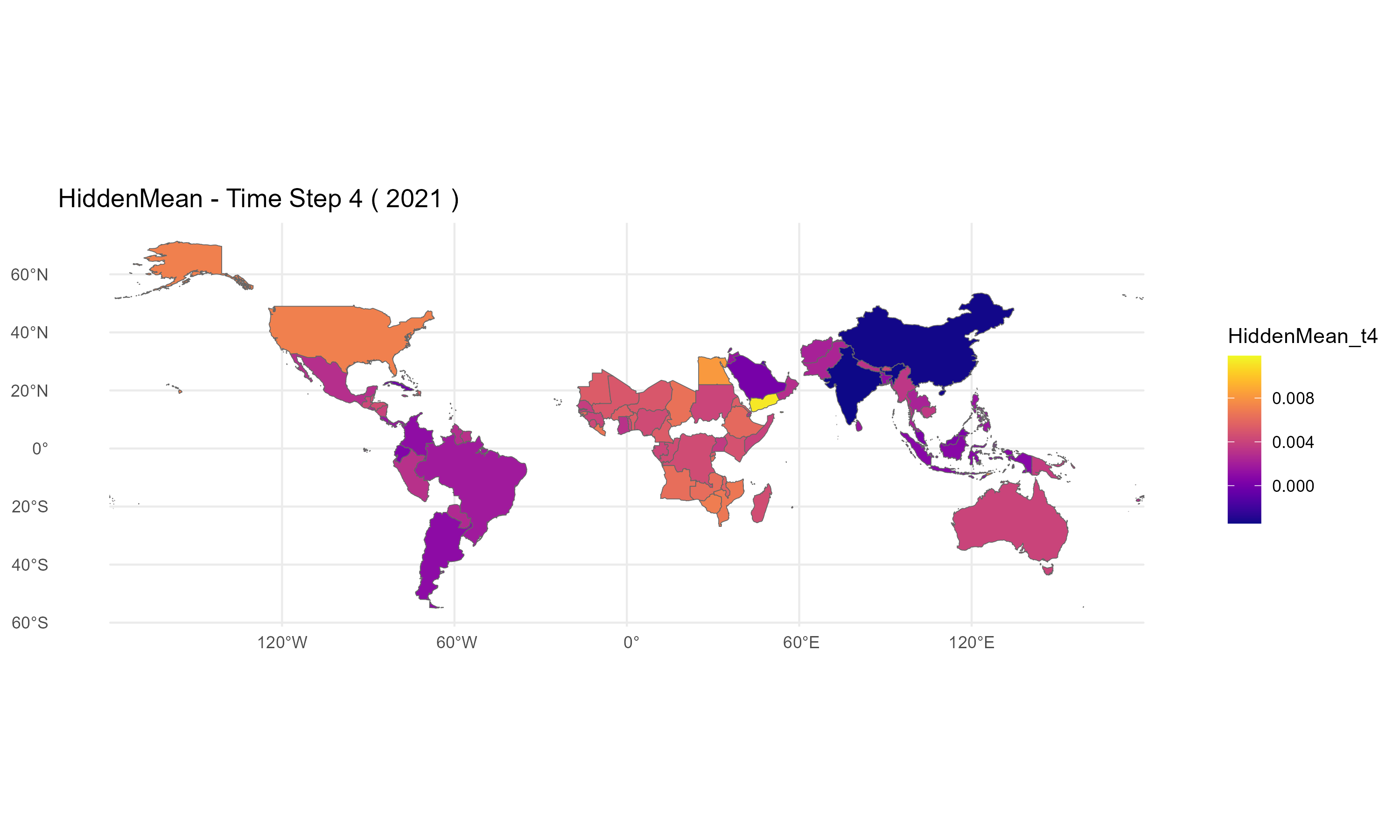

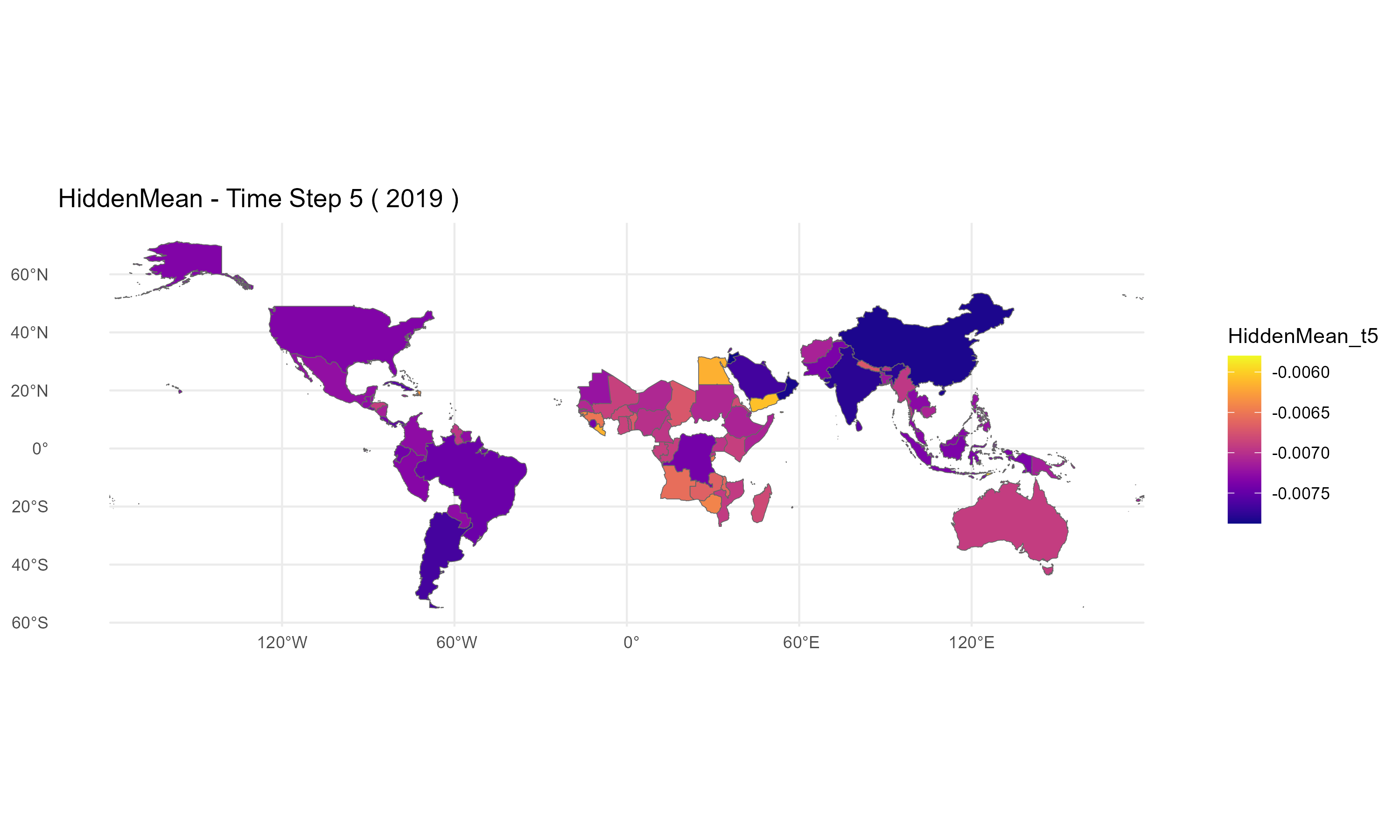

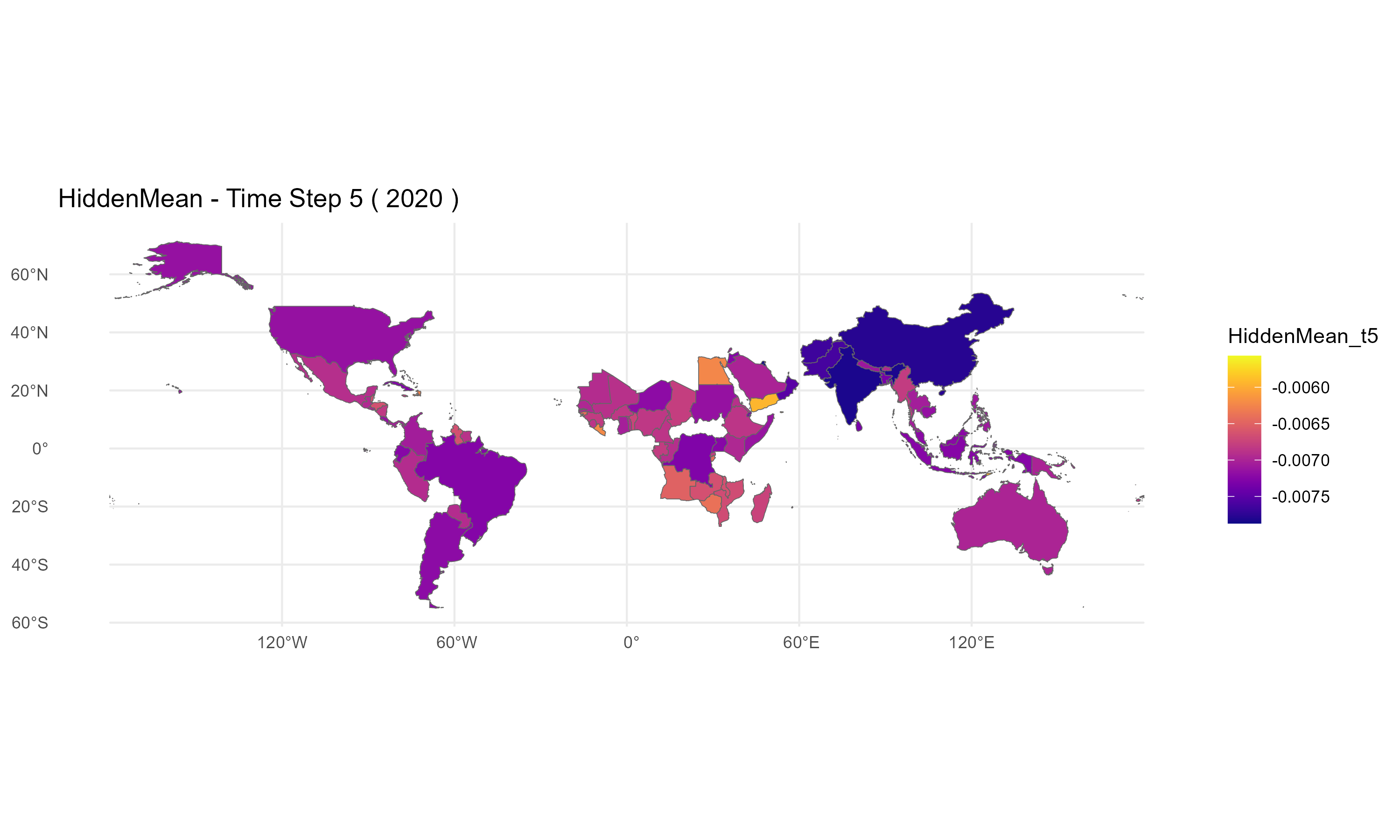

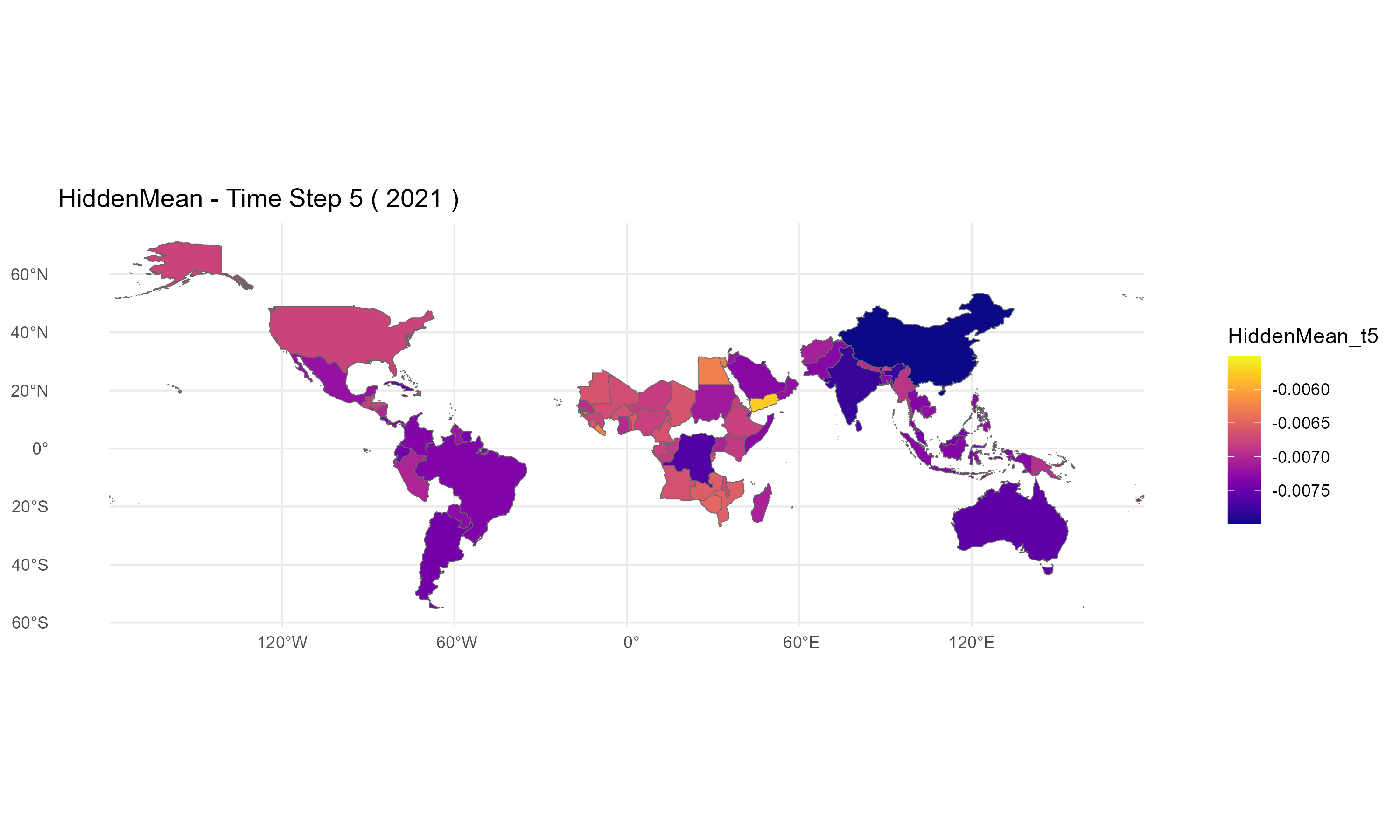


Figure S1. ConvLSTM Hidden States, Activation, and Attention Weights for all Countries (2019–2021)


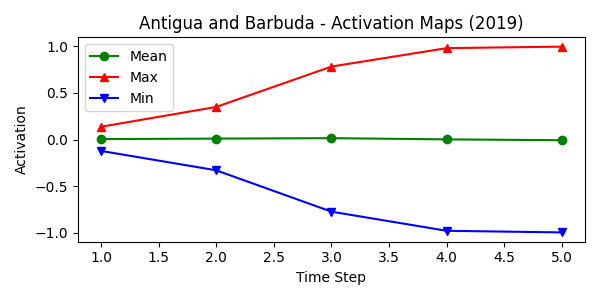

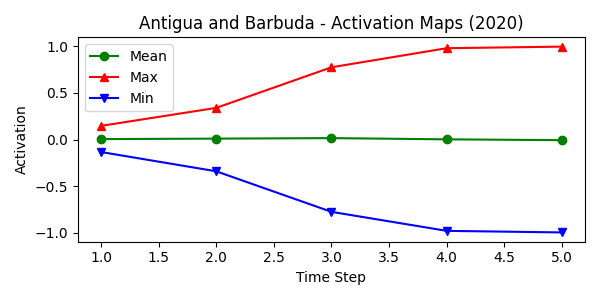

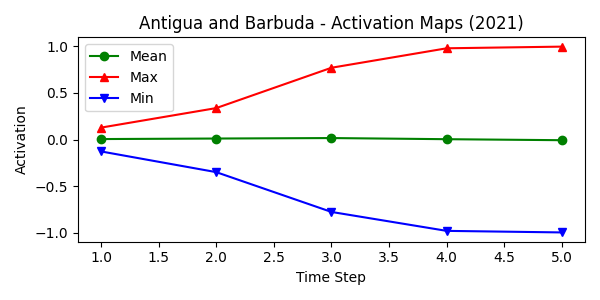

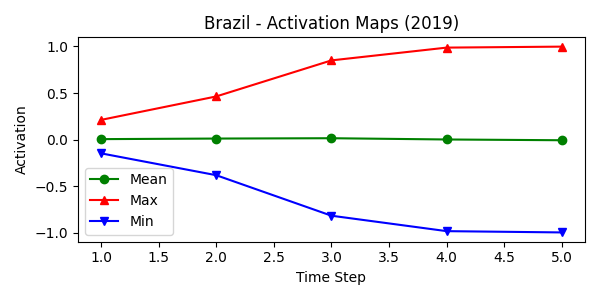

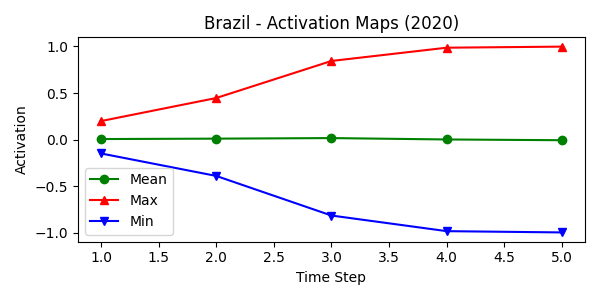

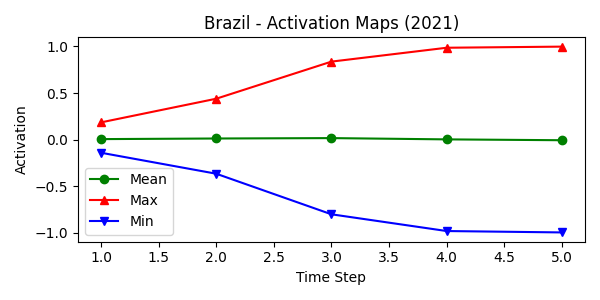

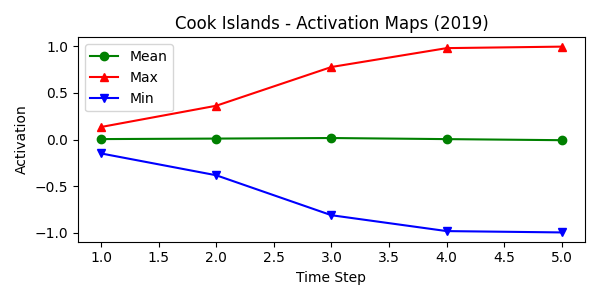

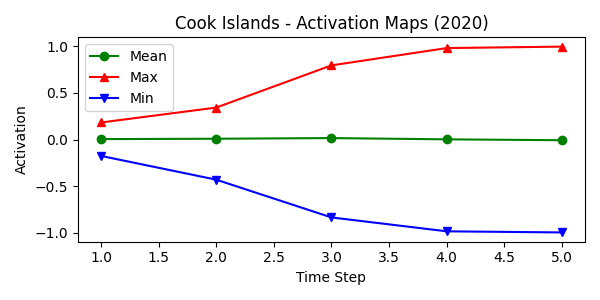

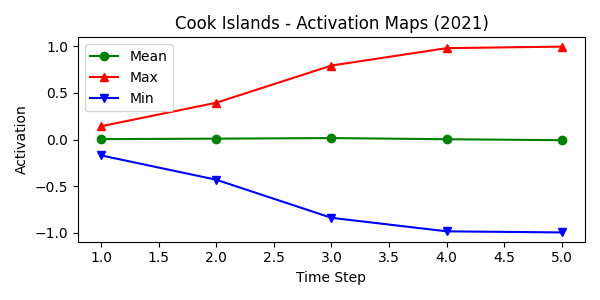

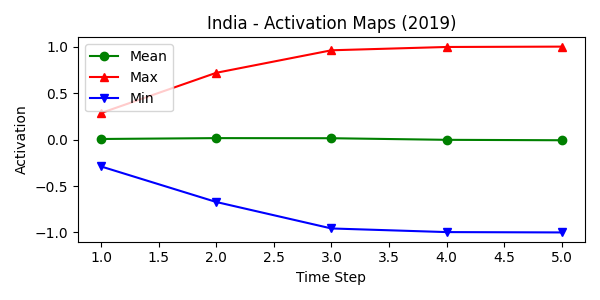

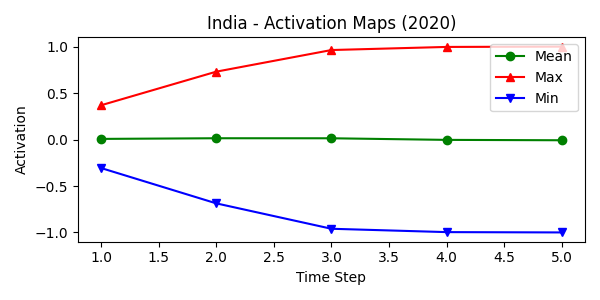

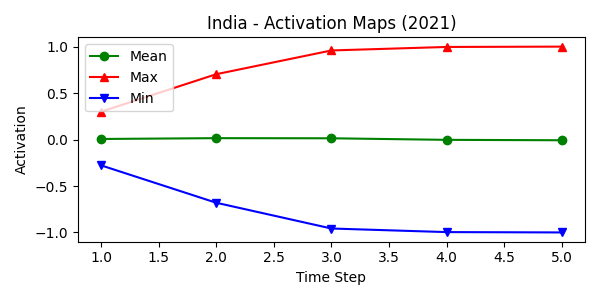

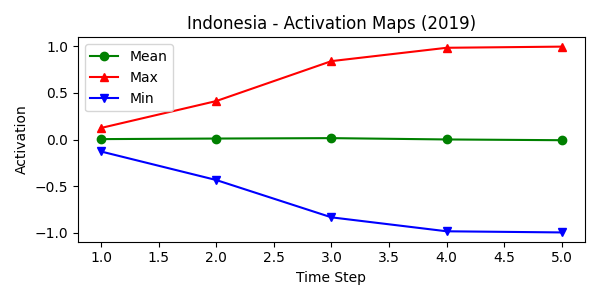

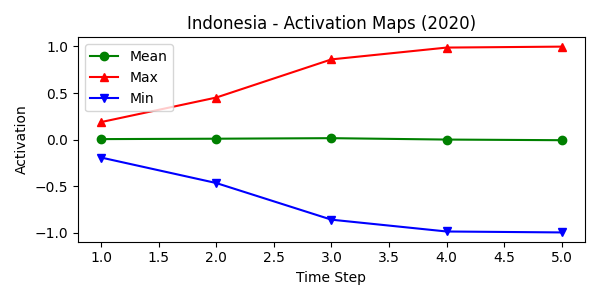

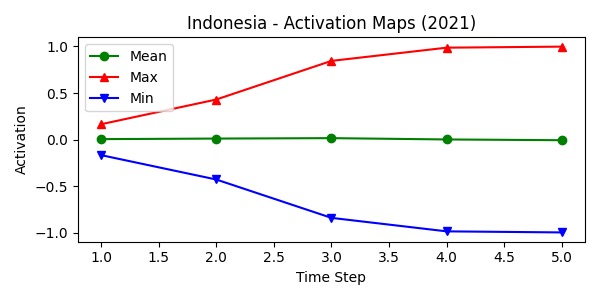

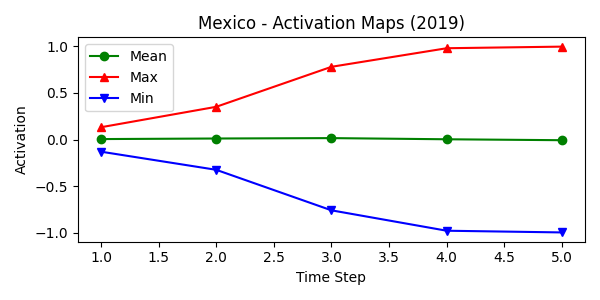

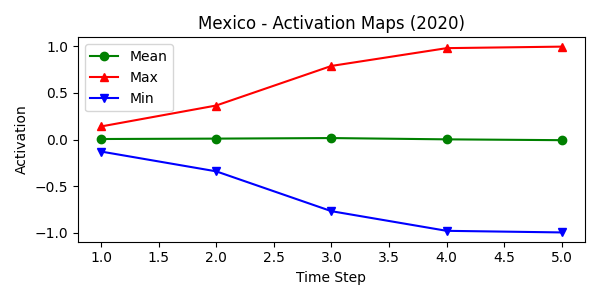

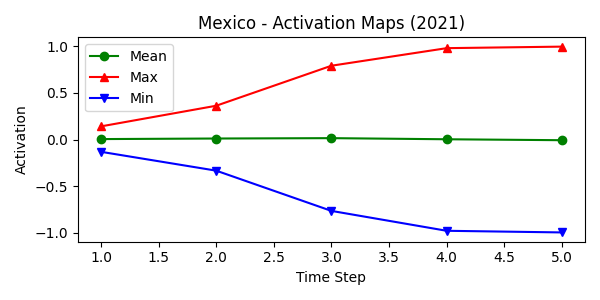

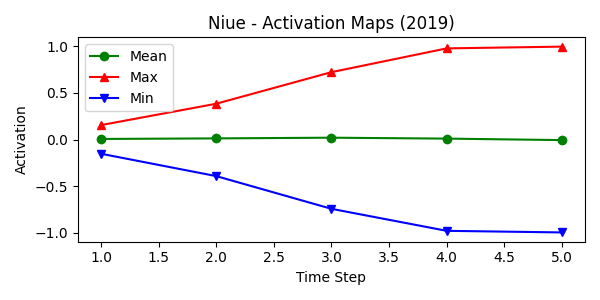

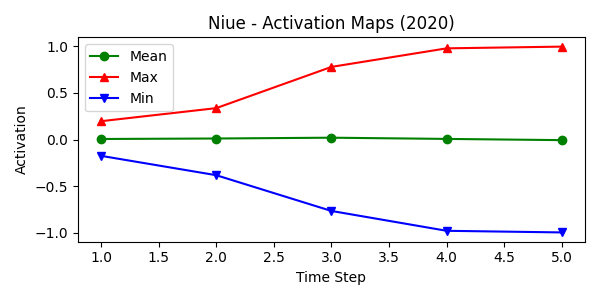

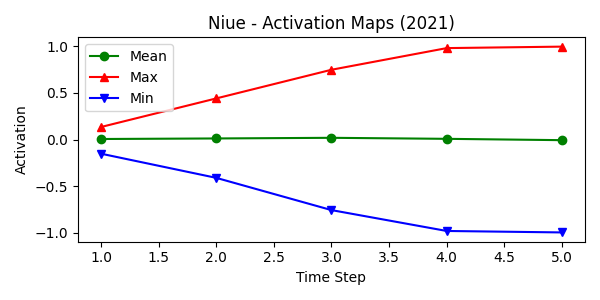

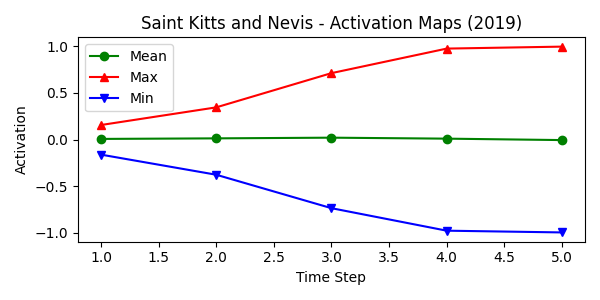

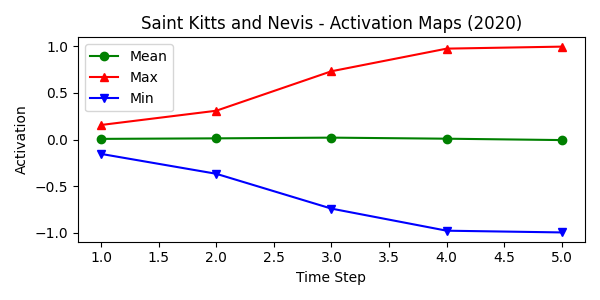

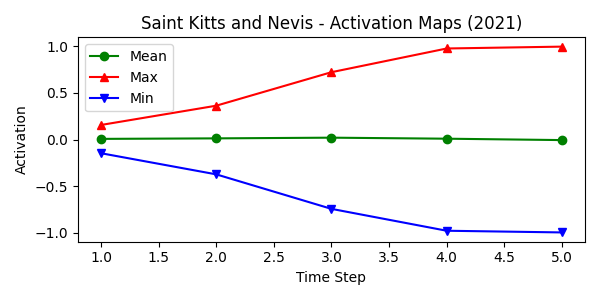

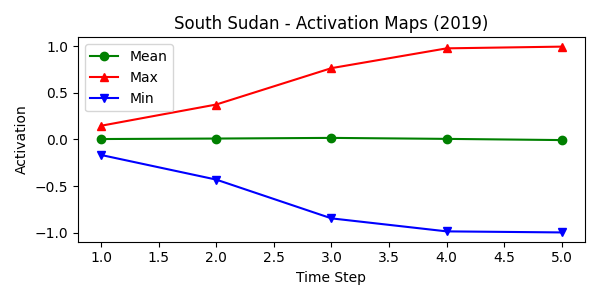

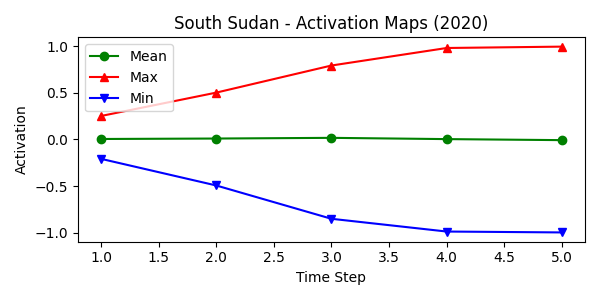

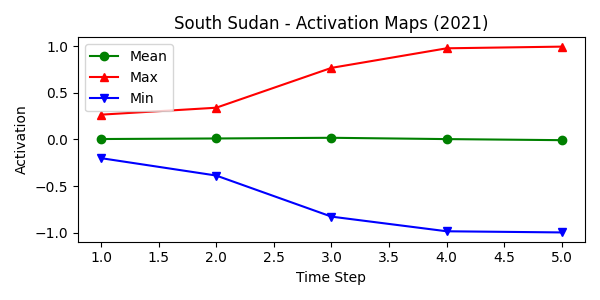

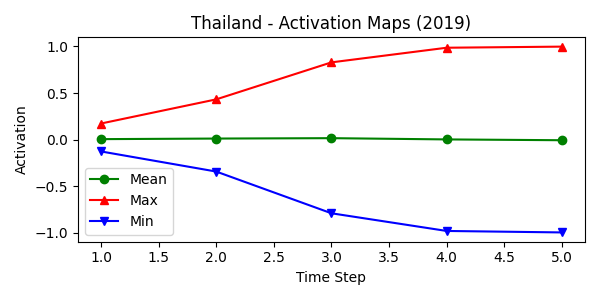

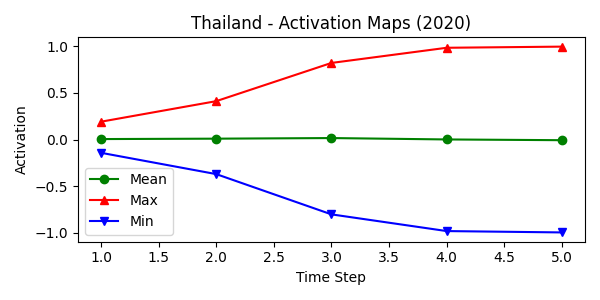

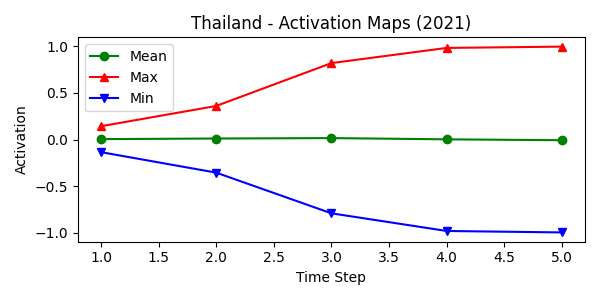

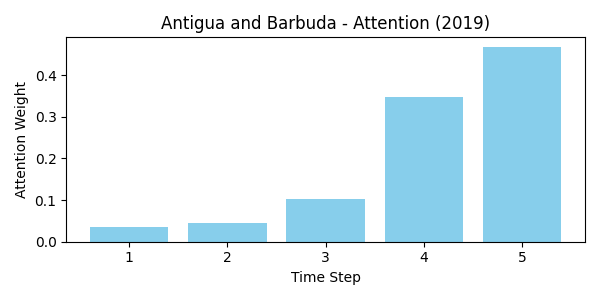

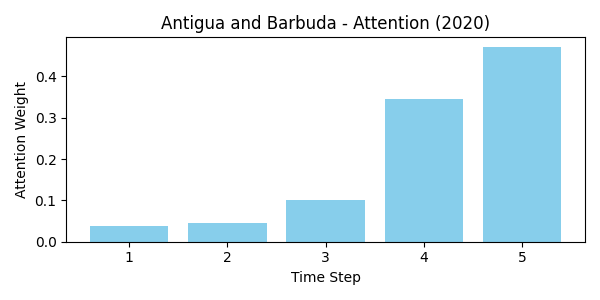

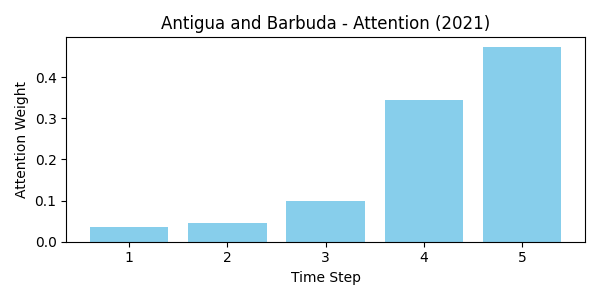

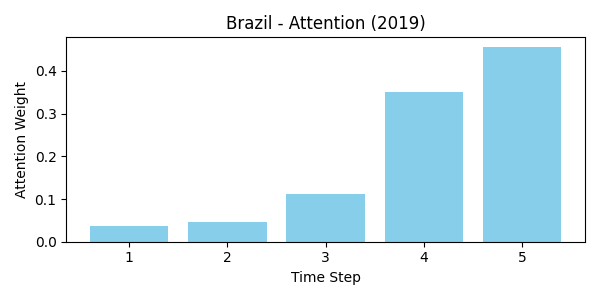

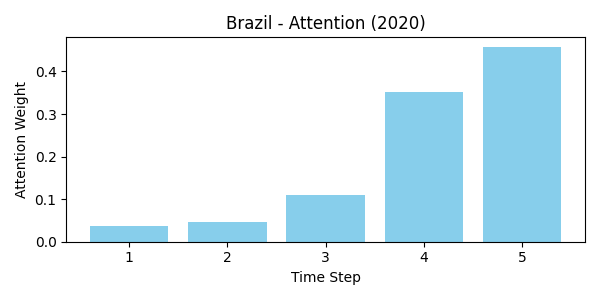

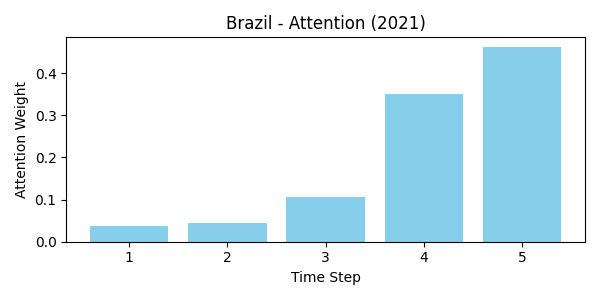

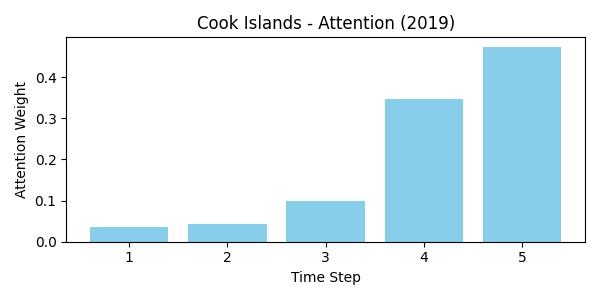

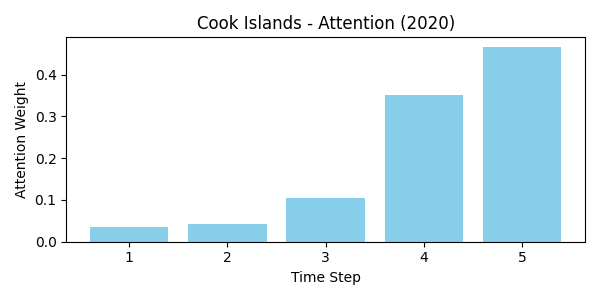

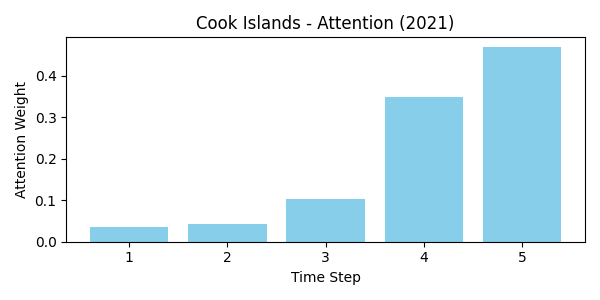

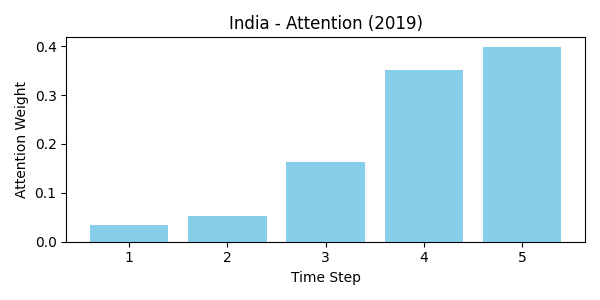

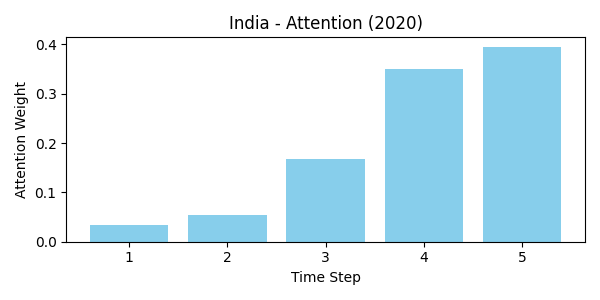

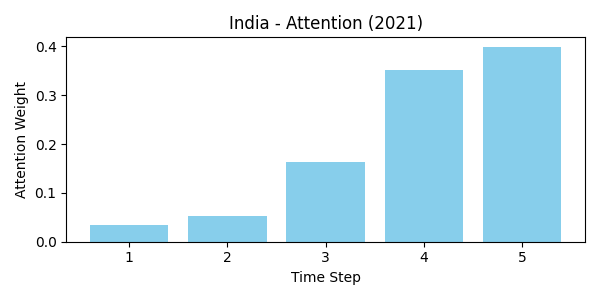

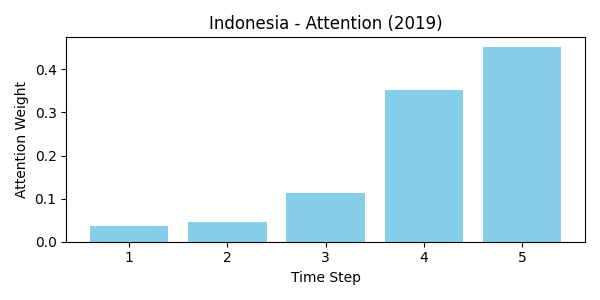

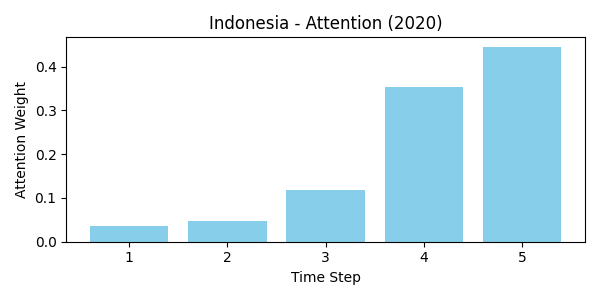

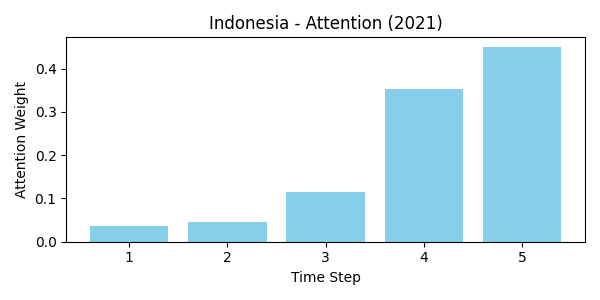

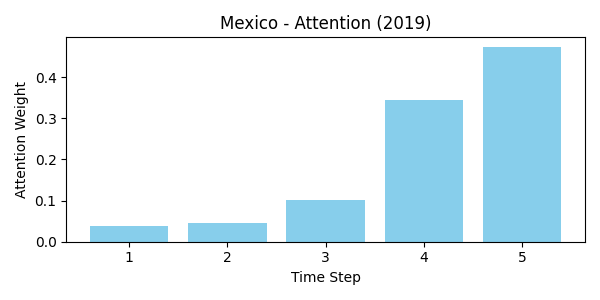

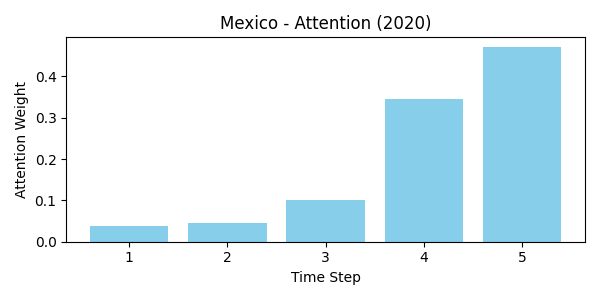

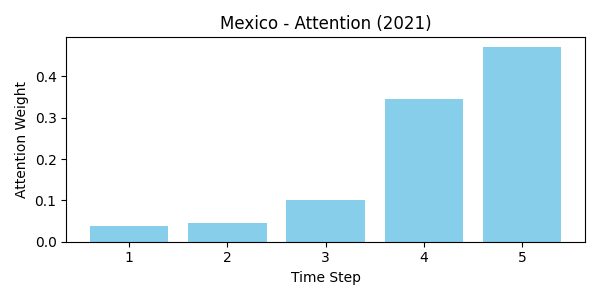

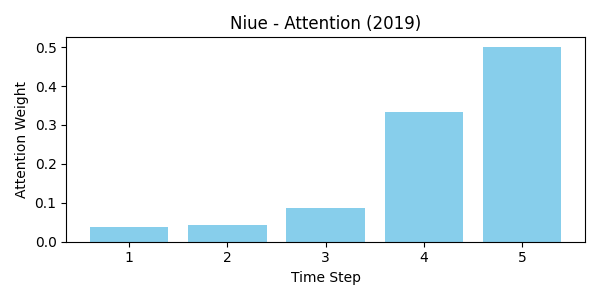

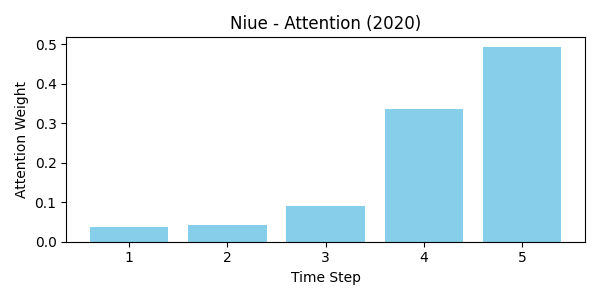

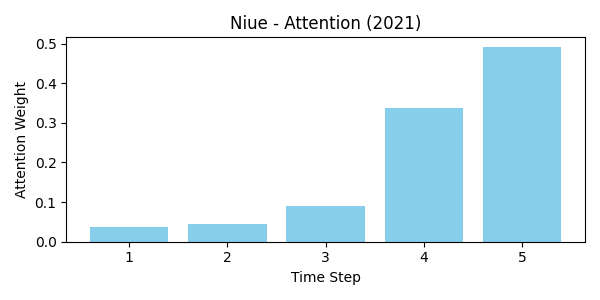

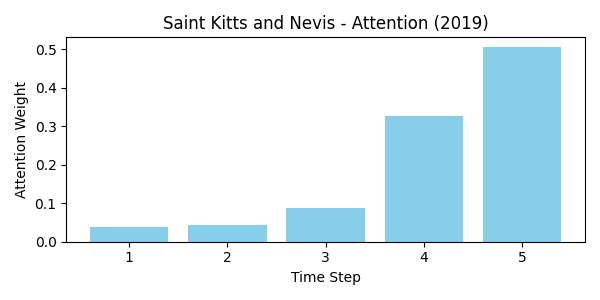

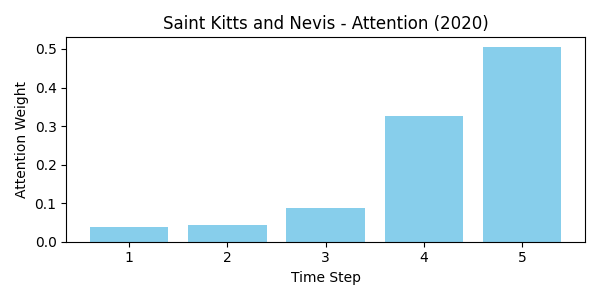

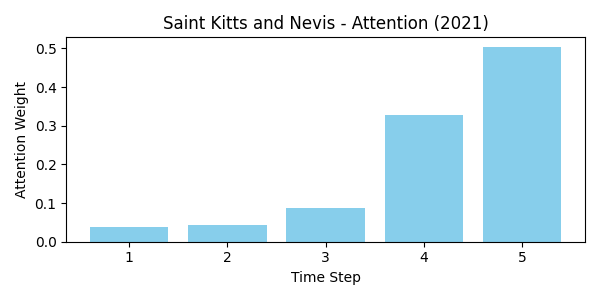

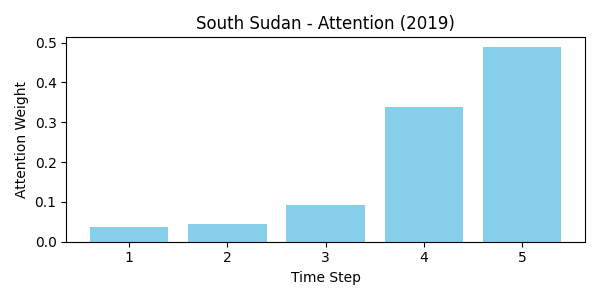

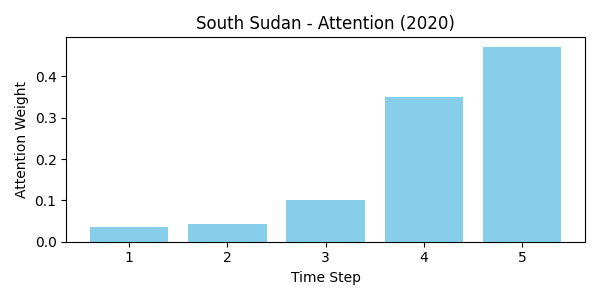

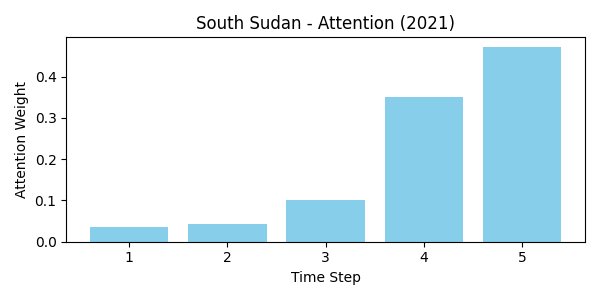

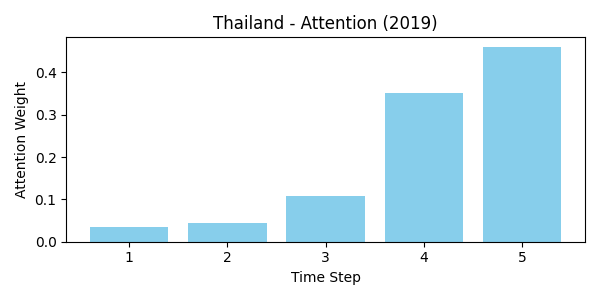


**Figure S2.** ConvLSTM Hidden States, Activation, and Attention Weights for High Incidence and Low incidence Countries (2019–2021)

**Table S11.** ConvLSTM Hidden States, Activation, and Attention Weights for Selected Countries (2019–2021)

| **Country** | **Year** | **Attention_t1** | **Attention_t2** | **Attention_t3** | **Attention_t4** | **Attention_t5** | **HiddenMean_t1** | **ActMean_t1** | **ActMax_t1** | **ActMin_t1** | **HiddenMean_t2** | **ActMean_t2** | **ActMax_t2** | **ActMin_t2** | **HiddenMean_t3** | **ActMean_t3** | **ActMax_t3** | **ActMin_t3** | **HiddenMean_t4** | **ActMean_t4** | **ActMax_t4** | **ActMin_t4** | **HiddenMean_t5** | **ActMean_t5** | **ActMax_t5** | **ActMin_t5** |
| --- | --- | --- | --- | --- | --- | --- | --- | --- | --- | --- | --- | --- | --- | --- | --- | --- | --- | --- | --- | --- | --- | --- | --- | --- | --- | --- |
| Antigua and Barbuda | 2019 | 0.0364 | 0.0451 | 0.1033 | 0.3475 | 0.4676 | 0.0048 | 0.0048 | 0.1371 | -0.1221 | 0.0100 | 0.0100 | 0.3499 | -0.3303 | 0.0148 | 0.0148 | 0.7833 | -0.7743 | 0.0018 | 0.0018 | 0.9813 | -0.9797 | -0.0075 | -0.0075 | 0.9976 | -0.9973 |
| Antigua and Barbuda | 2020 | 0.0369 | 0.0450 | 0.1015 | 0.3458 | 0.4708 | 0.0048 | 0.0048 | 0.1464 | -0.1340 | 0.0096 | 0.0096 | 0.3396 | -0.3409 | 0.0152 | 0.0152 | 0.7756 | -0.7764 | 0.0021 | 0.0021 | 0.9804 | -0.9805 | -0.0070 | -0.0070 | 0.9974 | -0.9974 |
| Antigua and Barbuda | 2021 | 0.0368 | 0.0449 | 0.1002 | 0.3450 | 0.4731 | 0.0049 | 0.0049 | 0.1276 | -0.1278 | 0.0106 | 0.0106 | 0.3374 | -0.3501 | 0.0157 | 0.0157 | 0.7704 | -0.7775 | 0.0034 | 0.0034 | 0.9794 | -0.9807 | -0.0074 | -0.0074 | 0.9972 | -0.9975 |
| Brazil | 2019 | 0.0360 | 0.0459 | 0.1109 | 0.3516 | 0.4556 | 0.0045 | 0.0045 | 0.2110 | -0.1479 | 0.0107 | 0.0107 | 0.4631 | -0.3829 | 0.0143 | 0.0143 | 0.8505 | -0.8182 | 0.0008 | 0.0008 | 0.9878 | -0.9841 | -0.0075 | -0.0075 | 0.9984 | -0.9979 |
| Brazil | 2020 | 0.0364 | 0.0458 | 0.1096 | 0.3509 | 0.4573 | 0.0054 | 0.0054 | 0.1984 | -0.1492 | 0.0097 | 0.0097 | 0.4461 | -0.3908 | 0.0157 | 0.0157 | 0.8442 | -0.8162 | 0.0008 | 0.0008 | 0.9867 | -0.9842 | -0.0073 | -0.0073 | 0.9982 | -0.9979 |
| Brazil | 2021 | 0.0363 | 0.0455 | 0.1070 | 0.3496 | 0.4616 | 0.0047 | 0.0047 | 0.1843 | -0.1417 | 0.0113 | 0.0113 | 0.4386 | -0.3670 | 0.0155 | 0.0155 | 0.8372 | -0.8030 | 0.0019 | 0.0019 | 0.9862 | -0.9828 | -0.0074 | -0.0074 | 0.9982 | -0.9977 |
| Cook Islands | 2019 | 0.0355 | 0.0440 | 0.1001 | 0.3469 | 0.4734 | 0.0046 | 0.0046 | 0.1342 | -0.1493 | 0.0100 | 0.0100 | 0.3626 | -0.3839 | 0.0160 | 0.0160 | 0.7788 | -0.8131 | 0.0043 | 0.0043 | 0.9814 | -0.9835 | -0.0073 | -0.0073 | 0.9975 | -0.9978 |
| Cook Islands | 2020 | 0.0361 | 0.0436 | 0.1044 | 0.3503 | 0.4657 | 0.0043 | 0.0043 | 0.1827 | -0.1762 | 0.0083 | 0.0083 | 0.3430 | -0.4313 | 0.0154 | 0.0154 | 0.7963 | -0.8371 | 0.0018 | 0.0018 | 0.9822 | -0.9861 | -0.0077 | -0.0077 | 0.9978 | -0.9982 |
| Cook Islands | 2021 | 0.0353 | 0.0439 | 0.1030 | 0.3491 | 0.4688 | 0.0040 | 0.0040 | 0.1422 | -0.1704 | 0.0091 | 0.0091 | 0.3947 | -0.4320 | 0.0156 | 0.0156 | 0.7943 | -0.8411 | 0.0029 | 0.0029 | 0.9815 | -0.9864 | -0.0074 | -0.0074 | 0.9977 | -0.9982 |
| India | 2019 | 0.0336 | 0.0525 | 0.1640 | 0.3519 | 0.3981 | 0.0058 | 0.0058 | 0.2849 | -0.2894 | 0.0152 | 0.0152 | 0.7177 | -0.6719 | 0.0140 | 0.0140 | 0.9601 | -0.9567 | -0.0032 | -0.0032 | 0.9957 | -0.9958 | -0.0078 | -0.0078 | 0.9994 | -0.9994 |
| India | 2020 | 0.0338 | 0.0532 | 0.1674 | 0.3509 | 0.3946 | 0.0067 | 0.0067 | 0.3683 | -0.3063 | 0.0138 | 0.0138 | 0.7300 | -0.6866 | 0.0135 | 0.0135 | 0.9628 | -0.9600 | -0.0038 | -0.0038 | 0.9962 | -0.9961 | -0.0078 | -0.0078 | 0.9995 | -0.9995 |
| India | 2021 | 0.0338 | 0.0524 | 0.1634 | 0.3520 | 0.3984 | 0.0060 | 0.0060 | 0.2993 | -0.2775 | 0.0147 | 0.0147 | 0.7027 | -0.6800 | 0.0133 | 0.0133 | 0.9583 | -0.9573 | -0.0034 | -0.0034 | 0.9959 | -0.9958 | -0.0078 | -0.0078 | 0.9994 | -0.9994 |
| Indonesia | 2019 | 0.0355 | 0.0461 | 0.1139 | 0.3527 | 0.4518 | 0.0045 | 0.0045 | 0.1240 | -0.1284 | 0.0105 | 0.0105 | 0.4132 | -0.4350 | 0.0150 | 0.0150 | 0.8424 | -0.8361 | 0.0007 | 0.0007 | 0.9857 | -0.9859 | -0.0074 | -0.0074 | 0.9981 | -0.9982 |
| Indonesia | 2020 | 0.0360 | 0.0465 | 0.1191 | 0.3539 | 0.4445 | 0.0045 | 0.0045 | 0.1872 | -0.1932 | 0.0088 | 0.0088 | 0.4511 | -0.4674 | 0.0146 | 0.0146 | 0.8613 | -0.8614 | -0.0004 | -0.0004 | 0.9883 | -0.9884 | -0.0072 | -0.0072 | 0.9985 | -0.9985 |
| Indonesia | 2021 | 0.0356 | 0.0461 | 0.1153 | 0.3531 | 0.4498 | 0.0044 | 0.0044 | 0.1640 | -0.1673 | 0.0104 | 0.0104 | 0.4299 | -0.4294 | 0.0149 | 0.0149 | 0.8450 | -0.8418 | 0.0008 | 0.0008 | 0.9870 | -0.9867 | -0.0073 | -0.0073 | 0.9983 | -0.9982 |
| Mexico | 2019 | 0.0370 | 0.0455 | 0.1006 | 0.3438 | 0.4731 | 0.0050 | 0.0050 | 0.1319 | -0.1304 | 0.0110 | 0.0110 | 0.3513 | -0.3250 | 0.0153 | 0.0153 | 0.7807 | -0.7600 | 0.0028 | 0.0028 | 0.9802 | -0.9790 | -0.0072 | -0.0072 | 0.9974 | -0.9973 |
| Mexico | 2020 | 0.0373 | 0.0455 | 0.1019 | 0.3447 | 0.4706 | 0.0056 | 0.0056 | 0.1402 | -0.1288 | 0.0099 | 0.0099 | 0.3653 | -0.3415 | 0.0158 | 0.0158 | 0.7903 | -0.7692 | 0.0020 | 0.0020 | 0.9812 | -0.9802 | -0.0069 | -0.0069 | 0.9975 | -0.9974 |
| Mexico | 2021 | 0.0370 | 0.0456 | 0.1016 | 0.3447 | 0.4711 | 0.0049 | 0.0049 | 0.1412 | -0.1317 | 0.0110 | 0.0110 | 0.3629 | -0.3341 | 0.0149 | 0.0149 | 0.7925 | -0.7663 | 0.0029 | 0.0029 | 0.9811 | -0.9797 | -0.0072 | -0.0072 | 0.9975 | -0.9974 |
| Niue | 2019 | 0.0369 | 0.0435 | 0.0873 | 0.3321 | 0.5001 | 0.0060 | 0.0060 | 0.1547 | -0.1527 | 0.0120 | 0.0120 | 0.3841 | -0.3929 | 0.0195 | 0.0195 | 0.7233 | -0.7443 | 0.0096 | 0.0096 | 0.9785 | -0.9806 | -0.0064 | -0.0064 | 0.9974 | -0.9978 |
| Niue | 2020 | 0.0373 | 0.0428 | 0.0905 | 0.3365 | 0.4929 | 0.0058 | 0.0058 | 0.1967 | -0.1751 | 0.0109 | 0.0109 | 0.3370 | -0.3831 | 0.0195 | 0.0195 | 0.7799 | -0.7673 | 0.0065 | 0.0065 | 0.9793 | -0.9803 | -0.0067 | -0.0067 | 0.9977 | -0.9978 |
| Niue | 2021 | 0.0362 | 0.0436 | 0.0910 | 0.3379 | 0.4913 | 0.0056 | 0.0056 | 0.1343 | -0.1521 | 0.0114 | 0.0114 | 0.4413 | -0.4117 | 0.0184 | 0.0184 | 0.7493 | -0.7580 | 0.0074 | 0.0074 | 0.9819 | -0.9822 | -0.0069 | -0.0069 | 0.9978 | -0.9979 |
| Saint Kitts and Nevis | 2019 | 0.0382 | 0.0442 | 0.0865 | 0.3258 | 0.5053 | 0.0063 | 0.0063 | 0.1555 | -0.1623 | 0.0123 | 0.0123 | 0.3450 | -0.3782 | 0.0197 | 0.0197 | 0.7126 | -0.7373 | 0.0092 | 0.0092 | 0.9760 | -0.9789 | -0.0062 | -0.0062 | 0.9972 | -0.9977 |
| Saint Kitts and Nevis | 2020 | 0.0381 | 0.0439 | 0.0868 | 0.3263 | 0.5049 | 0.0068 | 0.0068 | 0.1558 | -0.1545 | 0.0124 | 0.0124 | 0.3088 | -0.3679 | 0.0200 | 0.0200 | 0.7327 | -0.7420 | 0.0087 | 0.0087 | 0.9756 | -0.9790 | -0.0062 | -0.0062 | 0.9972 | -0.9977 |
| Saint Kitts and Nevis | 2021 | 0.0377 | 0.0442 | 0.0873 | 0.3275 | 0.5033 | 0.0067 | 0.0067 | 0.1560 | -0.1471 | 0.0122 | 0.0122 | 0.3630 | -0.3741 | 0.0197 | 0.0197 | 0.7227 | -0.7448 | 0.0087 | 0.0087 | 0.9773 | -0.9796 | -0.0062 | -0.0062 | 0.9973 | -0.9977 |
| South Sudan | 2019 | 0.0371 | 0.0435 | 0.0928 | 0.3380 | 0.4886 | 0.0049 | 0.0049 | 0.1473 | -0.1661 | 0.0100 | 0.0100 | 0.3754 | -0.4308 | 0.0171 | 0.0171 | 0.7663 | -0.8473 | 0.0065 | 0.0065 | 0.9788 | -0.9869 | -0.0064 | -0.0064 | 0.9973 | -0.9983 |
| South Sudan | 2020 | 0.0351 | 0.0425 | 0.1014 | 0.3499 | 0.4712 | 0.0054 | 0.0054 | 0.2525 | -0.2077 | 0.0103 | 0.0103 | 0.5036 | -0.4940 | 0.0177 | 0.0177 | 0.7948 | -0.8519 | 0.0042 | 0.0042 | 0.9831 | -0.9890 | -0.0070 | -0.0070 | 0.9978 | -0.9986 |
| South Sudan | 2021 | 0.0350 | 0.0425 | 0.1009 | 0.3502 | 0.4713 | 0.0050 | 0.0050 | 0.2667 | -0.2003 | 0.0110 | 0.0110 | 0.3413 | -0.3871 | 0.0184 | 0.0184 | 0.7692 | -0.8283 | 0.0043 | 0.0043 | 0.9794 | -0.9857 | -0.0072 | -0.0072 | 0.9974 | -0.9981 |
| Thailand | 2019 | 0.0360 | 0.0454 | 0.1077 | 0.3509 | 0.4601 | 0.0045 | 0.0045 | 0.1722 | -0.1276 | 0.0107 | 0.0107 | 0.4316 | -0.3437 | 0.0149 | 0.0149 | 0.8292 | -0.7919 | 0.0015 | 0.0015 | 0.9863 | -0.9818 | -0.0073 | -0.0073 | 0.9982 | -0.9976 |
| Thailand | 2020 | 0.0364 | 0.0454 | 0.1086 | 0.3511 | 0.4584 | 0.0051 | 0.0051 | 0.1915 | -0.1423 | 0.0094 | 0.0094 | 0.4123 | -0.3712 | 0.0159 | 0.0159 | 0.8229 | -0.8038 | 0.0010 | 0.0010 | 0.9858 | -0.9832 | -0.0070 | -0.0070 | 0.9981 | -0.9978 |
| Thailand | 2021 | 0.0362 | 0.0453 | 0.1060 | 0.3496 | 0.4628 | 0.0046 | 0.0046 | 0.1428 | -0.1353 | 0.0109 | 0.0109 | 0.3606 | -0.3555 | 0.0155 | 0.0155 | 0.8211 | -0.7925 | 0.0022 | 0.0022 | 0.9839 | -0.9820 | -0.0073 | -0.0073 | 0.9979 | -0.9976 |

**Table S12.** SHAP Feature contributions (%) to predicte dengue incidence (Both, male female).

| Both | | Male | | Female | |
| --- | --- | --- | --- | --- | --- |
| Feature | Contribution | Feature | Contribution | Feature | Contribution |
| x20 | 44.37 | x20 | 46.01 | x7 | 31.86 |
| x3 | 16.56 | x7 | 19.62 | x20 | 25.27 |
| x19 | 14.74 | x14 | 6.58 | x14 | 7.72 |
| x13 | 5.18 | x19 | 6.22 | x12 | 6.19 |
| x10 | 4.88 | x9 | 4.94 | x11 | 5.23 |
| x17 | 4.76 | x17 | 4.20 | x8 | 4.55 |
| x16 | 4.23 | x5 | 3.75 | x19 | 3.93 |
| x9 | 1.85 | x10 | 2.30 | x9 | 3.56 |
| x12 | 1.74 | x8 | 2.28 | x17 | 3.27 |
| x14 | 0.98 | x12 | 2.21 | x16 | 3.20 |

*x3: Temperature anomaly; x5: Agricultural land (% of land area); x7: Hospital bed density (per 10,000 population); x8: Density of physicians (per 10,000 population); x9: Domestic general government health expenditure (%); x10: Life expectancy at birth (years); x11: Mortality rate under 5 per 1000 live births; x12: UHC Service Coverage Index (SDG 3.8.1); x13: Population, total; x14: GDP growth (annual %); x16: Access to electricity; x17: Average number of years adults aged 25+ spent in formal education; x19: Urban population (% of total population); x20: Annual freshwater withdrawals, total (billion cubic meters).*

**Figure S3.** SHAP Feature Correlation Matrix for Total Dengue Incidence

**Table S13.** SHAP Feature Correlation Matrix for Total Dengue Incidence

|  | **x1** | **x2** | **x3** | **x4** | **x5** | **x6** | **x7** | **x8** | **x9** | **x10** | **x11** | **x12** | **x13** | **x14** | **x15** | **x16** | **x17** | **x18** | **x19** | **x20** |
| --- | --- | --- | --- | --- | --- | --- | --- | --- | --- | --- | --- | --- | --- | --- | --- | --- | --- | --- | --- | --- |
| **x1** | 1.000 | 0.306 | -0.035 | 0.276 | 0.111 | 0.488 | -0.131 | 0.120 | -0.164 | -0.031 | 0.066 | 0.066 | -0.060 | 0.389 | 0.024 | 0.013 | 0.262 | -0.034 | 0.413 | -0.111 |
| **x2** | 0.306 | 1.000 | 0.047 | 0.031 | 0.134 | 0.222 | -0.098 | -0.005 | -0.144 | -0.091 | 0.106 | 0.107 | 0.015 | 0.307 | 0.129 | 0.021 | 0.176 | -0.029 | 0.208 | -0.070 |
| **x3** | -0.035 | 0.047 | 1.000 | -0.304 | -0.051 | 0.060 | -0.048 | -0.094 | -0.114 | 0.050 | -0.028 | -0.122 | -0.118 | -0.149 | -0.054 | -0.115 | -0.175 | 0.014 | -0.042 | -0.042 |
| **x4** | 0.276 | 0.031 | -0.304 | 1.000 | 0.109 | 0.146 | 0.037 | 0.233 | 0.160 | 0.059 | 0.328 | 0.037 | 0.068 | 0.254 | 0.068 | -0.061 | 0.292 | 0.032 | 0.112 | 0.048 |
| **x5** | 0.111 | 0.134 | -0.051 | 0.109 | 1.000 | 0.051 | 0.042 | 0.062 | 0.016 | 0.074 | 0.154 | -0.058 | -0.177 | 0.112 | 0.111 | -0.018 | 0.110 | 0.203 | 0.280 | -0.045 |
| **x6** | 0.488 | 0.222 | 0.060 | 0.146 | 0.051 | 1.000 | -0.025 | 0.018 | -0.116 | -0.121 | 0.021 | -0.208 | -0.216 | 0.199 | 0.030 | 0.056 | 0.148 | -0.117 | 0.209 | -0.012 |
| **x7** | -0.131 | -0.098 | -0.048 | 0.037 | 0.042 | -0.025 | 1.000 | -0.064 | 0.027 | -0.030 | 0.301 | 0.025 | 0.075 | 0.066 | -0.032 | -0.020 | -0.149 | -0.039 | -0.047 | 0.938 |
| **x8** | 0.120 | -0.005 | -0.094 | 0.233 | 0.062 | 0.018 | -0.064 | 1.000 | 0.068 | -0.013 | 0.184 | 0.155 | 0.054 | 0.153 | 0.065 | 0.005 | 0.030 | 0.036 | -0.020 | -0.047 |
| **x9** | -0.164 | -0.144 | -0.114 | 0.160 | 0.016 | -0.116 | 0.027 | 0.068 | 1.000 | 0.145 | 0.093 | 0.136 | 0.277 | 0.067 | 0.132 | 0.008 | 0.149 | 0.020 | 0.031 | -0.009 |
| **x10** | -0.031 | -0.091 | 0.050 | 0.059 | 0.074 | -0.121 | -0.030 | -0.013 | 0.145 | 1.000 | 0.062 | 0.184 | 0.114 | 0.062 | 0.062 | -0.073 | 0.165 | 0.032 | 0.013 | -0.029 |
| **x11** | 0.066 | 0.106 | -0.028 | 0.328 | 0.154 | 0.021 | 0.301 | 0.184 | 0.093 | 0.062 | 1.000 | -0.064 | 0.039 | 0.242 | -0.038 | -0.143 | 0.182 | 0.013 | 0.168 | 0.259 |
| **x12** | 0.066 | 0.107 | -0.122 | 0.037 | -0.058 | -0.208 | 0.025 | 0.155 | 0.136 | 0.184 | -0.064 | 1.000 | 0.383 | 0.432 | 0.320 | 0.204 | 0.454 | 0.197 | 0.297 | 0.035 |
| **x13** | -0.060 | 0.015 | -0.118 | 0.068 | -0.177 | -0.216 | 0.075 | 0.054 | 0.277 | 0.114 | 0.039 | 0.383 | 1.000 | 0.148 | 0.111 | 0.028 | 0.253 | 0.043 | 0.024 | 0.123 |
| **x14** | 0.389 | 0.307 | -0.149 | 0.254 | 0.112 | 0.199 | 0.066 | 0.153 | 0.067 | 0.062 | 0.242 | 0.432 | 0.148 | 1.000 | 0.220 | 0.124 | 0.442 | 0.104 | 0.567 | 0.044 |
| **x15** | 0.024 | 0.129 | -0.054 | 0.068 | 0.111 | 0.030 | -0.032 | 0.065 | 0.132 | 0.062 | -0.038 | 0.320 | 0.111 | 0.220 | 1.000 | 0.378 | 0.297 | 0.174 | 0.237 | -0.053 |
| **x16** | 0.013 | 0.021 | -0.115 | -0.061 | -0.018 | 0.056 | -0.020 | 0.005 | 0.008 | -0.073 | -0.143 | 0.204 | 0.028 | 0.124 | 0.378 | 1.000 | 0.015 | 0.088 | 0.115 | 0.003 |
| **x17** | 0.262 | 0.176 | -0.175 | 0.292 | 0.110 | 0.148 | -0.149 | 0.030 | 0.149 | 0.165 | 0.182 | 0.454 | 0.253 | 0.442 | 0.297 | 0.015 | 1.000 | 0.100 | 0.474 | -0.163 |
| **x18** | -0.034 | -0.029 | 0.014 | 0.032 | 0.203 | -0.117 | -0.039 | 0.036 | 0.020 | 0.032 | 0.013 | 0.197 | 0.043 | 0.104 | 0.174 | 0.088 | 0.100 | 1.000 | 0.077 | -0.090 |
| **x19** | 0.413 | 0.208 | -0.042 | 0.112 | 0.280 | 0.209 | -0.047 | -0.020 | 0.031 | 0.013 | 0.168 | 0.297 | 0.024 | 0.567 | 0.237 | 0.115 | 0.474 | 0.077 | 1.000 | -0.112 |
| **x20** | -0.111 | -0.070 | -0.042 | 0.048 | -0.045 | -0.012 | 0.938 | -0.047 | -0.009 | -0.029 | 0.259 | 0.035 | 0.123 | 0.044 | -0.053 | 0.003 | -0.163 | -0.090 | -0.112 | 1.000 |

**Table S14.** SHAP Feature Correlation Matrix for male Dengue Incidence

|  | **x1** | **x2** | **x3** | **x4** | **x5** | **x6** | **x7** | **x8** | **x9** | **x10** | **x11** | **x12** | **x13** | **x14** | **x15** | **x16** | **x17** | **x18** | **x19** | **x20** |
| --- | --- | --- | --- | --- | --- | --- | --- | --- | --- | --- | --- | --- | --- | --- | --- | --- | --- | --- | --- | --- |
| **x1** | 1.000 | 0.257 | 0.011 | 0.336 | 0.069 | 0.333 | -0.173 | 0.084 | -0.140 | -0.066 | 0.124 | 0.102 | -0.001 | 0.104 | 0.046 | 0.023 | 0.157 | -0.048 | 0.117 | -0.155 |
| **x2** | 0.257 | 1.000 | -0.022 | 0.009 | 0.004 | 0.001 | -0.070 | -0.029 | -0.179 | -0.168 | 0.264 | 0.071 | 0.113 | 0.164 | 0.114 | 0.043 | 0.013 | 0.025 | 0.071 | -0.039 |
| **x3** | 0.011 | -0.022 | 1.000 | -0.116 | -0.017 | 0.153 | -0.095 | 0.013 | -0.115 | 0.009 | -0.015 | -0.056 | -0.128 | 0.017 | -0.045 | -0.093 | -0.061 | 0.050 | 0.134 | -0.077 |
| **x4** | 0.336 | 0.009 | -0.116 | 1.000 | 0.124 | 0.123 | 0.038 | 0.162 | 0.178 | 0.049 | 0.244 | 0.064 | 0.060 | 0.134 | 0.052 | -0.079 | 0.144 | 0.055 | -0.045 | 0.040 |
| **x5** | 0.069 | 0.004 | -0.017 | 0.124 | 1.000 | 0.046 | 0.067 | -0.001 | -0.039 | 0.155 | 0.091 | -0.100 | -0.247 | -0.179 | 0.024 | -0.030 | -0.033 | 0.177 | 0.029 | -0.017 |
| **x6** | 0.333 | 0.001 | 0.153 | 0.123 | 0.046 | 1.000 | 0.086 | -0.074 | -0.103 | -0.072 | -0.068 | -0.230 | -0.261 | -0.226 | -0.023 | -0.012 | -0.169 | -0.041 | -0.062 | 0.098 |
| **x7** | -0.173 | -0.070 | -0.095 | 0.038 | 0.067 | 0.086 | 1.000 | -0.050 | 0.045 | -0.071 | 0.099 | 0.065 | 0.105 | 0.101 | -0.012 | 0.048 | -0.070 | -0.031 | -0.053 | 0.952 |
| **x8** | 0.084 | -0.029 | 0.013 | 0.162 | -0.001 | -0.074 | -0.050 | 1.000 | 0.070 | -0.058 | 0.098 | 0.100 | 0.081 | 0.161 | 0.048 | -0.029 | 0.085 | 0.041 | -0.087 | -0.040 |
| **x9** | -0.140 | -0.179 | -0.115 | 0.178 | -0.039 | -0.103 | 0.045 | 0.070 | 1.000 | 0.164 | 0.031 | 0.060 | 0.164 | 0.034 | 0.082 | -0.011 | 0.100 | 0.000 | -0.067 | 0.012 |
| **x10** | -0.066 | -0.168 | 0.009 | 0.049 | 0.155 | -0.072 | -0.071 | -0.058 | 0.164 | 1.000 | -0.014 | 0.014 | 0.005 | -0.141 | 0.007 | -0.095 | 0.008 | -0.073 | -0.052 | -0.074 |
| **x11** | 0.124 | 0.264 | -0.015 | 0.244 | 0.091 | -0.068 | 0.099 | 0.098 | 0.031 | -0.014 | 1.000 | -0.096 | 0.061 | 0.075 | -0.048 | -0.029 | 0.030 | 0.069 | -0.077 | 0.100 |
| **x12** | 0.102 | 0.071 | -0.056 | 0.064 | -0.100 | -0.230 | 0.065 | 0.100 | 0.060 | 0.014 | -0.096 | 1.000 | 0.526 | 0.665 | 0.245 | 0.117 | 0.711 | 0.216 | 0.448 | 0.090 |
| **x13** | -0.001 | 0.113 | -0.128 | 0.060 | -0.247 | -0.261 | 0.105 | 0.081 | 0.164 | 0.005 | 0.061 | 0.526 | 1.000 | 0.606 | 0.146 | 0.063 | 0.484 | -0.054 | 0.223 | 0.146 |
| **x14** | 0.104 | 0.164 | 0.017 | 0.134 | -0.179 | -0.226 | 0.101 | 0.161 | 0.034 | -0.141 | 0.075 | 0.665 | 0.606 | 1.000 | 0.224 | 0.168 | 0.593 | 0.185 | 0.421 | 0.151 |
| **x15** | 0.046 | 0.114 | -0.045 | 0.052 | 0.024 | -0.023 | -0.012 | 0.048 | 0.082 | 0.007 | -0.048 | 0.245 | 0.146 | 0.224 | 1.000 | 0.512 | 0.310 | 0.171 | 0.149 | -0.027 |
| **x16** | 0.023 | 0.043 | -0.093 | -0.079 | -0.030 | -0.012 | 0.048 | -0.029 | -0.011 | -0.095 | -0.029 | 0.117 | 0.063 | 0.168 | 0.512 | 1.000 | 0.105 | 0.063 | 0.062 | 0.077 |
| **x17** | 0.157 | 0.013 | -0.061 | 0.144 | -0.033 | -0.169 | -0.070 | 0.085 | 0.100 | 0.008 | 0.030 | 0.711 | 0.484 | 0.593 | 0.310 | 0.105 | 1.000 | 0.180 | 0.455 | -0.068 |
| **x18** | -0.048 | 0.025 | 0.050 | 0.055 | 0.177 | -0.041 | -0.031 | 0.041 | 0.000 | -0.073 | 0.069 | 0.216 | -0.054 | 0.185 | 0.171 | 0.063 | 0.180 | 1.000 | 0.167 | -0.093 |
| **x19** | 0.117 | 0.071 | 0.134 | -0.045 | 0.029 | -0.062 | -0.053 | -0.087 | -0.067 | -0.052 | -0.077 | 0.448 | 0.223 | 0.421 | 0.149 | 0.062 | 0.455 | 0.167 | 1.000 | -0.061 |
| **x20** | -0.155 | -0.039 | -0.077 | 0.040 | -0.017 | 0.098 | 0.952 | -0.040 | 0.012 | -0.074 | 0.100 | 0.090 | 0.146 | 0.151 | -0.027 | 0.077 | -0.068 | -0.093 | -0.061 | 1.000 |

**Table S15.** SHAP Feature Correlation Matrix for femaleDengue Incidence

|  | **x1** | **x2** | **x3** | **x4** | **x5** | **x6** | **x7** | **x8** | **x9** | **x10** | **x11** | **x12** | **x13** | **x14** | **x15** | **x16** | **x17** | **x18** | **x19** | **x20** |
| --- | --- | --- | --- | --- | --- | --- | --- | --- | --- | --- | --- | --- | --- | --- | --- | --- | --- | --- | --- | --- |
| **x1** | 1.000 | 0.248 | -0.231 | 0.346 | 0.092 | 0.351 | -0.160 | 0.120 | -0.163 | 0.008 | 0.043 | 0.084 | -0.042 | 0.139 | 0.069 | 0.018 | 0.175 | -0.043 | 0.262 | -0.091 |
| **x2** | 0.248 | 1.000 | 0.094 | -0.031 | 0.157 | 0.040 | -0.082 | -0.028 | -0.136 | -0.030 | 0.136 | 0.035 | 0.027 | 0.077 | 0.124 | -0.009 | 0.034 | -0.055 | 0.188 | -0.055 |
| **x3** | -0.231 | 0.094 | 1.000 | -0.138 | 0.030 | 0.110 | -0.026 | -0.076 | 0.027 | -0.011 | -0.135 | -0.381 | -0.218 | -0.307 | -0.126 | -0.178 | -0.375 | -0.098 | -0.194 | -0.098 |
| **x4** | 0.346 | -0.031 | -0.138 | 1.000 | 0.118 | 0.204 | -0.044 | 0.120 | -0.057 | 0.065 | 0.143 | -0.117 | -0.187 | -0.006 | 0.099 | -0.064 | 0.021 | -0.025 | 0.011 | -0.006 |
| **x5** | 0.092 | 0.157 | 0.030 | 0.118 | 1.000 | 0.067 | 0.035 | -0.002 | -0.042 | 0.051 | 0.112 | -0.128 | -0.278 | -0.127 | 0.044 | -0.050 | -0.014 | 0.195 | 0.188 | -0.163 |
| **x6** | 0.351 | 0.040 | 0.110 | 0.204 | 0.067 | 1.000 | 0.029 | -0.093 | -0.081 | -0.066 | -0.076 | -0.284 | -0.213 | -0.183 | 0.006 | 0.019 | -0.174 | -0.024 | -0.003 | -0.029 |
| **x7** | -0.160 | -0.082 | -0.026 | -0.044 | 0.035 | 0.029 | 1.000 | -0.123 | 0.065 | -0.121 | 0.230 | 0.119 | 0.172 | 0.251 | -0.046 | 0.117 | -0.022 | -0.004 | -0.074 | 0.661 |
| **x8** | 0.120 | -0.028 | -0.076 | 0.120 | -0.002 | -0.093 | -0.123 | 1.000 | -0.055 | -0.042 | 0.013 | 0.232 | 0.097 | 0.217 | 0.108 | 0.005 | 0.159 | 0.029 | 0.012 | -0.096 |
| **x9** | -0.163 | -0.136 | 0.027 | -0.057 | -0.042 | -0.081 | 0.065 | -0.055 | 1.000 | 0.107 | 0.013 | 0.026 | 0.209 | 0.065 | 0.095 | 0.009 | 0.094 | -0.009 | -0.009 | 0.027 |
| **x10** | 0.008 | -0.030 | -0.011 | 0.065 | 0.051 | -0.066 | -0.121 | -0.042 | 0.107 | 1.000 | 0.025 | -0.146 | -0.055 | -0.110 | -0.154 | -0.186 | -0.045 | -0.083 | -0.061 | -0.192 |
| **x11** | 0.043 | 0.136 | -0.135 | 0.143 | 0.112 | -0.076 | 0.230 | 0.013 | 0.013 | 0.025 | 1.000 | -0.078 | 0.070 | 0.169 | 0.002 | 0.075 | 0.050 | 0.063 | 0.003 | 0.260 |
| **x12** | 0.084 | 0.035 | -0.381 | -0.117 | -0.128 | -0.284 | 0.119 | 0.232 | 0.026 | -0.146 | -0.078 | 1.000 | 0.518 | 0.742 | 0.251 | 0.225 | 0.694 | 0.168 | 0.388 | 0.178 |
| **x13** | -0.042 | 0.027 | -0.218 | -0.187 | -0.278 | -0.213 | 0.172 | 0.097 | 0.209 | -0.055 | 0.070 | 0.518 | 1.000 | 0.536 | 0.062 | 0.052 | 0.414 | -0.056 | 0.116 | 0.253 |
| **x14** | 0.139 | 0.077 | -0.307 | -0.006 | -0.127 | -0.183 | 0.251 | 0.217 | 0.065 | -0.110 | 0.169 | 0.742 | 0.536 | 1.000 | 0.105 | 0.138 | 0.593 | 0.133 | 0.382 | 0.325 |
| **x15** | 0.069 | 0.124 | -0.126 | 0.099 | 0.044 | 0.006 | -0.046 | 0.108 | 0.095 | -0.154 | 0.002 | 0.251 | 0.062 | 0.105 | 1.000 | 0.542 | 0.223 | 0.149 | 0.181 | -0.049 |
| **x16** | 0.018 | -0.009 | -0.178 | -0.064 | -0.050 | 0.019 | 0.117 | 0.005 | 0.009 | -0.186 | 0.075 | 0.225 | 0.052 | 0.138 | 0.542 | 1.000 | 0.054 | 0.060 | 0.062 | 0.232 |
| **x17** | 0.175 | 0.034 | -0.375 | 0.021 | -0.014 | -0.174 | -0.022 | 0.159 | 0.094 | -0.045 | 0.050 | 0.694 | 0.414 | 0.593 | 0.223 | 0.054 | 1.000 | 0.192 | 0.513 | 0.016 |
| **x18** | -0.043 | -0.055 | -0.098 | -0.025 | 0.195 | -0.024 | -0.004 | 0.029 | -0.009 | -0.083 | 0.063 | 0.168 | -0.056 | 0.133 | 0.149 | 0.060 | 0.192 | 1.000 | 0.187 | -0.099 |
| **x19** | 0.262 | 0.188 | -0.194 | 0.011 | 0.188 | -0.003 | -0.074 | 0.012 | -0.009 | -0.061 | 0.003 | 0.388 | 0.116 | 0.382 | 0.181 | 0.062 | 0.513 | 0.187 | 1.000 | -0.106 |
| **x20** | -0.091 | -0.055 | -0.098 | -0.006 | -0.163 | -0.029 | 0.661 | -0.096 | 0.027 | -0.192 | 0.260 | 0.178 | 0.253 | 0.325 | -0.049 | 0.232 | 0.016 | -0.099 | -0.106 | 1.000 |

**Table S16.** Country wise SHAP feature contributions (%) to predict dengue incidence (both sex total).

| **Country** | **x20** | **x3** | **x19** | **x13** | **x10** | **x17** | **x16** | **x9** | **x12** | **x14** |
| --- | --- | --- | --- | --- | --- | --- | --- | --- | --- | --- |
| Afghanistan | 0.00 | 0.00 | 1.56 | 56.26 | 0.00 | 17.85 | 24.34 | 0.00 | 0.00 | 0.00 |
| Angola | 0.00 | 37.14 | 0.31 | 1.22 | 49.62 | 5.14 | 6.57 | 0.00 | 0.00 | 0.00 |
| Antigua and Barbuda | 0.00 | 32.13 | 0.06 | 0.05 | 0.00 | 0.09 | 0.00 | 67.66 | 0.00 | 0.00 |
| Argentina | 0.00 | 0.00 | 0.00 | 8.15 | 0.00 | 1.98 | 87.73 | 0.00 | 2.14 | 0.00 |
| Australia | 0.00 | 91.76 | 0.00 | 0.00 | 6.02 | 2.22 | 0.00 | 0.00 | 0.00 | 0.00 |
| Bahamas | 0.00 | 5.92 | 0.00 | 6.20 | 74.27 | 2.88 | 10.73 | 0.00 | 0.00 | 0.00 |
| Bangladesh | 0.00 | 28.21 | 0.00 | 0.58 | 2.66 | 2.67 | 52.11 | 0.00 | 13.79 | 0.00 |
| Barbados | 0.00 | 0.10 | 0.00 | 0.00 | 80.71 | 2.00 | 10.51 | 0.00 | 6.69 | 0.00 |
| Belize | 0.00 | 0.00 | 0.00 | 2.27 | 92.61 | 0.00 | 4.97 | 0.00 | 0.15 | 0.00 |
| Benin | 0.00 | 0.00 | 2.03 | 0.00 | 0.00 | 0.00 | 52.62 | 0.00 | 45.35 | 0.00 |
| Bhutan | 0.00 | 81.59 | 11.80 | 1.50 | 0.00 | 0.00 | 2.15 | 0.00 | 2.96 | 0.00 |
| Bolivia (Plurinational State of) | 0.00 | 0.17 | 0.00 | 0.45 | 33.74 | 0.00 | 29.39 | 36.24 | 0.00 | 0.00 |
| Brazil | 0.00 | 40.00 | 0.00 | 0.00 | 30.41 | 2.90 | 21.64 | 4.80 | 0.00 | 0.24 |
| Brunei Darussalam | 0.00 | 0.91 | 0.00 | 8.56 | 0.00 | 0.00 | 0.00 | 90.10 | 0.43 | 0.00 |
| Burkina Faso | 0.00 | 0.00 | 4.66 | 44.13 | 5.75 | 13.84 | 0.00 | 17.35 | 14.26 | 0.00 |
| Burundi | 0.00 | 0.00 | 0.17 | 68.01 | 0.00 | 0.66 | 24.45 | 0.00 | 5.19 | 1.53 |
| Cabo Verde | 0.00 | 0.27 | 0.04 | 1.17 | 11.06 | 0.00 | 0.00 | 80.88 | 0.00 | 6.57 |
| Cambodia | 0.00 | 0.00 | 0.00 | 11.15 | 73.14 | 0.00 | 0.00 | 0.00 | 0.00 | 15.71 |
| Cameroon | 0.00 | 0.00 | 14.20 | 5.96 | 0.00 | 13.14 | 49.37 | 0.00 | 17.33 | 0.00 |
| Central African Republic | 0.00 | 0.00 | 0.16 | 11.01 | 0.00 | 0.00 | 68.87 | 0.00 | 16.01 | 3.96 |
| Chad | 0.00 | 79.92 | 0.72 | 5.92 | 0.47 | 8.38 | 0.00 | 0.00 | 4.60 | 0.00 |
| China | 8.41 | 0.00 | 0.53 | 10.78 | 67.95 | 12.33 | 0.00 | 0.00 | 0.00 | 0.00 |
| Colombia | 0.00 | 0.00 | 0.00 | 10.07 | 47.33 | 42.60 | 0.00 | 0.00 | 0.00 | 0.00 |
| Comoros | 0.00 | 12.33 | 0.00 | 0.85 | 70.34 | 3.85 | 0.00 | 0.00 | 12.63 | 0.00 |
| Congo | 0.00 | 65.91 | 0.00 | 3.95 | 0.00 | 0.00 | 0.00 | 0.00 | 30.14 | 0.00 |
| Cook Islands | 0.00 | 43.92 | 0.00 | 0.37 | 31.89 | 0.00 | 23.82 | 0.00 | 0.00 | 0.00 |
| Costa Rica | 0.00 | 64.67 | 0.00 | 1.91 | 14.79 | 2.25 | 7.59 | 8.79 | 0.00 | 0.00 |
| Cuba | 0.00 | 91.55 | 0.49 | 4.59 | 0.00 | 2.41 | 0.00 | 0.00 | 0.00 | 0.95 |
| Democratic Republic of the Congo | 0.00 | 0.05 | 0.00 | 6.93 | 25.94 | 0.00 | 0.00 | 66.76 | 0.31 | 0.00 |
| Djibouti | 0.00 | 74.55 | 1.03 | 14.95 | 0.00 | 9.47 | 0.00 | 0.00 | 0.00 | 0.00 |
| Dominica | 0.00 | 0.00 | 0.53 | 0.14 | 84.74 | 0.00 | 0.00 | 0.84 | 13.69 | 0.06 |
| Dominican Republic | 0.00 | 0.00 | 4.34 | 0.00 | 43.12 | 44.53 | 0.00 | 0.00 | 4.68 | 3.32 |
| Ecuador | 0.00 | 35.10 | 0.00 | 12.61 | 0.00 | 5.72 | 46.58 | 0.00 | 0.00 | 0.00 |
| Egypt | 0.00 | 0.00 | 0.00 | 9.73 | 45.06 | 5.72 | 39.13 | 0.00 | 0.35 | 0.00 |
| El Salvador | 0.00 | 0.00 | 0.79 | 32.88 | 0.00 | 0.00 | 32.93 | 0.00 | 0.00 | 33.40 |
| Equatorial Guinea | 0.00 | 0.00 | 0.00 | 88.18 | 0.00 | 7.25 | 0.00 | 0.00 | 4.57 | 0.00 |
| Eritrea | 0.00 | 30.86 | 0.00 | 10.67 | 0.03 | 0.00 | 0.00 | 53.32 | 0.00 | 5.12 |
| Ethiopia | 0.00 | 0.00 | 1.10 | 0.00 | 19.61 | 2.09 | 75.52 | 0.00 | 1.67 | 0.00 |
| Fiji | 0.00 | 0.00 | 0.00 | 65.72 | 26.21 | 8.07 | 0.00 | 0.00 | 0.00 | 0.00 |
| Gabon | 0.00 | 0.00 | 0.00 | 0.00 | 95.90 | 0.00 | 0.00 | 0.00 | 0.00 | 4.10 |
| Gambia | 0.00 | 0.00 | 0.00 | 5.60 | 0.00 | 0.00 | 79.63 | 0.00 | 14.77 | 0.00 |
| Ghana | 0.00 | 0.00 | 3.68 | 4.64 | 0.00 | 50.20 | 2.59 | 0.00 | 38.88 | 0.00 |
| Grenada | 0.00 | 0.00 | 0.00 | 8.68 | 0.00 | 0.00 | 0.00 | 81.42 | 5.22 | 4.68 |
| Guatemala | 0.00 | 0.00 | 0.00 | 0.00 | 87.53 | 0.57 | 11.33 | 0.00 | 0.57 | 0.00 |
| Guinea | 0.00 | 0.00 | 0.05 | 0.00 | 94.80 | 3.63 | 0.00 | 0.00 | 1.52 | 0.00 |
| Guinea-Bissau | 0.00 | 80.59 | 5.61 | 13.55 | 0.00 | 0.00 | 0.24 | 0.00 | 0.00 | 0.00 |
| Guyana | 0.00 | 0.00 | 0.07 | 27.86 | 0.00 | 3.45 | 0.00 | 68.02 | 0.59 | 0.00 |
| Haiti | 0.00 | 70.43 | 21.53 | 8.04 | 0.00 | 0.00 | 0.00 | 0.00 | 0.00 | 0.00 |
| Honduras | 0.00 | 59.68 | 3.37 | 1.82 | 0.00 | 0.00 | 34.76 | 0.37 | 0.00 | 0.00 |
| India | 0.00 | 7.26 | 0.00 | 0.00 | 77.66 | 1.10 | 13.97 | 0.00 | 0.01 | 0.00 |
| Indonesia | 0.00 | 0.00 | 0.15 | 3.46 | 59.47 | 36.10 | 0.00 | 0.00 | 0.82 | 0.00 |
| Jamaica | 0.00 | 20.03 | 0.00 | 6.42 | 0.00 | 0.00 | 53.76 | 7.59 | 5.52 | 6.68 |
| Jordan | 0.00 | 0.00 | 0.00 | 0.00 | 87.95 | 0.88 | 11.17 | 0.00 | 0.00 | 0.00 |
| Kenya | 0.00 | 27.05 | 0.46 | 0.00 | 39.99 | 0.00 | 26.97 | 2.15 | 3.38 | 0.00 |
| Kuwait | 0.00 | 0.00 | 0.00 | 61.75 | 0.00 | 28.82 | 0.00 | 0.00 | 9.42 | 0.00 |
| Lao People's Democratic Republic | 0.00 | 0.00 | 0.00 | 84.07 | 0.00 | 0.00 | 15.27 | 0.00 | 0.67 | 0.00 |
| Lebanon | 0.00 | 0.00 | 0.00 | 45.05 | 47.45 | 0.00 | 7.50 | 0.00 | 0.00 | 0.00 |
| Liberia | 0.00 | 22.45 | 0.00 | 2.35 | 0.00 | 0.00 | 59.59 | 0.00 | 5.15 | 10.45 |
| Madagascar | 0.00 | 32.37 | 0.00 | 0.00 | 0.30 | 5.20 | 55.78 | 0.00 | 6.26 | 0.10 |
| Malawi | 0.00 | 0.00 | 0.00 | 0.68 | 79.28 | 0.00 | 0.00 | 0.00 | 0.00 | 20.05 |
| Malaysia | 0.00 | 44.99 | 0.00 | 3.60 | 13.98 | 7.01 | 26.01 | 4.43 | 0.00 | 0.00 |
| Maldives | 0.00 | 0.00 | 0.00 | 2.45 | 29.77 | 15.02 | 52.77 | 0.00 | 0.00 | 0.00 |
| Mali | 0.00 | 29.54 | 2.45 | 0.00 | 60.51 | 0.30 | 0.00 | 0.00 | 7.20 | 0.00 |
| Mauritania | 0.00 | 92.33 | 6.72 | 0.00 | 0.86 | 0.00 | 0.10 | 0.00 | 0.00 | 0.00 |
| Mauritius | 0.00 | 69.42 | 0.00 | 0.39 | 28.72 | 1.14 | 0.00 | 0.00 | 0.18 | 0.13 |
| Mexico | 0.00 | 42.86 | 0.00 | 0.00 | 26.13 | 6.68 | 0.00 | 15.64 | 8.69 | 0.00 |
| Micronesia (Federated States of) | 0.00 | 0.00 | 0.00 | 38.90 | 0.00 | 0.00 | 26.76 | 30.12 | 4.22 | 0.00 |
| Mozambique | 0.00 | 0.00 | 0.00 | 0.00 | 6.30 | 0.00 | 0.00 | 53.42 | 0.00 | 40.28 |
| Myanmar | 0.00 | 88.51 | 0.27 | 0.00 | 0.00 | 0.00 | 0.00 | 0.00 | 0.00 | 11.22 |
| Nepal | 0.00 | 0.00 | 0.00 | 100.00 | 0.00 | 0.00 | 0.00 | 0.00 | 0.00 | 0.00 |
| Nicaragua | 0.03 | 0.00 | 0.00 | 0.07 | 96.26 | 3.63 | 0.00 | 0.00 | 0.00 | 0.00 |
| Niger | 0.23 | 44.46 | 0.00 | 0.00 | 45.53 | 0.00 | 0.00 | 6.40 | 3.38 | 0.00 |
| Nigeria | 0.00 | 0.00 | 7.65 | 0.00 | 0.00 | 92.35 | 0.00 | 0.00 | 0.00 | 0.00 |
| Niue | 0.00 | 0.00 | 0.00 | 0.54 | 0.00 | 0.00 | 99.46 | 0.00 | 0.00 | 0.00 |
| Oman | 0.00 | 0.00 | 0.00 | 49.91 | 7.47 | 0.00 | 41.65 | 0.00 | 0.97 | 0.00 |
| Pakistan | 10.92 | 0.00 | 0.00 | 4.90 | 0.00 | 3.27 | 51.88 | 23.46 | 3.92 | 1.65 |
| Panama | 0.00 | 41.12 | 0.00 | 10.67 | 12.56 | 23.14 | 0.00 | 0.00 | 12.50 | 0.00 |
| Papua New Guinea | 0.00 | 0.00 | 0.23 | 6.89 | 0.18 | 1.01 | 87.17 | 4.52 | 0.00 | 0.00 |
| Paraguay | 0.00 | 0.00 | 0.00 | 0.00 | 98.54 | 1.46 | 0.00 | 0.00 | 0.00 | 0.00 |
| Peru | 11.37 | 0.00 | 0.00 | 23.90 | 47.22 | 14.80 | 0.00 | 2.72 | 0.00 | 0.00 |
| Philippines | 0.38 | 0.00 | 0.00 | 0.75 | 7.61 | 32.32 | 57.94 | 1.01 | 0.00 | 0.00 |
| Rwanda | 0.00 | 0.00 | 0.00 | 0.00 | 98.06 | 0.37 | 0.00 | 0.00 | 1.57 | 0.00 |
| Saint Kitts and Nevis | 0.00 | 0.00 | 0.00 | 0.79 | 92.15 | 0.00 | 1.58 | 0.00 | 5.48 | 0.00 |
| Saint Lucia | 0.00 | 0.00 | 0.00 | 0.00 | 82.86 | 0.18 | 15.38 | 0.00 | 1.57 | 0.00 |
| Saint Vincent and the Grenadines | 0.00 | 0.00 | 0.00 | 0.00 | 100.00 | 0.00 | 0.00 | 0.00 | 0.00 | 0.00 |
| Sao Tome and Principe | 0.00 | 1.47 | 0.78 | 2.49 | 31.81 | 0.00 | 18.64 | 38.66 | 0.00 | 6.15 |
| Saudi Arabia | 0.00 | 18.31 | 0.02 | 31.47 | 0.69 | 0.00 | 49.51 | 0.00 | 0.00 | 0.00 |
| Senegal | 0.00 | 0.00 | 1.46 | 7.82 | 0.00 | 0.00 | 35.95 | 52.34 | 2.43 | 0.00 |
| Seychelles | 0.00 | 0.00 | 0.00 | 0.00 | 36.99 | 2.38 | 0.00 | 0.00 | 60.63 | 0.00 |
| Sierra Leone | 0.00 | 0.00 | 13.28 | 18.71 | 0.00 | 8.19 | 0.00 | 0.00 | 59.82 | 0.00 |
| Singapore | 0.00 | 0.00 | 0.00 | 100.00 | 0.00 | 0.00 | 0.00 | 0.00 | 0.00 | 0.00 |
| Somalia | 0.00 | 55.19 | 0.00 | 0.00 | 28.70 | 0.00 | 7.89 | 0.00 | 3.55 | 4.68 |
| South Sudan | 0.00 | 0.00 | 0.31 | 24.94 | 38.28 | 0.00 | 36.47 | 0.00 | 0.00 | 0.00 |
| Sri Lanka | 0.00 | 0.00 | 0.00 | 5.65 | 8.61 | 2.92 | 0.00 | 80.39 | 2.43 | 0.00 |
| Sudan | 0.00 | 33.62 | 0.00 | 16.62 | 33.60 | 7.25 | 8.91 | 0.00 | 0.00 | 0.00 |
| Suriname | 0.00 | 90.62 | 0.00 | 0.00 | 9.38 | 0.00 | 0.00 | 0.00 | 0.00 | 0.00 |
| Syrian Arab Republic | 0.00 | 0.00 | 0.00 | 73.31 | 0.00 | 0.00 | 0.00 | 0.00 | 0.27 | 26.42 |
| Thailand | 0.00 | 0.00 | 0.00 | 0.67 | 36.62 | 5.05 | 52.28 | 5.38 | 0.00 | 0.00 |
| Timor-Leste | 0.00 | 0.00 | 0.00 | 0.00 | 99.21 | 0.00 | 0.00 | 0.00 | 0.79 | 0.00 |
| Togo | 0.00 | 28.56 | 1.06 | 0.00 | 0.44 | 0.00 | 0.35 | 68.63 | 0.00 | 0.95 |
| Trinidad and Tobago | 0.00 | 96.34 | 0.00 | 2.69 | 0.00 | 0.97 | 0.00 | 0.00 | 0.00 | 0.00 |
| Uganda | 0.00 | 95.60 | 0.90 | 0.72 | 0.00 | 1.83 | 0.00 | 0.00 | 0.95 | 0.00 |
| United Republic of Tanzania | 0.00 | 0.00 | 0.00 | 95.68 | 0.00 | 0.00 | 0.00 | 4.32 | 0.00 | 0.00 |
| United States of America | 0.00 | 0.00 | 0.00 | 45.85 | 40.92 | 0.00 | 0.00 | 13.23 | 0.00 | 0.00 |
| Venezuela (Bolivarian Republic of) | 0.00 | 0.00 | 0.00 | 0.00 | 99.41 | 0.00 | 0.00 | 0.00 | 0.59 | 0.00 |
| Viet Nam | 0.00 | 0.00 | 0.00 | 6.51 | 0.00 | 0.00 | 93.49 | 0.00 | 0.00 | 0.00 |
| Yemen | 0.00 | 0.00 | 0.00 | 4.41 | 0.00 | 5.01 | 88.64 | 0.00 | 1.94 | 0.00 |
| Zambia | 0.00 | 77.70 | 0.00 | 0.00 | 0.62 | 16.80 | 0.00 | 0.00 | 4.88 | 0.00 |
| Zimbabwe | 0.62 | 21.15 | 0.00 | 23.67 | 0.00 | 5.54 | 0.00 | 0.00 | 2.47 | 46.55 |

*x20: Annual freshwater withdrawals, total (billion cubic meters); x3: Temperature anomaly; x19: Urban population (% of total population); x13: Population, total; x10: Life expectancy at birth (years); x17: Average number of years adults aged 25+ spent in formal education; x16: Access to electricity; x9: Domestic general government health expenditure (%); x12: UHC Service Coverage Index (SDG 3.8.1); x14: GDP growth (annual %).*

**Table S17.** Country wise SHAP feature contributions (%) to predict dengue incidence (male).

| **Country** | **x20** | **x3** | **x19** | **x13** | **x10** | **x17** | **x16** | **x9** | **x12** | **x14** |
| --- | --- | --- | --- | --- | --- | --- | --- | --- | --- | --- |
| Afghanistan | 0.00 | 17.59 | 0.23 | 0.00 | 0.00 | 0.00 | 74.96 | 0.00 | 7.21 | 0.00 |
| Angola | 0.00 | 0.00 | 0.00 | 0.00 | 82.78 | 0.00 | 14.14 | 0.00 | 3.08 | 0.00 |
| Antigua and Barbuda | 0.00 | 0.00 | 0.00 | 0.00 | 0.00 | 0.00 | 0.00 | 100.00 | 0.00 | 0.00 |
| Argentina | 0.00 | 65.04 | 0.00 | 0.00 | 0.00 | 0.00 | 34.96 | 0.00 | 0.00 | 0.00 |
| Australia | 1.94 | 0.00 | 0.00 | 0.00 | 6.34 | 0.00 | 91.72 | 0.00 | 0.00 | 0.00 |
| Bahamas | 0.00 | 0.00 | 0.00 | 0.00 | 99.20 | 0.80 | 0.00 | 0.00 | 0.00 | 0.00 |
| Bangladesh | 0.00 | 24.98 | 0.59 | 0.00 | 2.63 | 13.54 | 58.26 | 0.00 | 0.00 | 0.00 |
| Barbados | 0.00 | 0.00 | 0.00 | 0.55 | 97.06 | 1.06 | 1.34 | 0.00 | 0.00 | 0.00 |
| Belize | 0.00 | 0.00 | 0.00 | 0.00 | 46.72 | 1.83 | 51.45 | 0.00 | 0.00 | 0.00 |
| Benin | 0.00 | 0.00 | 14.55 | 0.00 | 85.45 | 0.00 | 0.00 | 0.00 | 0.00 | 0.00 |
| Bhutan | 0.00 | 29.46 | 12.79 | 0.00 | 0.00 | 30.31 | 22.64 | 4.81 | 0.00 | 0.00 |
| Bolivia (Plurinational State of) | 0.00 | 0.12 | 0.00 | 0.00 | 16.63 | 0.00 | 68.43 | 14.82 | 0.00 | 0.00 |
| Brazil | 0.00 | 0.00 | 0.00 | 0.00 | 36.39 | 0.00 | 58.24 | 5.03 | 0.00 | 0.35 |
| Brunei Darussalam | 0.00 | 0.40 | 0.00 | 0.00 | 0.00 | 0.00 | 0.00 | 92.95 | 6.65 | 0.00 |
| Burkina Faso | 0.00 | 62.48 | 0.00 | 0.00 | 0.23 | 0.00 | 0.00 | 37.29 | 0.00 | 0.00 |
| Burundi | 0.00 | 78.63 | 2.43 | 0.00 | 0.00 | 0.00 | 0.00 | 0.00 | 0.00 | 18.94 |
| Cabo Verde | 0.00 | 0.14 | 2.82 | 0.00 | 4.01 | 0.00 | 0.00 | 83.99 | 0.59 | 8.45 |
| Cambodia | 0.00 | 0.00 | 0.00 | 0.00 | 0.00 | 4.51 | 0.00 | 85.76 | 6.84 | 2.89 |
| Cameroon | 0.00 | 81.28 | 0.05 | 0.00 | 0.00 | 0.00 | 18.67 | 0.00 | 0.00 | 0.00 |
| Central African Republic | 0.00 | 6.32 | 2.15 | 0.00 | 19.87 | 0.00 | 71.39 | 0.00 | 0.00 | 0.27 |
| Chad | 0.00 | 0.00 | 0.00 | 100.00 | 0.00 | 0.00 | 0.00 | 0.00 | 0.00 | 0.00 |
| China | 0.00 | 77.74 | 0.00 | 0.00 | 4.73 | 0.00 | 17.53 | 0.00 | 0.00 | 0.00 |
| Colombia | 1.48 | 0.21 | 0.00 | 0.00 | 6.34 | 0.00 | 91.97 | 0.00 | 0.00 | 0.00 |
| Comoros | 0.00 | 0.00 | 0.00 | 0.10 | 22.59 | 0.00 | 76.84 | 0.00 | 0.00 | 0.46 |
| Congo | 0.00 | 0.00 | 0.00 | 0.00 | 62.56 | 0.91 | 36.52 | 0.00 | 0.00 | 0.00 |
| Cook Islands | 0.00 | 12.05 | 0.00 | 0.05 | 14.67 | 0.00 | 65.97 | 0.00 | 7.25 | 0.00 |
| Costa Rica | 1.18 | 14.17 | 0.00 | 0.00 | 50.96 | 0.00 | 0.00 | 33.69 | 0.00 | 0.00 |
| Cuba | 0.00 | 0.00 | 14.33 | 0.00 | 0.00 | 0.00 | 0.00 | 0.00 | 0.00 | 85.67 |
| Democratic Republic of the Congo | 0.00 | 0.03 | 0.00 | 0.00 | 1.88 | 0.00 | 0.00 | 98.09 | 0.00 | 0.00 |
| Djibouti | 0.00 | 0.00 | 0.00 | 0.00 | 0.00 | 0.00 | 0.00 | 0.00 | 100.00 | 0.00 |
| Dominica | 0.00 | 0.00 | 0.44 | 0.00 | 57.55 | 1.35 | 1.48 | 2.91 | 36.27 | 0.00 |
| Dominican Republic | 0.00 | 13.48 | 0.00 | 0.00 | 12.82 | 0.00 | 36.76 | 31.06 | 5.88 | 0.00 |
| Ecuador | 0.00 | 0.00 | 0.00 | 0.00 | 0.00 | 0.00 | 98.08 | 0.00 | 1.92 | 0.00 |
| Egypt | 0.00 | 13.67 | 0.00 | 0.00 | 38.41 | 0.00 | 47.91 | 0.00 | 0.00 | 0.00 |
| El Salvador | 0.02 | 4.93 | 0.38 | 0.00 | 0.00 | 1.11 | 87.44 | 0.00 | 0.00 | 6.12 |
| Equatorial Guinea | 0.00 | 47.24 | 0.00 | 0.00 | 0.00 | 0.00 | 40.70 | 0.00 | 12.06 | 0.00 |
| Eritrea | 0.00 | 2.50 | 7.79 | 0.00 | 0.00 | 4.48 | 0.00 | 70.59 | 1.10 | 13.53 |
| Ethiopia | 0.00 | 0.57 | 0.00 | 0.00 | 0.00 | 0.00 | 99.43 | 0.00 | 0.00 | 0.00 |
| Fiji | 0.00 | 36.45 | 0.00 | 0.00 | 63.55 | 0.00 | 0.00 | 0.00 | 0.00 | 0.00 |
| Gabon | 0.00 | 36.45 | 1.53 | 0.00 | 30.47 | 0.00 | 31.55 | 0.00 | 0.00 | 0.00 |
| Gambia | 0.00 | 46.65 | 0.00 | 0.00 | 0.00 | 0.00 | 53.35 | 0.00 | 0.00 | 0.00 |
| Ghana | 0.00 | 33.38 | 0.00 | 0.00 | 0.00 | 0.00 | 66.62 | 0.00 | 0.00 | 0.00 |
| Grenada | 0.00 | 0.00 | 0.11 | 0.00 | 21.40 | 0.32 | 23.43 | 53.98 | 0.00 | 0.76 |
| Guatemala | 0.00 | 0.00 | 0.00 | 0.00 | 15.22 | 0.00 | 81.12 | 0.00 | 3.66 | 0.00 |
| Guinea | 0.00 | 0.00 | 0.00 | 0.00 | 7.68 | 0.00 | 0.00 | 0.00 | 92.32 | 0.00 |
| Guinea-Bissau | 0.00 | 0.00 | 6.77 | 0.00 | 0.00 | 0.00 | 93.23 | 0.00 | 0.00 | 0.00 |
| Guyana | 0.00 | 0.00 | 0.00 | 0.00 | 0.00 | 0.00 | 0.00 | 0.00 | 100.00 | 0.00 |
| Haiti |  |  |  |  |  |  |  |  |  |  |
| Honduras | 0.00 | 0.00 | 5.56 | 0.00 | 0.00 | 0.00 | 75.25 | 0.54 | 18.66 | 0.00 |
| India | 0.00 | 0.00 | 0.00 | 0.00 | 75.38 | 0.00 | 0.00 | 3.08 | 21.54 | 0.00 |
| Indonesia | 0.00 | 0.00 | 0.00 | 0.00 | 11.53 | 0.00 | 40.17 | 37.75 | 3.14 | 7.42 |
| Jamaica | 0.08 | 0.00 | 1.02 | 0.00 | 0.00 | 0.00 | 92.09 | 1.78 | 0.53 | 4.51 |
| Jordan | 0.00 | 0.00 | 0.00 | 0.00 | 14.97 | 0.00 | 85.03 | 0.00 | 0.00 | 0.00 |
| Kenya | 0.00 | 19.46 | 5.25 | 0.00 | 0.00 | 0.00 | 75.29 | 0.00 | 0.00 | 0.00 |
| Kuwait | 0.00 | 41.50 | 0.00 | 0.00 | 0.00 | 0.00 | 58.50 | 0.00 | 0.00 | 0.00 |
| Lao People's Democratic Republic | 0.00 | 0.00 | 0.00 | 0.00 | 0.00 | 0.00 | 100.00 | 0.00 | 0.00 | 0.00 |
| Lebanon | 0.00 | 41.06 | 0.00 | 0.00 | 24.73 | 0.00 | 34.21 | 0.00 | 0.00 | 0.00 |
| Liberia | 0.00 | 0.00 | 22.06 | 0.00 | 1.86 | 25.93 | 0.00 | 0.00 | 0.00 | 50.14 |
| Madagascar | 0.00 | 0.00 | 5.69 | 31.31 | 4.23 | 37.88 | 0.00 | 0.00 | 0.00 | 20.90 |
| Malawi | 0.00 | 0.54 | 0.00 | 0.00 | 32.58 | 0.00 | 58.73 | 0.00 | 0.00 | 8.15 |
| Malaysia | 0.00 | 0.00 | 0.00 | 0.00 | 25.58 | 0.00 | 64.77 | 9.64 | 0.00 | 0.00 |
| Maldives | 0.00 | 0.00 | 0.00 | 0.00 | 13.23 | 0.00 | 86.70 | 0.00 | 0.07 | 0.00 |
| Mali | 0.00 | 0.00 | 100.00 | 0.00 | 0.00 | 0.00 | 0.00 | 0.00 | 0.00 | 0.00 |
| Mauritania | 0.00 | 0.00 | 93.18 | 0.00 | 0.00 | 6.82 | 0.00 | 0.00 | 0.00 | 0.00 |
| Mauritius | 0.08 | 73.40 | 0.24 | 0.00 | 0.00 | 3.20 | 0.00 | 14.12 | 6.95 | 2.02 |
| Mexico | 1.42 | 14.60 | 0.00 | 0.00 | 75.61 | 0.00 | 0.00 | 8.37 | 0.00 | 0.00 |
| Micronesia (Federated States of) | 0.00 | 0.00 | 0.00 | 0.00 | 11.95 | 0.00 | 85.35 | 2.41 | 0.29 | 0.00 |
| Mozambique | 0.00 | 8.08 | 23.57 | 1.14 | 0.00 | 45.00 | 0.00 | 6.96 | 2.76 | 12.49 |
| Myanmar | 0.00 | 16.00 | 1.27 | 0.94 | 24.52 | 12.96 | 7.91 | 0.00 | 23.94 | 12.45 |
| Nepal | 0.00 | 0.00 | 34.50 | 0.00 | 0.00 | 49.95 | 0.00 | 0.00 | 15.55 | 0.00 |
| Nicaragua | 0.00 | 0.00 | 0.00 | 0.00 | 30.52 | 0.00 | 69.48 | 0.00 | 0.00 | 0.00 |
| Niger | 0.00 | 0.00 | 0.77 | 0.00 | 0.00 | 0.00 | 79.02 | 13.29 | 6.92 | 0.01 |
| Nigeria | 0.00 | 62.49 | 0.00 | 0.00 | 1.36 | 0.00 | 22.77 | 0.00 | 13.38 | 0.00 |
| Niue | 0.00 | 0.00 | 0.00 | 0.00 | 0.00 | 0.00 | 79.91 | 0.00 | 20.09 | 0.00 |
| Oman | 0.00 | 1.25 | 0.00 | 0.00 | 1.23 | 2.43 | 94.97 | 0.00 | 0.12 | 0.00 |
| Pakistan | 42.00 | 35.92 | 0.94 | 0.00 | 0.00 | 0.00 | 2.41 | 14.99 | 0.00 | 3.74 |
| Panama | 0.00 | 21.97 | 0.00 | 0.00 | 12.88 | 0.00 | 65.15 | 0.00 | 0.00 | 0.00 |
| Papua New Guinea | 0.00 | 0.00 | 0.00 | 0.00 | 0.00 | 0.00 | 72.60 | 0.00 | 21.71 | 5.69 |
| Paraguay | 0.00 | 16.93 | 0.00 | 0.00 | 56.29 | 0.00 | 26.12 | 0.66 | 0.00 | 0.00 |
| Peru | 0.00 | 4.69 | 0.00 | 0.00 | 44.24 | 0.00 | 44.49 | 3.35 | 3.24 | 0.00 |
| Philippines | 0.00 | 0.00 | 0.00 | 0.00 | 4.33 | 0.00 | 88.26 | 0.00 | 7.41 | 0.00 |
| Rwanda | 0.18 | 0.00 | 3.79 | 0.00 | 44.02 | 0.00 | 0.00 | 52.01 | 0.00 | 0.00 |
| Saint Kitts and Nevis | 0.00 | 0.00 | 0.00 | 0.00 | 63.96 | 0.00 | 34.36 | 0.00 | 1.68 | 0.00 |
| Saint Lucia | 0.00 | 0.00 | 0.00 | 0.00 | 0.00 | 0.00 | 0.00 | 100.00 | 0.00 | 0.00 |
| Saint Vincent and the Grenadines | 0.00 | 0.00 | 0.00 | 0.00 | 0.00 | 56.22 | 0.00 | 43.78 | 0.00 | 0.00 |
| Sao Tome and Principe | 0.00 | 8.72 | 3.41 | 0.00 | 11.46 | 0.31 | 0.00 | 68.66 | 0.00 | 7.44 |
| Saudi Arabia | 0.00 | 0.00 | 0.00 | 0.00 | 1.43 | 6.51 | 92.05 | 0.00 | 0.00 | 0.00 |
| Senegal | 0.09 | 78.90 | 1.78 | 0.00 | 0.00 | 3.71 | 3.55 | 0.00 | 0.00 | 11.98 |
| Seychelles | 0.00 | 0.00 | 0.00 | 0.28 | 0.00 | 0.00 | 32.18 | 66.35 | 1.19 | 0.00 |
| Sierra Leone | 0.00 | 17.14 | 0.00 | 0.00 | 73.46 | 0.00 | 0.00 | 0.00 | 9.40 | 0.00 |
| Singapore | 2.44 | 0.00 | 0.00 | 0.00 | 4.89 | 92.67 | 0.00 | 0.00 | 0.00 | 0.00 |
| Somalia | 0.00 | 79.13 | 0.00 | 11.87 | 0.32 | 0.00 | 0.00 | 3.96 | 0.00 | 4.72 |
| South Sudan | 0.00 | 7.11 | 0.00 | 0.00 | 11.21 | 0.00 | 81.69 | 0.00 | 0.00 | 0.00 |
| Sri Lanka | 0.00 | 0.00 | 0.01 | 0.00 | 1.88 | 0.50 | 7.05 | 90.25 | 0.31 | 0.00 |
| Sudan | 0.00 | 7.63 | 0.00 | 0.00 | 5.94 | 0.00 | 83.68 | 0.00 | 2.75 | 0.00 |
| Suriname | 0.00 | 4.24 | 0.00 | 0.00 | 12.29 | 0.00 | 82.65 | 0.00 | 0.00 | 0.82 |
| Syrian Arab Republic | 0.00 | 0.00 | 30.33 | 0.00 | 1.75 | 0.00 | 0.00 | 0.00 | 0.00 | 67.92 |
| Thailand | 0.00 | 0.26 | 0.00 | 0.00 | 45.69 | 0.00 | 44.56 | 9.49 | 0.00 | 0.00 |
| Timor-Leste | 0.00 | 0.00 | 0.00 | 0.00 | 62.49 | 0.00 | 37.51 | 0.00 | 0.00 | 0.00 |
| Togo | 0.00 | 5.48 | 11.11 | 5.29 | 0.14 | 28.00 | 0.00 | 40.39 | 0.00 | 9.59 |
| Trinidad and Tobago | 0.00 | 10.33 | 0.00 | 0.00 | 0.00 | 0.00 | 85.13 | 0.00 | 4.54 | 0.00 |
| Uganda | 0.00 | 0.00 | 0.00 | 0.00 | 0.00 | 0.00 | 100.00 | 0.00 | 0.00 | 0.00 |
| United Republic of Tanzania | 0.00 | 0.00 | 0.00 | 0.00 | 27.91 | 0.00 | 0.00 | 68.79 | 3.29 | 0.00 |
| United States of America | 0.00 | 1.22 | 0.00 | 0.00 | 8.77 | 0.00 | 0.00 | 90.01 | 0.00 | 0.00 |
| Venezuela (Bolivarian Republic of) | 0.00 | 3.08 | 0.00 | 96.92 | 0.00 | 0.00 | 0.00 | 0.00 | 0.00 | 0.00 |
| Viet Nam | 0.00 | 0.00 | 0.00 | 0.00 | 0.00 | 0.00 | 100.00 | 0.00 | 0.00 | 0.00 |
| Yemen | 0.00 | 55.78 | 0.00 | 0.00 | 0.00 | 0.00 | 44.22 | 0.00 | 0.00 | 0.00 |
| Zambia | 0.00 | 69.48 | 0.00 | 0.00 | 30.52 | 0.00 | 0.00 | 0.00 | 0.00 | 0.00 |
| Zimbabwe | 0.00 | 0.00 | 0.00 | 0.00 | 13.32 | 0.00 | 15.90 | 0.00 | 12.29 | 58.48 |

*x20: Annual freshwater withdrawals, total (billion cubic meters); x3: Temperature anomaly; x19: Urban population (% of total population); x13: Population, total; x10: Life expectancy at birth (years); x17: Average number of years adults aged 25+ spent in formal education; x16: Access to electricity; x9: Domestic general government health expenditure (%); x12: UHC Service Coverage Index (SDG 3.8.1); x14: GDP growth (annual %).*

**Table S18.** Country wise SHAP feature contributions (%) to predicted dengue incidence (female).

| **Country** | **x7** | **x20** | **x14** | **x12** | **x11** | **x8** | **x19** | **x9** | **x17** | **x16** |
| --- | --- | --- | --- | --- | --- | --- | --- | --- | --- | --- |
| Afghanistan | 2.01 | 0.00 | 0.00 | 0.00 | 0.00 | 35.42 | 0.00 | 0.48 | 14.03 | 48.05 |
| Angola | 2.07 | 0.00 | 0.00 | 1.92 | 0.00 | 56.37 | 0.00 | 5.88 | 7.91 | 25.84 |
| Antigua and Barbuda | 0.00 | 0.00 | 0.00 | 0.00 | 0.00 | 0.00 | 0.00 | 99.38 | 0.62 | 0.00 |
| Argentina | 0.04 | 0.00 | 0.00 | 0.00 | 0.00 | 24.96 | 0.00 | 0.00 | 0.72 | 74.29 |
| Australia | 0.07 | 2.21 | 0.00 | 0.00 | 0.00 | 1.31 | 0.00 | 0.00 | 0.85 | 95.56 |
| Bahamas | 0.00 | 0.00 | 0.00 | 0.00 | 0.00 | 0.00 | 0.00 | 0.00 | 8.80 | 91.20 |
| Bangladesh | 2.05 | 0.00 | 0.00 | 8.33 | 0.00 | 0.00 | 0.00 | 0.00 | 0.00 | 89.62 |
| Barbados | 0.16 | 0.00 | 0.00 | 2.34 | 0.00 | 0.00 | 0.00 | 0.00 | 7.81 | 89.69 |
| Belize | 0.05 | 0.00 | 0.04 | 0.00 | 0.00 | 78.28 | 0.00 | 0.00 | 0.00 | 21.63 |
| Benin | 1.76 | 0.00 | 0.00 | 0.00 | 1.46 | 30.59 | 0.00 | 0.04 | 22.80 | 43.36 |
| Bhutan | 0.17 | 0.00 | 0.00 | 27.64 | 0.00 | 62.28 | 9.91 | 0.00 | 0.00 | 0.00 |
| Bolivia (Plurinational State of) | 0.00 | 0.00 | 0.00 | 3.46 | 0.00 | 0.00 | 0.00 | 51.54 | 0.00 | 45.00 |
| Brazil | 0.53 | 0.34 | 0.02 | 0.00 | 0.00 | 9.00 | 0.00 | 1.82 | 6.55 | 81.74 |
| Brunei Darussalam | 0.03 | 0.00 | 0.00 | 2.00 | 0.00 | 18.98 | 0.00 | 78.99 | 0.00 | 0.00 |
| Burkina Faso | 1.01 | 0.00 | 0.72 | 2.25 | 0.00 | 0.00 | 0.00 | 4.80 | 14.80 | 76.43 |
| Burundi | 0.73 | 0.00 | 0.00 | 0.00 | 0.00 | 5.47 | 0.00 | 0.00 | 18.06 | 75.73 |
| Cabo Verde | 0.01 | 0.01 | 0.00 | 0.64 | 0.84 | 44.32 | 2.45 | 51.73 | 0.00 | 0.00 |
| Cambodia | 0.00 | 0.00 | 0.00 | 0.92 | 49.36 | 49.72 | 0.00 | 0.00 | 0.00 | 0.00 |
| Cameroon | 1.47 | 0.00 | 0.00 | 0.00 | 0.00 | 21.68 | 0.00 | 0.00 | 8.38 | 68.46 |
| Central African Republic | 0.07 | 0.00 | 0.00 | 0.00 | 0.00 | 0.00 | 0.00 | 0.00 | 1.09 | 98.84 |
| Chad | 1.41 | 0.00 | 1.70 | 2.01 | 0.00 | 0.00 | 0.00 | 0.00 | 9.06 | 85.82 |
| China | 0.00 | 0.25 | 0.00 | 0.00 | 0.00 | 5.83 | 0.00 | 0.00 | 1.11 | 92.81 |
| Colombia | 0.34 | 1.44 | 0.00 | 0.00 | 0.00 | 53.41 | 0.00 | 0.00 | 3.61 | 41.20 |
| Comoros | 0.04 | 0.00 | 0.00 | 2.37 | 0.00 | 2.70 | 0.00 | 0.00 | 10.88 | 84.02 |
| Cook Islands | 3.22 | 0.00 | 0.00 | 0.00 | 0.00 | 0.00 | 0.00 | 0.00 | 0.00 | 96.78 |
| Costa Rica | 0.02 | 0.26 | 0.00 | 0.00 | 0.00 | 24.46 | 0.00 | 3.00 | 0.62 | 71.65 |
| Cuba | 0.23 | 0.00 | 0.00 | 0.00 | 56.42 | 28.29 | 7.62 | 0.00 | 7.44 | 0.00 |
| Democratic Republic of the Congo | 0.07 | 0.00 | 0.00 | 0.00 | 0.00 | 0.00 | 0.00 | 99.93 | 0.00 | 0.00 |
| Djibouti | 0.00 | 0.00 | 0.00 | 38.94 | 0.00 | 0.00 | 0.00 | 0.00 | 61.06 | 0.00 |
| Dominica | 0.00 | 0.00 | 0.00 | 0.61 | 0.00 | 0.00 | 9.79 | 14.03 | 0.00 | 75.57 |
| Dominican Republic | 0.16 | 0.00 | 1.03 | 0.00 | 0.28 | 61.51 | 0.00 | 27.31 | 9.70 | 0.00 |
| Ecuador | 0.14 | 0.00 | 0.00 | 0.10 | 0.00 | 49.95 | 0.00 | 0.00 | 5.19 | 44.63 |
| Egypt | 0.00 | 0.00 | 0.00 | 0.08 | 0.00 | 0.00 | 0.00 | 0.00 | 8.70 | 91.22 |
| El Salvador | 0.00 | 0.01 | 0.03 | 0.00 | 1.71 | 0.00 | 5.66 | 0.00 | 0.86 | 91.72 |
| Equatorial Guinea | 1.07 | 0.00 | 0.00 | 15.13 | 0.00 | 27.37 | 0.00 | 0.00 | 56.42 | 0.00 |
| Eritrea | 0.00 | 0.00 | 0.00 | 0.13 | 3.13 | 0.00 | 7.82 | 88.31 | 0.62 | 0.00 |
| Ethiopia | 19.17 | 0.00 | 0.00 | 0.00 | 0.00 | 0.00 | 0.00 | 0.00 | 0.96 | 79.87 |
| Fiji | 0.01 | 0.00 | 0.35 | 0.00 | 0.00 | 99.64 | 0.00 | 0.00 | 0.00 | 0.00 |
| Gabon | 0.00 | 0.00 | 0.00 | 1.70 | 0.00 | 28.07 | 0.00 | 0.00 | 0.00 | 70.23 |
| Gambia | 0.00 | 0.00 | 0.00 | 0.54 | 0.00 | 0.00 | 0.00 | 0.00 | 6.50 | 92.96 |
| Ghana | 1.14 | 0.00 | 0.00 | 0.00 | 0.00 | 0.00 | 0.00 | 0.00 | 15.19 | 83.67 |
| Grenada | 0.00 | 0.00 | 0.00 | 1.85 | 0.00 | 0.00 | 1.36 | 96.79 | 0.00 | 0.00 |
| Guatemala | 0.28 | 0.00 | 0.00 | 0.00 | 0.00 | 8.83 | 0.00 | 0.31 | 13.59 | 76.99 |
| Guinea | 4.21 | 0.00 | 4.57 | 0.00 | 0.00 | 0.00 | 0.00 | 0.00 | 91.22 | 0.00 |
| Guinea-Bissau | 0.00 | 0.00 | 0.12 | 0.00 | 0.00 | 93.71 | 0.00 | 0.00 | 0.62 | 5.55 |
| Guyana | 0.34 | 0.00 | 0.02 | 5.44 | 0.00 | 0.00 | 0.00 | 38.25 | 55.94 | 0.00 |
| Haiti | 4.79 | 0.00 | 27.91 | 0.00 | 0.00 | 67.31 | 0.00 | 0.00 | 0.00 | 0.00 |
| Honduras | 0.68 | 0.00 | 0.00 | 0.20 | 0.00 | 62.77 | 0.00 | 0.00 | 31.10 | 5.25 |
| India | 0.03 | 0.00 | 0.00 | 0.00 | 0.00 | 0.00 | 0.00 | 0.00 | 2.62 | 97.35 |
| Indonesia | 2.35 | 0.00 | 0.00 | 1.84 | 7.47 | 0.00 | 0.00 | 24.70 | 11.34 | 52.29 |
| Jamaica | 0.00 | 0.45 | 0.00 | 0.00 | 29.45 | 0.00 | 25.53 | 10.97 | 0.00 | 33.59 |
| Jordan | 0.08 | 0.02 | 0.00 | 8.11 | 3.99 | 16.05 | 0.00 | 0.00 | 0.00 | 71.75 |
| Kenya | 1.55 | 0.00 | 0.00 | 0.00 | 0.00 | 14.18 | 0.00 | 19.31 | 10.38 | 54.58 |
| Kuwait | 0.07 | 0.00 | 0.00 | 0.86 | 0.00 | 36.90 | 0.00 | 0.00 | 12.64 | 49.53 |
| Lao People's Democratic Republic | 0.00 | 0.00 | 0.00 | 0.00 | 0.00 | 0.00 | 0.00 | 0.00 | 0.00 | 100.00 |
| Lebanon | 0.00 | 0.00 | 0.01 | 1.46 | 0.00 | 0.00 | 0.00 | 0.00 | 0.00 | 98.54 |
| Liberia | 0.00 | 0.00 | 0.00 | 0.04 | 1.67 | 0.00 | 6.92 | 31.12 | 0.00 | 60.24 |
| Madagascar | 0.15 | 0.00 | 9.80 | 9.92 | 4.04 | 0.00 | 3.27 | 0.00 | 0.15 | 72.66 |
| Malawi | 100.00 | 0.00 | 0.00 | 0.00 | 0.00 | 0.00 | 0.00 | 0.00 | 0.00 | 0.00 |
| Malaysia | 3.30 | 0.00 | 0.00 | 3.17 | 0.00 | 0.00 | 0.00 | 30.13 | 63.40 | 0.00 |
| Maldives | 0.00 | 0.00 | 0.00 | 0.24 | 0.26 | 31.08 | 0.24 | 68.04 | 0.15 | 0.00 |
| Mali | 2.22 | 0.00 | 15.99 | 0.00 | 0.00 | 0.00 | 0.00 | 0.00 | 29.38 | 52.40 |
| Mauritania | 0.00 | 0.00 | 0.00 | 8.29 | 16.02 | 52.94 | 0.00 | 0.00 | 0.80 | 21.94 |
| Mauritius | 0.00 | 0.15 | 0.00 | 0.15 | 0.00 | 31.35 | 4.37 | 28.23 | 35.75 | 0.00 |
| Mexico | 19.45 | 40.80 | 0.00 | 0.00 | 0.00 | 0.00 | 0.00 | 7.40 | 32.35 | 0.00 |
| Micronesia (Federated States of) | 0.00 | 0.00 | 0.00 | 0.00 | 0.00 | 0.00 | 0.00 | 5.13 | 0.00 | 94.87 |
| Mozambique | 0.00 | 0.00 | 0.00 | 4.96 | 27.60 | 31.68 | 34.75 | 1.01 | 0.00 | 0.00 |
| Myanmar | 0.04 | 0.00 | 0.00 | 15.50 | 21.55 | 31.27 | 4.60 | 27.05 | 0.00 | 0.00 |
| Nepal | 0.00 | 0.00 | 2.65 | 17.43 | 21.22 | 45.97 | 12.73 | 0.00 | 0.00 | 0.00 |
| Nicaragua | 0.00 | 0.00 | 0.00 | 0.65 | 0.00 | 33.14 | 0.00 | 0.62 | 3.44 | 62.15 |
| Niger | 0.91 | 0.01 | 0.22 | 0.00 | 0.12 | 0.00 | 0.00 | 0.00 | 3.14 | 95.60 |
| Nigeria | 26.35 | 0.00 | 0.00 | 10.54 | 0.00 | 3.70 | 0.00 | 0.00 | 59.41 | 0.00 |
| Niue | 0.00 | 0.00 | 0.00 | 0.00 | 0.00 | 0.00 | 0.00 | 0.00 | 0.00 | 100.00 |
| Oman | 0.03 | 0.00 | 0.00 | 0.00 | 0.00 | 0.00 | 13.58 | 0.26 | 0.00 | 86.13 |
| Pakistan | 0.00 | 0.00 | 0.00 | 42.10 | 57.90 | 0.00 | 0.00 | 0.00 | 0.00 | 0.00 |
| Panama | 0.05 | 0.00 | 0.00 | 0.00 | 0.00 | 95.89 | 0.00 | 0.00 | 4.06 | 0.00 |
| Papua New Guinea | 0.40 | 0.00 | 0.00 | 0.00 | 0.00 | 0.00 | 0.00 | 1.41 | 0.98 | 97.21 |
| Paraguay | 0.22 | 0.00 | 0.00 | 4.23 | 0.00 | 30.95 | 0.00 | 0.00 | 10.42 | 54.18 |
| Peru | 0.49 | 0.00 | 0.67 | 2.45 | 0.00 | 88.65 | 0.00 | 1.27 | 6.48 | 0.00 |
| Philippines | 0.74 | 0.00 | 0.00 | 0.50 | 0.00 | 0.22 | 0.00 | 0.00 | 11.51 | 87.03 |
| Rwanda | 1.42 | 0.41 | 0.00 | 9.50 | 0.00 | 0.00 | 0.00 | 0.00 | 88.67 | 0.00 |
| Saint Kitts and Nevis | 0.00 | 0.00 | 0.00 | 0.69 | 0.00 | 0.00 | 0.00 | 0.00 | 0.00 | 99.31 |
| Saint Lucia | 0.00 | 0.00 | 0.00 | 5.18 | 0.00 | 0.00 | 0.00 | 0.00 | 94.82 | 0.00 |
| Saint Vincent and the Grenadines | 0.00 | 0.00 | 0.00 | 19.64 | 0.00 | 0.00 | 0.00 | 0.00 | 80.36 | 0.00 |
| Sao Tome and Principe | 0.01 | 0.00 | 0.00 | 2.95 | 0.42 | 4.81 | 2.54 | 67.23 | 0.00 | 22.03 |
| Saudi Arabia | 0.00 | 0.00 | 0.00 | 0.60 | 0.00 | 0.00 | 0.81 | 0.00 | 0.00 | 98.59 |
| Senegal | 1.88 | 0.00 | 0.00 | 0.00 | 0.00 | 3.33 | 0.00 | 0.00 | 0.00 | 94.79 |
| Seychelles | 0.09 | 0.00 | 0.00 | 0.00 | 0.00 | 0.00 | 0.00 | 0.00 | 1.91 | 98.01 |
| Sierra Leone | 1.02 | 0.00 | 14.59 | 4.67 | 0.00 | 0.00 | 0.00 | 0.00 | 7.35 | 72.37 |
| Singapore | 25.49 | 43.26 | 0.08 | 0.08 | 31.00 | 0.00 | 0.08 | 0.00 | 0.00 | 0.00 |
| Somalia | 1.35 | 0.00 | 4.35 | 0.00 | 0.00 | 24.64 | 0.00 | 25.99 | 0.00 | 43.67 |
| South Sudan | 0.27 | 0.00 | 0.05 | 1.25 | 0.00 | 0.00 | 0.00 | 0.00 | 0.00 | 98.44 |
| Sri Lanka | 0.01 | 0.00 | 0.09 | 0.00 | 0.24 | 0.00 | 0.00 | 99.47 | 0.19 | 0.00 |
| Sudan | 0.90 | 0.00 | 0.00 | 0.09 | 0.00 | 0.00 | 0.00 | 0.00 | 4.04 | 94.97 |
| Suriname | 0.06 | 0.00 | 0.00 | 45.84 | 0.00 | 0.00 | 0.00 | 0.00 | 3.95 | 50.15 |
| Syrian Arab Republic | 0.00 | 0.00 | 0.00 | 0.00 | 0.00 | 14.23 | 85.77 | 0.00 | 0.00 | 0.00 |
| Thailand | 0.05 | 0.00 | 0.02 | 0.00 | 0.00 | 0.00 | 0.00 | 4.85 | 3.09 | 91.98 |
| Timor-Leste | 0.03 | 0.00 | 2.58 | 0.00 | 0.00 | 63.82 | 0.00 | 0.00 | 0.00 | 33.56 |
| Togo | 0.00 | 0.00 | 0.00 | 6.46 | 7.01 | 0.00 | 8.58 | 77.94 | 0.00 | 0.00 |
| Trinidad and Tobago | 0.00 | 0.00 | 0.00 | 0.95 | 0.00 | 3.17 | 0.00 | 0.00 | 1.11 | 94.77 |
| Uganda | 30.24 | 0.00 | 0.00 | 10.32 | 0.00 | 0.00 | 0.00 | 0.00 | 59.44 | 0.00 |
| United Republic of Tanzania | 0.00 | 0.00 | 0.00 | 28.64 | 0.00 | 0.00 | 0.00 | 71.36 | 0.00 | 0.00 |
| United States of America | 0.00 | 0.00 | 0.00 | 0.00 | 0.00 | 0.00 | 0.00 | 100.00 | 0.00 | 0.00 |
| Venezuela (Bolivarian Republic of) | 0.00 | 0.00 | 0.00 | 1.75 | 0.00 | 0.00 | 0.00 | 0.00 | 0.00 | 98.25 |
| Viet Nam | 4.90 | 0.00 | 0.44 | 2.71 | 0.00 | 0.00 | 0.00 | 0.00 | 0.00 | 91.95 |
| Yemen | 0.00 | 0.00 | 0.00 | 0.28 | 0.00 | 0.00 | 0.00 | 0.00 | 5.51 | 94.21 |
| Zambia | 1.55 | 0.00 | 0.00 | 1.89 | 0.00 | 15.44 | 0.00 | 0.00 | 14.13 | 66.99 |
| Zimbabwe | 0.12 | 0.91 | 10.63 | 0.00 | 5.40 | 18.30 | 0.00 | 0.00 | 17.13 | 47.52 |

*x7: Hospital bed density (per 10,000 population); x20: Annual freshwater withdrawals, total (billion cubic meters); x14: GDP growth (annual %); x12: UHC Service Coverage Index (SDG 3.8.1); x11: Mortality rate under 5 per 1000 live births; x8: Density of physicians (per 10,000 population); x19: Urban population (% of total population); x9: Domestic general government health expenditure (%); x17: Average number of years adults aged 25+ spent in formal education; x16: Access to electricity.*

**Table S19.** Sensitivity analysis of top 10 features on predicted dengue incidence (Both)

| **Change** | **x20** | **x3** | **x19** | **x13** | **x10** | **x17** | **x16** | **x9** | **x12** | **x14** |
| --- | --- | --- | --- | --- | --- | --- | --- | --- | --- | --- |
| -50 | -65.03 | -0.15 | -34.68 | 11.35 | -1.99 | 5.27 | -0.81 | -2.40 | -2.77 | -2.36 |
| -40 | -59.13 | -0.12 | -33.74 | 8.91 | -1.81 | 5.53 | -0.80 | -3.22 | -3.05 | -2.08 |
| -30 | -53.76 | -0.09 | -35.39 | 5.44 | -1.87 | 4.68 | -0.73 | -2.15 | -3.21 | -1.82 |
| -20 | -31.87 | -0.07 | -35.47 | 3.46 | -1.86 | 2.28 | -0.45 | -1.35 | -3.37 | -1.66 |
| -10 | -28.80 | -0.04 | -33.93 | 0.84 | -1.56 | 1.08 | -0.32 | -1.52 | -3.12 | -0.52 |
| 0 | 0.00 | 0.00 | 0.00 | 0.00 | 0.00 | 0.00 | 0.00 | 0.00 | 0.00 | 0.00 |
| 10 | 36.20 | 0.03 | 52.86 | -1.88 | 0.46 | -0.80 | 0.92 | 0.05 | 2.95 | -0.22 |
| 20 | 48.93 | 0.04 | 67.22 | -3.06 | 0.62 | -1.35 | 1.72 | 0.16 | 5.41 | -0.08 |
| 30 | 72.22 | 0.04 | 69.24 | -3.61 | 1.06 | -1.65 | 2.39 | 0.16 | 6.38 | 0.09 |
| 40 | 81.43 | 0.04 | 74.46 | -4.64 | 1.32 | -1.80 | 2.83 | 0.18 | 8.02 | 0.22 |
| 50 | 91.45 | 0.06 | 85.66 | -5.25 | 1.49 | -1.93 | 3.72 | 0.25 | 8.07 | 0.44 |

*x20: Annual freshwater withdrawals, total (billion cubic meters); x3: Temperature anomaly; x19: Urban population (% of total population); x13: Population, total; x10: Life expectancy at birth (years); x17: Average number of years adults aged 25+ spent in formal education; x16: Access to electricity; x9: Domestic general government health expenditure (%); x12: UHC Service Coverage Index (SDG 3.8.1); x14: GDP growth (annual %).*

**Table S20.** Sensitivity analysis of top 10 features on predicted dengue incidence (male)

| **Change** | **x20** | **x7** | **x14** | **x19** | **x9** | **x17** | **x5** | **x10** | **x8** | **x12** |
| --- | --- | --- | --- | --- | --- | --- | --- | --- | --- | --- |
| -50 | -57.17 | -24.54 | -5.31 | -22.90 | -3.15 | -0.68 | 6.03 | -1.45 | 0.00 | -5.63 |
| -40 | -53.31 | -23.07 | -4.87 | -22.81 | -1.80 | -0.55 | 3.69 | -1.29 | -0.03 | -5.62 |
| -30 | -49.59 | -21.62 | -4.07 | -23.26 | -0.78 | -0.46 | 1.94 | -1.40 | -0.05 | -5.59 |
| -20 | -33.56 | -20.65 | -3.72 | -23.29 | -0.63 | -0.37 | 1.08 | -1.36 | -0.03 | -5.42 |
| -10 | -29.52 | -15.83 | -2.20 | -22.33 | 0.15 | -0.23 | 0.44 | -1.01 | -0.02 | -2.73 |
| 0 | 0.00 | 0.00 | 0.00 | 0.00 | 0.00 | 0.00 | 0.00 | 0.00 | 0.00 | 0.00 |
| 10 | 32.86 | 13.04 | 2.98 | 24.09 | -0.59 | 0.12 | -0.98 | 1.68 | 0.26 | 2.44 |
| 20 | 38.48 | 19.52 | 4.76 | 28.80 | -0.39 | 0.21 | -1.70 | 1.95 | 0.28 | 3.74 |
| 30 | 52.59 | 22.17 | 5.68 | 32.75 | -0.27 | 0.28 | -1.86 | 2.09 | 0.33 | 5.17 |
| 40 | 58.49 | 23.08 | 6.59 | 40.01 | 0.23 | 0.40 | -1.43 | 2.19 | 0.39 | 6.68 |
| 50 | 65.45 | 23.91 | 6.91 | 50.71 | 0.06 | 0.42 | -2.41 | 2.45 | 0.46 | 7.98 |

*x20: Annual freshwater withdrawals, total (billion cubic meters); x7: Hospital bed density (per 10,000 population); x14: GDP growth (annual %); x19: Urban population (% of total population); x9: Domestic general government health expenditure (%); x17: Average number of years adults aged 25+ spent in formal education; x5: Agricultural land (% of land area); x10: Life expectancy at birth (years); x8: Density of physicians (per 10,000 population); x12: UHC Service Coverage Index (SDG 3.8.1).*

**Table S21.** Sensitivity Analysis of Top 10 Features on Predicted Dengue Incidence (female)

| Change | x7 | x20 | x14 | x12 | x11 | x8 | x19 | x9 | x17 | x16 |
| --- | --- | --- | --- | --- | --- | --- | --- | --- | --- | --- |
| -50 | -28.69 | -55.96 | -3.01 | -5.62 | 13.83 | -0.14 | -25.16 | -3.22 | 0.37 | -0.45 |
| -40 | -27.93 | -50.79 | -2.79 | -5.59 | 13.32 | -0.17 | -25.12 | -1.89 | 0.33 | -0.45 |
| -30 | -26.60 | -46.26 | -2.47 | -5.53 | 3.83 | 0.02 | -25.08 | -1.06 | 0.23 | -0.45 |
| -20 | -25.52 | -29.83 | -2.35 | -5.25 | 2.11 | 0.02 | -24.98 | -0.33 | 0.05 | -0.45 |
| -10 | -19.55 | -25.56 | -1.39 | -2.40 | 0.41 | 0.00 | -21.91 | 0.26 | -0.02 | -0.45 |
| 0 | 0.00 | 0.00 | 0.00 | 0.00 | 0.00 | 0.00 | 0.00 | 0.00 | 0.00 | 0.00 |
| 10 | 15.12 | 31.44 | 1.04 | 2.50 | 6.01 | -0.01 | 19.54 | -0.24 | 0.11 | 1.00 |
| 20 | 22.62 | 37.73 | 2.10 | 3.57 | 4.40 | -0.04 | 23.45 | 0.02 | 0.17 | 3.17 |
| 30 | 25.66 | 49.11 | 2.66 | 4.83 | 4.43 | -0.05 | 27.36 | -0.04 | 0.19 | 4.29 |
| 40 | 27.03 | 54.43 | 3.27 | 6.19 | 3.91 | -0.06 | 35.32 | 0.37 | 0.24 | 4.78 |
| 50 | 28.42 | 60.84 | 3.45 | 7.39 | 5.38 | -0.05 | 48.10 | 0.27 | 0.27 | 6.04 |

*x7: Hospital bed density (per 10,000 population); x20: Annual freshwater withdrawals, total (billion cubic meters); x14: GDP growth (annual %); x12: UHC Service Coverage Index (SDG 3.8.1); x11: Mortality rate under 5 per 1000 live births; x8: Density of physicians (per 10,000 population); x19: Urban population (% of total population); x9: Domestic general government health expenditure (%); x17: Average number of years adults aged 25+ spent in formal education; x16: Access to electricity.*

**Table S22.** Top features ranked by signed Information Gain (IG) percentage for the overall, male, and female models

| **Feature** | **IG_Percentage Signed for Both** | **Feature** | **IG_Percentage_Signed for mele** | **Feature** | **IG_Percentage_Signed for female** |
| --- | --- | --- | --- | --- | --- |
| x3 | -27.237 | x3 | -36.982 | x3 | -24.860 |
| x12 | -21.373 | x12 | -19.540 | x16 | -15.331 |
| x16 | -14.559 | x16 | -11.954 | x17 | -10.818 |
| x17 | -9.645 | x17 | -8.289 | x13 | 10.404 |
| x14 | -7.638 | x13 | 7.735 | x12 | -9.837 |
| x13 | 6.157 | x14 | -6.723 | x14 | -8.545 |
| x19 | -5.397 | x19 | -3.819 | x11 | 6.701 |
| x11 | 1.379 | x9 | 2.280 | x19 | -4.900 |
| x5 | -1.366 | x11 | 1.258 | x9 | 3.665 |
| x10 | -1.289 | x8 | 0.442 | x5 | -1.927 |

**Table S23.** Layer-wise Relevance Propagation (LRP) scores of input features across network layers for overall, male, and female models

| **Feature** | **LRP_Signed both** | **Feature** | **LRP_Signed male** | **Feature** | **LRP_Signed female** |
| --- | --- | --- | --- | --- | --- |
| **Input_Layer** |  |  |  |  |  |
| x3 | 1.757 | x3 | -15.766 | x3 | 0.212 |
| x5 | 1.028 | x5 | 27.295 | x5 | -2.692 |
| x7 | 7.293 | x7 | 1.130 | x7 | 10.714 |
| x8 | 1.500 | x8 | -0.366 | x8 | -28.350 |
| x9 | -15.843 | x9 | 8.895 | x9 | -11.447 |
| x10 | 9.991 | x10 | 0.699 | x10 | 15.061 |
| x11 | 8.670 | x11 | -0.482 | x11 | -2.199 |
| x12 | 11.482 | x12 | -17.826 | x12 | 1.494 |
| x13 | -11.186 | x13 | 3.149 | x13 | 2.402 |
| x14 | -3.541 | x14 | 12.757 | x14 | -2.378 |
| x16 | 5.763 | x16 | 8.394 | x16 | 1.831 |
| x17 | -7.289 | x17 | -1.949 | x17 | 20.344 |
| x19 | -13.504 | x19 | 0.665 | x19 | -0.357 |
| x20 | 1.152 | x20 | -0.627 | x20 | -0.518 |
| Hidden_Layer |  |  |  |  |  |
| x3 | 7.226 | x3 | 8.411 | x3 | 6.745 |
| x5 | 7.136 | x5 | 6.933 | x5 | 6.870 |
| x7 | 7.149 | x7 | 6.326 | x7 | 7.268 |
| x8 | 7.317 | x8 | 5.354 | x8 | 7.192 |
| x9 | 7.121 | x9 | 6.146 | x9 | 7.593 |
| x10 | 7.175 | x10 | 7.096 | x10 | 7.277 |
| x11 | 7.188 | x11 | 6.273 | x11 | 7.332 |
| x12 | 7.232 | x12 | 7.220 | x12 | 6.887 |
| x13 | 7.108 | x13 | 8.288 | x13 | 6.984 |
| x14 | 7.114 | x14 | 8.109 | x14 | 7.176 |
| x16 | 6.948 | x16 | 7.165 | x16 | 7.157 |
| x17 | 6.970 | x17 | 8.115 | x17 | 7.191 |
| x19 | 7.136 | x19 | 7.497 | x19 | 7.171 |
| x20 | 7.181 | x20 | 7.068 | x20 | 7.158 |
| FC_Layer |  |  |  |  |  |
| x3 | 7.303 | x3 | -5.108 | x3 | 7.129 |
| x5 | 7.219 | x5 | -5.854 | x5 | 7.156 |
| x7 | 7.234 | x7 | -6.218 | x7 | 7.301 |
| x8 | 7.428 | x8 | -7.027 | x8 | 7.284 |
| x9 | 7.235 | x9 | -6.960 | x9 | 7.276 |
| x10 | 7.365 | x10 | -6.918 | x10 | 7.414 |
| x11 | 7.228 | x11 | -6.239 | x11 | 7.262 |
| x12 | 7.063 | x12 | -7.186 | x12 | 7.105 |
| x13 | 7.040 | x13 | -8.129 | x13 | 7.090 |
| x14 | 6.987 | x14 | -8.561 | x14 | 7.052 |
| x16 | 6.947 | x16 | -8.453 | x16 | 6.984 |
| x17 | 7.009 | x17 | -7.891 | x17 | 6.950 |
| x19 | 6.968 | x19 | -8.061 | x19 | 7.032 |
| x20 | 6.974 | x20 | -7.397 | x20 | 6.967 |

**Table S24.** Average predicted global dengue incidence from 2022 to 2032.

| **Country** | **Predicted_Incidence** |
| --- | --- |
| Afghanistan | 209.38 |
| Angola | 15.21 |
| Antigua and Barbuda | 69.61 |
| Argentina | 240.78 |
| Australia | 25.49 |
| Bahamas | 76.35 |
| Bangladesh | 204.97 |
| Barbados | 41.61 |
| Belize | 225.50 |
| Benin | 82.54 |
| Bhutan | 126.70 |
| Bolivia (Plurinational State of) | 0.00 |
| Brazil | 199.62 |
| Brunei Darussalam | 205.09 |
| Burkina Faso | 58.63 |
| Burundi | 28.62 |
| Cabo Verde | 52.04 |
| Cambodia | 41.00 |
| Cameroon | 75.12 |
| Central African Republic | 0.00 |
| Chad | 0.00 |
| China | 549.25 |
| Colombia | 253.44 |
| Comoros | 57.37 |
| Congo | 84.71 |
| Cook Islands | 38.23 |
| Costa Rica | 197.01 |
| Cuba | 338.46 |
| Democratic Republic of the Congo | 0.00 |
| Djibouti | 33.85 |
| Dominica | 85.44 |
| Dominican Republic | 189.35 |
| Ecuador | 234.47 |
| Egypt | 0.00 |
| El Salvador | 227.78 |
| Equatorial Guinea | 118.82 |
| Eritrea | 0.00 |
| Ethiopia | 7.64 |
| Fiji | 132.51 |
| Gabon | 134.68 |
| Gambia | 0.00 |
| Ghana | 126.56 |
| Grenada | 56.92 |
| Guatemala | 205.16 |
| Guinea | 150.95 |
| Guinea-Bissau | 55.85 |
| Guyana | 237.71 |
| Haiti | 94.66 |
| Honduras | 152.84 |
| India | 658.10 |
| Indonesia | 270.80 |
| Jamaica | 170.58 |
| Jordan | 254.61 |
| Kenya | 78.96 |
| Kuwait | 199.66 |
| Lao People's Democratic Republic | 0.00 |
| Lebanon | 295.93 |
| Liberia | 97.25 |
| Madagascar | 88.99 |
| Malawi | 57.38 |
| Malaysia | 254.01 |
| Maldives | 361.79 |
| Mali | 91.64 |
| Mauritania | 71.36 |
| Mauritius | 108.72 |
| Mexico | 182.74 |
| Micronesia (Federated States of) | 0.00 |
| Mozambique | 18.08 |
| Myanmar | 172.73 |
| Nepal | 129.17 |
| Nicaragua | 159.66 |
| Niger | 53.59 |
| Nigeria | 107.86 |
| Niue | 0.00 |
| Oman | 100.78 |
| Pakistan | 148.13 |
| Panama | 234.88 |
| Papua New Guinea | 79.82 |
| Paraguay | 116.87 |
| Peru | 219.96 |
| Philippines | 205.06 |
| Rwanda | 126.61 |
| Saint Kitts and Nevis | 0.00 |
| Saint Lucia | 0.00 |
| Saint Vincent and the Grenadines | 0.00 |
| Sao Tome and Principe | 90.13 |
| Saudi Arabia | 338.12 |
| Senegal | 113.07 |
| Seychelles | 127.59 |
| Sierra Leone | 137.18 |
| Singapore | 259.56 |
| Somalia | 7.96 |
| South Sudan | 0.00 |
| Sri Lanka | 114.73 |
| Sudan | 43.04 |
| Suriname | 75.92 |
| Syrian Arab Republic | 60.91 |
| Thailand | 185.83 |
| Timor-Leste | 51.78 |
| Togo | 111.31 |
| Trinidad and Tobago | 81.88 |
| Uganda | 87.65 |
| United Republic of Tanzania | 0.00 |
| United States of America | 0.00 |
| Venezuela (Bolivarian Republic of) | 0.00 |
| Viet Nam | 0.00 |
| Yemen | 0.00 |
| Zambia | 53.35 |
| Zimbabwe | 26.60 |

*R^2^ = 0.81.*
